# Increased stiffness mimicking ovarian aging induces a fibroinflammatory response in follicles and impairs oocyte quality

**DOI:** 10.1093/reprod/xaaf026

**Published:** 2026-02-05

**Authors:** Sara Pietroforte, Farners Amargant

**Affiliations:** Department of Obstetrics and Gynecology, Washington University School of Medicine, St Louis, MO, United States

**Keywords:** ovarian aging, folliculogenesis, oocyte quality, stiffness

## Abstract

Reproductive aging in females is characterized by decreased ovarian reserve and oocyte quality. With aging, both mouse and human ovaries become pro-fibrotic and stiff. However, whether follicles sense and respond to microenvironmental stiffness and affect folliculogenesis and oocyte quality independent of other aging-related factors is unknown. To address this question, we cultured mouse secondary follicles in alginate hydrogels that reproduce the stiffness of reproductively young and old mice. RNA-sequencing revealed that follicles respond rapidly to increased stiffness and exhibit enrichment in genes related to inflammation and extracellular matrix remodeling. Long-term culture in stiff hydrogels resulted in reduced follicle survival, granulosa cell viability, estradiol synthesis, and oocyte quality. To begin to determine how stiffness is transmitted within the follicle, we examined transzonal projections, which mediate granulosa cell–oocyte communication and nutrient exchange. In stiff conditions, the number of transzonal projections decreased. Our findings demonstrate that follicles are highly mechanosensitive and that stiffness alone can trigger hallmarks of ovarian aging, including reduced follicle growth, reduced oocyte quality, and a fibroinflammatory phenotype potentially integrated into the oocyte via transzonal projections.

## In brief

With aging, the ovary becomes stiff, but the consequences of increased microenvironment stiffness on follicle function are unknown. Here, we demonstrate that increased stiffness impairs folliculogenesis and oocyte quality.

The ovary shows signs of age-associated cellular and tissue deterioration before any other organ in the human body (Broekmans et al., 2009; Wu et al., 2022). This reproductive aging is characterized by declines in oocyte quantity and quality and significant reductions in the ovarian-produced hormones estrogen and progesterone (Broekmans et al., 2009). Female reproductive aging both causes infertility and affects women’s overall health, as estrogen and progesterone regulate many tissues such as bones and the liver (Mills et al., 2021; Shen & Shi, 2015; Streicher et al., 2017). We and others reported that mouse and human ovaries become stiffer with advanced maternal age. This increased stiffness is mainly triggered by increased collagen deposition (fibrosis) and decreased abundance of the extracellular matrix (ECM) glycosaminoglycan hyaluronan (Amargant et al., 2020; Dipali et al., 2023; Jaeschke et al., 2025; Ouni et al., 2021). However, it is unclear whether follicles directly sense the environment and how stiffness, independent of other physiologic effects of aging such as inflammation, affect follicle development and oocyte quality.

The physical properties of the tissue have emerged as an important regulator of cell and tissue function because cells detect mechanical stiffness by a variety of receptors and trigger complex signaling pathways that result in cell proliferation, cell death, ECM production, or inflammation, which are all key processes in development and tissue homeostasis (Martino et al., 2018; Panciera et al., 2017). The ovarian follicle, the functional unit of the ovary, is a unique system in which to study biomechanics and mechanobiology since it is a contained structure consisting of an oocyte surrounded by supporting granulosa cells (Fiorentino et al., 2023; Pietroforte & Amargant, 2025). In addition, because follicles form before birth and remain in meiotic quiescence prior to ovulation, those follicles are exposed to the surrounding ovarian microenvironment for decades in humans. In fact, we know that mechanical stress regulates primordial follicle dormancy in mice (Nagamatsu et al., 2019). However, the age-associated increase in ovarian stiffness is also likely to contribute to the decrease in follicle number and oocyte quality observed with advanced maternal age for several reasons. First, multiple studies have reported a correlation between increased tissue stiffness and pathologies such as cardiovascular disease and diabetes (Mitchell et al., 2010; Sporea et al., 2016). Second, mouse follicles grow more rapidly when cultured in a low percentage 3D alginate hydrogel than when cultured in a high percentage alginate hydrogel (Hornick et al., 2012; West et al., 2007; Xu et al., 2006; Zheng et al., 2023). Finally, treatment of reproductively old mice with anti-fibrotic drugs to reduce fibrosis, and therefore stiffness, restored ovulation and increased follicle development (Amargant et al., 2025; Landry et al., 2022; Umehara et al., 2022). Although these data suggest that ovarian stiffness regulates folliculogenesis and oocyte quality, whether (and how) it can do so independent of other physiological effects of aging is unknown.

To answer this question, we developed a system to culture secondary follicles from young mice in 3D alginate hydrogels that have similar stiffness values to the young and aging ovary. Using this system, we demonstrated that follicles are highly mechanosensitive, integrating environmental cues within 3 hr of exposure, and respond by generating a fibroinflammatory response. Long-term culture in stiff hydrogels impaired follicle growth, granulosa cell viability, and oocyte quality. The decrease in oocyte quality might be mediated by a reduction in the number of transzonal projections (TZPs), which mediate communication and exchange of nutrients between granulosa cells and oocytes. Overall, we have developed a physiologically relevant system to mechanistically study the impact of microenvironment stiffness on folliculogenesis. Importantly, all the changes we report are observed in ovaries from reproductively old mice and women (Broekmans et al., 2009), suggesting that stiffness can independently contribute to the decline in oocyte quantity and quality observed in women of advanced reproductive age.

## Materials and methods

### Animals

Pre-pubertal (12–13 days) CB6F1 and CD1 mice were used to maximize the yield of secondary follicles. CB6F1 hybrids were generated by housing two Balb/c females with one C57BL/6 male per cage. CD-1 pre-pubertal mice were obtained together with a dam. All mice were purchased from Envigo (Indianapolis, IN, USA). Ovaries from 4-month-old CB6F1 female mice were used for the nanoindentation control. Mice were housed at Northwestern University’s Center for Comparative Medicine or at Washington University’s Division of Comparative Medicine under constant temperature, humidity, and light/dark cycles (12 hr light/12 hr dark) with free access to food and water. All mouse procedures were reviewed and approved by the Institutional Animal Care and Use Committee of Northwestern University (Chicago, IL, USA) or Washington University in St Louis (St Louis, MO, USA) and were performed in accordance with National Institutes of Health guidelines.

### Alginate gel preparation

Distilled phosphate-buffered saline (DPBS) without calcium and magnesium (14190250, Thermo Fisher Scientific, Waltham, MA, USA) was filtered (0.2 μm) and mixed with alginic acid solution salt from brown algae (71238, Sigma-Aldrich, St Louis, MO, USA) to create final alginate solutions of 0.5%, 2%, and 4% weight/volume (w/v). The alginate solutions were placed on a rocker at 4 °C for 24 hr, then stored at 4 °C and used within 1 month. To prepare alginate gels, a 7 μl drop of alginate solution was placed in a calcium solution (50 mM CaCl_2_ and 140 mM NaCl_2_) to crosslink the gel for 3 min, then washed at least three times with DPBS (Converse et al., 2023). To create flat alginate gels for nanoindentation, 1 mL of alginate solution was placed in a 24-mm round dish, calcium solution was added for 3 min, then gels were washed at least three times with DPBS.

### Follicle isolation, encapsulation, and growth

Follicles were cultured and analyzed as previously described (Converse et al., 2023). Briefly, ovaries from day 12 or 13 prepubertal CB6F1 and CD1 mice were placed in dissection medium containing L15, 1% fetal bovine serum (FBS), and 0.5% penicillin–streptomycin (Pen–Strep) (Gibco, Thermo Fisher Scientific). Early secondary follicles (120–130 μm) were mechanically isolated with insulin syringes without disrupting granulosa and theca cells (Babayev et al., 2022). Follicles were incubated for 1 hr at 37 °C in α-MEM-Glutamax containing 1% FBS and 0.5% Pen–Strep in a humidified atmosphere of 5% CO_2_. Only follicles with an intact basement membrane and visible and healthy oocytes surrounded by two layers of granulosa cells were selected for culture. To encapsulate the follicles, the parafilm technique was performed (Converse et al., 2023). Groups of 10 follicles were mixed with 7 μL of 0.5% or 2% w/v alginate solution. The alginate drops were crosslinked as described above. The resulting alginate beads were then incubated in α-MEM-Glutamax containing 1% FBS and 0.5% Pen-Strep for 1 hr. Finally, alginate beads were transferred to a 96-well ultra-low attachment plate (Corning, Corning, NY, USA) with one bead per well. Each well contained 100 μL of growth medium [α-MEM-Glutamax containing 3 mg/mL bovine serum albumin (MP Biomedicals), 1 mg/mL fetuin (Sigma-Aldrich), 0.1% insulin–transferrin–selenium (Thermo Fisher Scientific), and 10 mIU/mL follicle stimulating hormone (Gonal-F, EMD Serono, Inc. Rockland, MA, USA)]. Follicles were cultured at 37 °C, 5% CO_2_ for short-term culture (up to 24 hr) or for long-term culture (up to 12 days). Every other day, half of the medium was removed, frozen at −80°C for downstream analysis, and replaced with fresh medium.

### Nanoindentation

CB6F1 mouse ovaries were removed from the bursa and placed in L15 medium supplemented with 3 mg/mL polyvinylpyrrolidone (P0930, Sigma-Aldrich) and 0.5% Pen–Strep. Each ovary was then attached to a plastic Petri dish with tissue glue (PELCO^®^ Pro CA44 Tissue Adhesive, Fresno, CA, USA) and covered with warm 1x DPBS with calcium and magnesium (14040182, Thermo Fisher Scientific). Flat 0.5%, 2%, and 4% alginate gels were covered with warm 1x DPBS. Ovaries and gels were indented with a Piuma nanoindenter (Optics 11). The probe had a glass spherical tip of 100 μm in diameter in a cantilever with a spring constant of 0.5 N m^−1^. For all measurements, the indentation depth was set at a maximum of 10% of the probe diameter. A minimum of 10 indentations were performed in each ovary or gel. To obtain the Young’s modulus, the data were analyzed by using the Hertzian contact model in the Piuma software.

### Rheometry

Alginate elastic modulus was measured at 25°C with a HR-20 Rheometer (TA Instruments, New Castle, DE, USA). A parallel plate geometry (Peltier plate stainless steel, diameter 40 mm) and TRIOS software (version 5.3.0.48151; TA Instruments, New Castle, DE, USA) were used. Flat alginate gels were prepared immediately before testing as follows: 10 mL of alginate solution was placed in a 51 mm polystyrene weighing dish (ULINE, Pleasant Prairie, WI, USA), calcium solution (described above) was added for 3 min, then gels were washed at least three times with DPBS and immediately placed onto the lower plate of the rheometer. Storage moduli were determined by experiments performed with a strain amplitude of 0.01% to 10% and angular frequency of 1 rad/s. For all measurements, a minimum of 9 data points were measured on each gel. The storage moduli obtained by the TRIOS software were analyzed individually for each alginate gel (0.5%, 2%, and 4%).

### Diffusion test

Drops of alginate were crosslinked with calcium solution for 3 min as described above to produce beads, which were washed three times in PBS and incubated with a solution of Alcian blue for 3 and 5 min. Then, beads were rinsed in PBS three times, and the blue staining was visually evaluated. Beads were also bisected to determine whether the Alcian blue dye reached the centers.

### Toxicity test

The potential toxicity of 2% alginate on follicle development was evaluated by culturing encapsulated follicles in the presence of additional alginate beads. Briefly, early secondary follicles from CB6F1 mice were isolated and encapsulated in 0.5% alginate as above. They were then cultured for up to 12 days in the presence of an additional 2% alginate bead in the same well. Two technical replicates per condition were performed. Follicles cultured in 0.5% alginate with no additional bead were used as the reference.

### RNA sequencing and data analysis

Follicles were isolated from pre-pubertal (12–13 days) CB6F1 mice and cultured in 0.5% or 2% alginate beads as described above but in groups of 5 follicles per bead. Follicles were isolated at 3, 6, 12, and 24 hr and incubated for 5 min in 4 mg/mL alginate lyase. Isolated follicles were snap-frozen in a minimum volume of PBS and submitted to the Northwestern University NUSeq Core for processing and RNA sequencing in parallel. The SMART-seq mRNA kit (Takara, San Jose, CA, USA) was used to construct cDNA, and libraries were sequenced as 50 bp single-end reads on a Hiseq 4000 sequencer (Illumina, San Diego, CA, USA), with about 90 million reads in total, to produce the raw *fastq* files. Raw data were obtained for 160 follicles divided in two groups (0.5% and 2%) at four time points (3, 6, 12, and 24 hr) with four replicates of five follicles per condition (supplementary Table S1). FastQC (Babraham Bioinformatics, Cambridge, UK) was used to assess the quality of the sequencing data.

Reads were aligned to the UCSC mm10 genome (http://genome.ucsc.edu) by using STAR 2.7.0a with gene models from Ensembl (http://www.ensembl.org) and counted by using the function SummarizeOverlap of the Bioconductor/R package Genomic Features (Lawrence et al., 2013). DESeq2 (Love et al., 2014) was then used to detect differentially expressed genes (DEGs) between experimental groups, and genes with adjusted *p*-value <0.05 were considered statistically significant. The threshold of log2 fold-change (log2FC) = |2| was considered to calculate up- and down-regulated genes. Volcano plots of the statistically significant genes for each comparison were created by using the “plot” function in R [log2FC and −log10(*p*adj)].

To analyze genes with stable expression across conditions and replicates, DESeq2 was used to select genes with low log2FC and high base mean values (log2FC = 0.5 and base mean = 100). ShilyGO 0.80 (Ge et al., 2020) was used for pathway analysis of these stably expressed genes.

Gene set enrichment analysis (GSEA) was performed by WebGestalt (Liao et al., 2019), using the gene ontology (GO) database for biological function and selecting the standard parameters for analysis (organism: *mus musculus*; enrichment categories: *geneontology Biological Process noRedundant*; ID type: *ensemble gene ID*; minimum number of ID in the category: *3*; significance level: *top 20*). Up- and down-regulated genes were specifically analyzed individually in WebGestalt by using over-representation analysis and the GO database for biological function, selecting the same above-mentioned standard parameters. Additional GSEA of pathways of interest was performed with GSEA Software 4.3.3 (Mootha et al., 2003; Subramanian et al., 2005) with *Mouse MSigDB v2023.2.Mm* (October 2023) for GO biological process and/or Reactome pathways provided by the software.

### Cytokine array analysis

Media samples on day 2 of culture were collected from wells with follicles encapsulated in 0.5% and 2% alginate. For each condition, medium was collected from four independent experiments, pooled together, and analyzed with a mouse cytokine antibody array C3 (RayBio C-Series, Norcross, GA, USA) according to the manufacturer’s instructions. The medium was diluted 1:5 in the assay buffer provided in the kit, for a final volume of 1 mL sample/condition. A ChemiDoc MP Imaging System (Bio-Rad Laboratories Inc., Hercules, CA, USA) was used to detect signal. Array membranes were analyzed by a protein array analyzer (Fiji software) following the manufacturer’s instructions (http://image.bio.methods.free.fr/ImageJ/Protein-Array-Analyzer-for-ImageJ.html).

### Collagen deposition quantification

Secondary follicles were isolated from 12–13-day-old CB6F1 mice, encapsulated in 0.5% or 2% alginate, and cultured *in vitro* (*n* = 2 technical replicates per condition). At days 2, 4, 6, and 8, follicles were removed from alginate as described above and processed for histological analysis. Collagen deposition was evaluated with a trichrome stain kit (ab150686, Abcam, USA) according to the manufacturer’s instructions. Follicles were imaged with an OLYMPUS light microscope with 10x, 20x, or 60x objectives (Plympus Corporation, Center Valley, PA, USA). In Fiji software, “Color Deconvolution” was performed with Masson trichrome as vector for each follicle. The window with the green composition related to the collagen was selected, and the threshold was adjusted until the entire green area was highlighted in red. The region of interest (entire follicle area without the oocyte) was selected for each follicle, and the intensity of collagen staining was measured. Then, the total number of granulosa cells per follicle was counted by the “Cell Counter” Plugin. Collagen deposition was quantified by dividing the intensity per number of granulosa cells per follicle. Only follicles with visible oocytes were analyzed.

### Follicle survival, growth, and granular pattern

Follicles were imaged every other day by using 4x, 10x, and 20x objectives on an Evos FL Auto imaging system (Thermo Fisher Scientific). Individual follicles were tracked by analyzing their relative position inside the bead in the 4x objective images. Follicle size and survival were measured in Fiji (ImageJ, National Institute of Health, Bethesda, MD, USA). Follicle sizes were only measured until day 10 because follicles close to one another tended to fuse by day 12 of culture. Follicle growth was analyzed by calculating the average size of two perpendicular diameter measurements for each follicle. Follicles that did not grow for 2 to 4 consecutive days were considered dead. Follicle survival and growth values were plotted in Prism (GraphPad). The presence or absence of a granular appearance of the follicles was assessed in 20x objective images.

### Live/dead assay

Follicles were cultured in 0.5% or 2% alginate for 2, 4, 6, 8, or 10 days. Follicles were removed from the alginate by incubating the beads in L15 medium containing 1 mg/mL of alginate lyase for 20 min at RT. The follicles were then washed three times in L15 medium, incubated for 30 min in a live/dead viability/cytotoxicity solution containing 4 mM calcein and 8 mM ethidium homodimer-1 (L3224, Thermo Fisher Scientific) at 37 °C, 5% CO_2,_ washed three times in DPBS, and imaged at 4x, 10x, and 20x magnification with an EVOS system (calcein 494/517 nm, ethidium homodimer-1 528/617 nm).

### Estradiol measurement

The concentration of 17β-estradiol in the frozen conditioned media was measured with ELISA kits (Enzyme-Linked ImmunoSorbent Assay kit, Cayman Chemicals, Ann Arbor, MI, USA) according to the manufacturer’s instructions. Media samples collected from wells without follicles were used as negative controls. Medium was diluted 1:10 in assay buffer to ensure the readings were within the detection limits. Concentrations are reported in ng/mL.

### Oocyte analysis

Follicles were removed from the alginate on day 12 as described above. Oocytes were mechanically isolated from the follicle and imaged with an EVOS system. Oocytes with a visible germinal vesicle (GV) and without fragmentation were considered to be of good quality.

### Staining TZPs

Follicles were removed from alginate as described above, fixed with 3.8% paraformaldehyde in PBS containing 1% Triton for 20 min at 37 °C, washed three times in PBS-polyvinylpyrrolidone, and stored at 4 °C until processed for immunofluorescence. Follicles were permeabilized in PBS containing 1% BSA and 0.1% Triton X-100 for 15 min at RT, washed three times for 5 min in blocking buffer (PBS with 1% BSA and 0.1% Tween-20), then incubated with Phalloidin 633 (A22284, Thermo Fisher Scientific) for 1 hr at RT with gentle rocking. Then, follicles were washed three times for 20 min with blocking buffer and mounted onto microscope slides with Vectashield Plus Antifade mounting medium containing 4′,6′-diamidino-2-phenylindole (Vector Laboratories). Slides were imaged with a Leica TCS Sp5 confocal system with 40x or 63x objectives (Leica Microsystems, Wetzlar, Germany). In Fiji software, a background threshold was determined in each follicle by measuring the intensity in a region without actin staining. Then, a 10 μm line was drawn between the granulosa cells and the oocyte interface (zona pellucida), and a phalloidin intensity profile was generated. The number of TZPs was determined by counting the number of peaks above the background threshold.

### Statistical analyses

The normality of the data was analyzed by using the Shapiro–Wilk test, and normally distributed data were analyzed by using Student *t*-test for comparisons between two groups or one-way ANOVA for more than two groups. The statistical package in Prism 9.4 (GraphPad Software) was used for all analyses.

## Results

### The follicle transcriptome responds rapidly to a stiff microenvironment

Mouse ovaries become stiffer with advanced reproductive age. For example, instrumental indentation revealed that ovaries from 14–17-month-old mice were 2.2-fold stiffer than those from 6–12-week-old mice (4.36 ± 1.24 kPa vs. 1.98 ± 0.42 kPa) (Amargant et al., 2020). Similarly, ovaries from menopausal women were 2.2-fold stiffer than those from young women (7.1 ± 0.71 kPa vs. 3.2 ± 0.25 kPa) (Ouni et al., 2021). To investigate whether follicles have the capacity to directly integrate and respond to increased microenvironment stiffness, we performed follicle culture, a system in which follicles are cultured *in vitro* in alginate hydrogels beads (Converse et al., 2023; Green & Shikanov, 2016; West et al., 2007; Xu et al., 2006). To model the physical environment of an aging ovary, we generated alginate gels of different concentrations, including 0.5%, 2%, and 4% alginate, and we used instrumental indentation to measure their stiffness. In control experiments, ovaries from 4-month-old mice had a stiffness of 2.00 ± 0.17 kPa, confirming that the indentation procedure yielded values in the expected range. The 0.5%, 2%, and 4% alginate had stiffness values of 0.84 ± 0.16, 5.85 ± 0.28, and 11.24 ± 0.36 kPa, respectively (Figure 1A). Rheometry data confirmed the greater stiffness in 2% and 4% alginate (supplementary Figure S1). To assess whether increased alginate concentrations modified the internal structure of the gels and prevented diffusion of nutrients in the medium, we incubated 0.5%, 2%, and 4% alginate beads in the dye Alcian blue, which has a molecular weight of 1298.86 g/mol. After 3 and 5 min, all alginate beads were equally blue. We then bisected the beads and confirmed that the dye diffused across the entire bead (Figure 1B). Thus, important nutrients for follicle growth, such as glucose (180.156 g/mol) and follicle-stimulating hormone (980.2 g/mol), should be able to diffuse through 0.5%, 2%, and 4% alginate beads.

Given the above results, we chose to use 0.5% alginate to mimic the stiffness of ovaries from young mice and 2% alginate to mimic the stiffness of ovaries from reproductively old mice. We isolated secondary follicles from CB6F1 mice, cultured them in 0.5% or 2% alginate beads for 3, 6, 12, and 24 hr, and then processed them for RNA sequencing (RNA-seq) (Figure 1C; supplementary Table S1). At the 3-hr timepoint, we identified 51 DEGs between follicles in 0.5% and 2% alginate. The number of DEGs peaked at 6 hr (135 DEGs) and declined to a plateau by 12 hr (Figure 2A). GSEA identified mechanosensory behavior as one of the most enriched pathways in 2% alginate, indicating that mechanosensitive pathways in the follicle respond to the increased microenvironmental stiffness. For example, *Nrxn2*, which is involved in cell adhesion, was upregulated in the stiffer conditions (Figure 2B) (Dean & Dresbach, 2006). The Hippo pathway is one of the main mechanotransduction pathways in cells (Panciera et al., 2017), but we found no differences in expression of the Hippo-associated genes *Yap1, Taz*, or *Lats1/2* between follicles in 0.5% and 2% alginate at 3, 6, 12, or 24 hr. However, the Yap1 downstream genes *Ctgf, Ankrd1*, and *Cyr61* were significantly downregulated in 2% alginate (supplementary Figure S2), suggesting that stiffness impairs Yap1 signaling.

Volcano plots revealed that, at 3 hr, 40% of DEGs were downregulated and 60% of DEGs were upregulated in follicles cultured in 2% alginate relative to those in 0.5% alginate (Figure 2C; supplementary Table S2). At 6 hr, 70% of DEGs were downregulated and 30% were upregulated in follicles cultured in 2% alginate relative to those in 0.5% alginate (Figure 2F; supplementary Table S3). We then performed GO analysis for the down- and up-regulated genes to identify pathways affected by stiffness. At 3 hr, genes down-regulated in follicles cultured in 2% alginate were associated with phagosome maturation, morphogenesis, and development, including Noggin (*Nog*), which is an antagonist to bone morphogenetic proteins (Glister et al., 2019; McMahon et al., 1998) (Figure 2D). At 3 hr, up-regulated genes in 2% alginate were associated with the immune response mediated by chemokines and interleukin-1, as well as negative regulation of response to external stimuli, such as forkhead box F1 (*Foxf1*), a transcription factor associated with matrix remodeling and cell junction organization (Flood et al., 2019; Malin et al., 2007; Nilsson & Kannius-Janson, 2016) (Figure 2E). To gain more insights into these inflammatory pathways, we performed GSEA. Multiple inflammatory-associated pathways were enriched in follicles cultured in 2% alginate, including positive regulation of T helper cells, humoral immune response mediated by circulating immunoglobulins, eosinophil chemotaxis, and neutrophil migration (supplementary Figure S3). Some of the most upregulated genes were caspase recruitment domain family member 9 (*Card9*), which is involved in NF-kappa B activation and cell apoptosis (Bertin et al., 2000); complement factor I (*Cfi*), involved in regulation of the complement cascade (Roversi et al., 2011); C-C motif chemokine ligand 3 (*Ccl3*), involved in monocyte/macrophage chemotaxis (Metzemaekers et al., 2020); and NCK-associated protein 1 like (*Nckap1l*), involved in neutrophil migration (Cook et al., 2020).

At 6 hr, metabolic-associated processes were the most down-regulated pathways in follicles cultured in 2% alginate (Figure 2G), while up-regulated genes were associated with biological processes such as apoptosis, inflammation, and cellular adaptation to the environment (Figure 2H). No differences were noted in gene expression between follicles in 0.5% alginate and 2% alginate at 12 and 24 hr of culture, suggesting that follicles transiently adapt to the stiff microenvironment (supplementary Figure S4A–D). We identified 10,798 genes that did not differ in expression across all comparisons and conditions. In GO analysis, these genes were associated with biological processes important for oocyte maturation, cell division, metabolic processes, and cell survival (supplementary Figure S4E). We conclude that follicles are highly mechanosensitive, rapidly integrating the cues from their environment. We show that a physiologically relevant stiff microenvironment alters metabolism and inflammation pathways, which are well-known regulators of follicle development and growth.

### A stiff microenvironment increases expression of genes associated with ECM remodeling

To examine the dynamics of gene expression changes, we compared DEGs at 3 and 24 hr in the 0.5% and 2% conditions. We first removed the 51 DEGs between the 0.5% and 2% conditions to obtain the most homogeneous population at 3 hr. We identified 1,282 DEGs between follicles cultured in 0.5% alginate at 24 hr and those at 3 hr (supplementary Table S4). Identified molecular pathways were associated with protein and ion binding, and biological pathways were associated with metabolic processes and response to stimuli. Among the 39% of DEGs that were down-regulated at 24 hr relative to 3 hr (Figure 3A), GO analysis revealed genes involved in metabolic processes such as amino acid synthesis or hormone-related processes (Figure 3B). A subset of the up-regulated genes classified in GO were known as “benzene and ether metabolism, peptidyl-arginine modification, and detoxification.” Because these GO pathways did not match the molecular and biological processes results, we investigated the genes in each GO term. The benzene and ether metabolism term included genes such as acetyl-coenzyme A acyltransferase 1B, glutathione S-transferase, hydroxyacid oxidase 2, and phospholipase A2, which are involved in fatty acid metabolism or acetyl CoA-transferase activity (Figure 3C). All of these genes are related to metabolic processes and energy production, which are essential for follicle growth.

We next analyzed time-dependent gene expression changes in 2% alginate and identified 1,029 DEGs in follicles between 3 and 24 hr (supplementary Table S5). These genes were associated with protein and ion binding, metabolic pathways, and response to stimulus pathways as in the 0.5% condition. Of the 36% of DEGs that were down-regulated at 24 hr (Figure 3D), GO analysis revealed genes that were associated with response to hormones, metabolic processes, and development (Figure 3E). These included eukaryotic translation initiation factor 4E binding protein 1 (*Eif4bp1*), Kruppel-like factor 5 (*Klf5*), prostaglandin-endoperoxide synthetase 2 (*Ptgs2*), and early growth response 3 (*Egr3*). The up-regulated genes were associated with matrix remodeling, including collagen metabolic processes, and connective tissue development (Figure 3F). Analysis of the ECM-associated genes by GSEA revealed that pathways related to ECM matrix organization were enriched in 2% alginate. This included 91 significant genes such as desintegrin and metalloproteinase domain 8 (*Adam8*), integrin subunit alpha 2 b (*Itga2b*), collagen type XI alpha 1 chain (*Col11a1*), fibrillin 2 (*Fbn2*), and elastin microfibril interfacer 3 (*Emilin* 3) (Figure 3G). The most abundant ECM gene families included 34 genes related to collagen formation (Figure 3H and I). Together, these results suggest that a microenvironment that mimics the stiffness of a reproductively old ovary triggers follicles to express genes that could remodel the ECM within the follicle.

### A stiff microenvironment induces an intrafollicular fibroinflammatory phenotype

In our RNA-seq data, the two most prominent pathways triggered by a stiff environment were inflammation and collagen deposition. To determine whether the RNA expression differences in inflammatory genes were reflected in proteins, we collected media at day 2 of culture from follicles in 0.5% and 2% alginate and assayed them on a cytokine array. Twelve of the 62 cytokines in the array were more than 1.5-fold more abundant in medium from follicles in 2% alginate than in medium from follicles in 0.5% alginate (Figure 4A–C). Some of these cytokines were also upregulated in our RNA-seq data, such as IFN-γ. To determine whether the RNA expression differences in collagen deposition genes were reflected in proteins, we stained follicles with Trichrome up to day 8 of culture. In both alginate concentrations, we observed collagen foci in the follicles. In 0.5% alginate but not in 2% alginate, collagen content declined over the 8 days (Figure 4D and E). Overall, these results confirm that follicles respond to stiffness by generating an intrafollicular fibroinflammatory phenotype.

### Increased stiffness impairs follicle survival and growth

To explore whether the follicular response to microenvironmental stiffness altered follicle development, follicle function, and oocyte quality, we cultured early secondary follicles in 0.5% or 2% alginate for 12 days and examined follicle survival. Given reports of mouse strain-dependent differences in early follicle growth patterns (Canning et al., 2003), we performed these experiments with follicles from both the hybrid CB6F1 mouse strain and the outbred CD1 strain (Figure 1C). On day 12 of culture, 100% of CB6F1 follicles and >80% of CD1 follicles cultured in 0.5% alginate were viable. In contrast, only 6.66% of CB6F1 and 30% of CD1 follicles cultured in 2% alginate were viable (Figure 5A and B; supplementary Figure S5A and B). We next analyzed the growth rates of the follicles that survived during the first 10 days of culture. On day 0, follicles from CB6F1 mice were 122.0 ± 2.48 μm in diameter, and those from CD1 mice were 125.3 ± 1.88 μm. Follicles cultured in 0.5% alginate showed significant growth by day 6 (CB6F1, 171.6 ± 13.58 μm; CD1 200.4 ± 23.01 μm) and reached 247.6 ± 13.76 μm (CB6F1) and 226.9 ± 8.69 μm (CD1) by day 10 of culture (Figure 5C and D; supplementary Figure S6A and B). In 2% alginate, follicles stopped growing by day 6. Follicles from CB6F1 mice reached a final size of 157.5 ± 10.33 μm in 2% alginate (Figure 5C and D). Follicles from CD1 reached a final size of 160.8 ± 4.03 μm in 2% alginate (supplementary Figure S6A and B). To determine whether the observed decrease in follicle growth in 2% alginate was due to the increased stiffness, the toxic effect of higher alginate concentrations, or both, we co-incubated 0.5% alginate beads containing follicles with additional 2% alginate beads. Growth of follicles cultured in the presence of additional 2% alginate beads was similar to that of follicles in the absence of additional 2% alginate beads (supplementary Figure S5C). Together, these results indicate that stiff microenvironments slow follicle growth during the active expansion phase of folliculogenesis and that this effect is not due to alginate toxicity.

We wondered whether the lack of follicle growth in stiff hydrogels reflected poor growth of granulosa cells, oocytes, or both and thus analyzed the size of these two cell types separately. On day 0, granulosa cells from CB6F1 mice were 64.0 ± 4.16 μm in diameter, and those from CD1 mice were 65.2 ± 2.98 μm. Granulosa cells from CB6F1 mice in 0.5% alginate reached a maximum size of 133.0 ± 21.66 μm on day 10, and those from CD1 mice reached a maximum size of 160.0 ± 6.71 μm. In 2% alginate, growth of granulosa cells from both strains was significantly impaired. On day 10, granulosa cells from CB6F1 had reached a final size of 107.4 ± 7.26 μm in 2% alginate (Figure 5E). Granulosa cells from CD1 mice reached a final size of 103.4 ± 6.74 μm in 2% alginate (supplementary Figure 6C). Additionally, beginning on day 4 of culture in 2% alginate, some granulosa cells from both strains began to appear granular, a marker of cellular stress, and the percentage of granulated granulosa cells increased with higher alginate concentrations (supplementary Figure S6E–H). However, no major differences were observed in oocyte size, except for those from CB6F1 mice at day 10 (0.5%, 72.8 ± 2.49 μm; 2%, 62.8 ± 2.44 μm; Figure 5F; supplementary Figure S6D). Altogether, these data show that the increased microenvironment stiffness significantly impairs granulosa cell expansion and follicle growth.

### The somatic compartment of the follicle is impaired by increased stiffness

To determine how the stiffer environment affected granulosa cell expansion, we performed a live/dead assay. As expected, most of the follicles cultured in 0.5% alginate were alive on day 10 as indicated by the green fluorescent signal (Figure 6A; supplementary Figure S7A). In contrast, a red fluorescent signal (cell death marker) was present on day 8 in follicles cultured in 2% alginate, and the proportion of dead cells increased substantially during the culture period (Figure 6A; supplementary Figure S7A). The first cells to die were those in the external part of the follicle. By the end of the culture period in 2% alginate, most granulosa cells and oocytes were dead. Consistent with the death noted in the granulosa cells, follicles cultured in 2% alginate produced less estradiol than follicles cultured in 0.5% alginate. By day 10 of culture, estradiol concentrations were 533-fold lower in CB6F1 follicles and 35.1-fold lower in CD1 follicles cultured in 2% alginate than in follicles cultured in 0.5% alginate (Figure 6B; supplementary Figure S7B).

To determine whether increased stiffness also resulted in decreased oocyte quality, we removed follicles from the alginate on day 12 and mechanically isolated the oocytes. Because follicles in 2% alginate gels did not reach 180 μm in diameter, we did not induce *in vitro* oocyte maturation and instead only examined whether oocytes reached the GV stage. In 0.5% alginate, 73.5 ± 4.91% and 76.4 ± 4.61% of the isolated CB6F1 and CD1 oocytes, respectively, were classified by morphological criteria as good-quality GV oocytes. However, in 2% alginate, only 7.9 ± 3.96% and 31.1 ± 6.87% of CB6F1 and CD1 oocytes, respectively, were of good quality (Figure 6C and D; supplementary Figure S7C and D). Importantly, oocytes isolated from follicles cultured in 0.5% alginate beads with an additional 2% alginate bead were of similar quality to those incubated without an additional bead (supplementary Figure 5C and D). This result indicated that the decreased oocyte quality in 2% alginate was not due to toxicity from the alginate. Together, these results suggest that a stiff microenvironment impairs granulosa function and viability. The cells in closest contact with the environment are affected first, and the negative impact is then transmitted to the internal layer of granulosa cells and the oocyte, leading to impaired oocyte quality.

### TZPs are dysregulated in response to increased stiffness

We wondered how the impact of increased stiffness is transmitted from granulosa cells to oocytes. Granulosa cells communicate with and provide essential nutrients to the oocyte via TZPs, which emanate from granulosa cells and connect to oocytes via gap junctions (Doherty et al., 2022). Older mice have fewer TZPs and lower-quality oocytes than younger mice, (El-Hayek et al., 2018), so we hypothesized that increased stiffness could impair TZPs. To test this idea, we collected secondary follicles from young CB6F1 and CD1 mice, randomly distributed them between 0.5% and 2% alginate gels, and cultured them for 2 days. We analyzed TZPs after 2 days because follicle expansion hinders visualization of TZPs by confocal microscopy. Freshly isolated follicles served as controls. Follicles from both mouse strains in 2% alginate had significantly fewer TZPs than follicles in 0.5% alginate (Figure 6E and F; supplementary Figure S7E and F). Overall, increased stiffness induces follicular changes that result in fewer TZPs, which may explain the decreased oocyte quality.

## Discussion

In this study, we leveraged a robust method to investigate the impact of increased stiffness, independently of other factors associated with aging, on follicle development and oocyte quality. Using this approach, we demonstrated that follicles rapidly integrate the physical cues of the environment, which led to decreased follicle growth, impaired follicle function, granulosa cell death, and reduced oocyte quality. All of these are well-known features of mouse and human ovarian aging (Broekmans et al., 2009; Lliberos et al., 2021; Machlin et al., 2021; Navarro-Pando et al., 2021; Zhang et al., 2020). Follicles are highly mechanosensitive, as we detected numerous DEGs within 3 hr of culture in stiff hydrogels. Enriched transcripts were related to inflammation and ECM remodeling. Our finding that the number of TZPs was decreased in stiffer environments suggests that stiffness impairs oocyte quality by impairing the granulosa–oocyte bidirectional communication system. Together, the data presented here support the hypothesis that increased stiffness in the ovarian environment, which is a hallmark of the aging ovary, directly impairs follicle development and oocyte quality, thus microenvironment stiffness is a new driver of age-associated ovarian dysfunction (Figure 7).

The RNA-seq and cytokine analysis revealed that, upon exposure to a stiff microenvironment, follicles developed a fibroinflammatory phenotype. This finding is consistent with RNA-seq data revealing that inflammation genes were more highly expressed in follicles isolated from reproductively old mice than in those from young mice (Duncan et al., 2017). In addition, follicular fluid obtained from women of advanced maternal age is enriched in pro-inflammatory cytokines (Machlin et al., 2021). Therefore, the stiff environment in the ovary may contribute to the generation and maintenance of the inflammatory profile observed in follicles from reproductively old mice and women of advanced maternal age. Although we did not assess whether the inflammatory response was triggered by the theca, granulosa cells, or the oocyte, we suspect granulosa cells were responsible for two reasons. First, they are the outermost cells of the follicle and are in contact with the ovarian microenvironment. Second, granulosa cells have been proposed to possess immune signaling capabilities (Hashim et al., 2025). Immune-privileged structures such as the follicle, which is enclosed by the basement membrane, can release similar cytokines and chemokines typical of immune cells such as leukocytes (Hashim et al., 2025; Typiak & Żurawa-Janicka, 2024). Some of the DEGs we identified in our RNA-seq analysis encoded chemokines that attract immune cells, such as eosinophils, suggesting that stiffness can promote granulosa cell immune signaling capabilities. Future studies are needed to definitively determine which cell types drive the fibroinflammatory phenotype and the molecular pathways involved.

Our transcriptomic data also revealed that genes related to ECM remodeling and collagen production were higher in follicles cultured in 2% alginate than in those cultured in 0.5% alginate. The collagen foci that we identified in our histologic analysis resemble the “focimatrix” observed between granulosa cells in mouse, bovine, sheep, and human follicles (Huet et al., 1997; Irving-Rodgers et al., 2004; 2010; Irving-Rodgers & Rodgers, 2000; Nakano et al., 2007; Yamada et al., 1999). Similar observations have been reported in models of premature ovarian aging in cattle (Modina et al., 2014). In sheep, collagen, fibronectin, and laminin within the granulosa cell focimatrix increase with follicular atresia (Huet et al., 1997). In humans, focimatrix abundance may increase with advanced maternal age in response to tissue stiffness, thereby contributing to the age-related increase in follicular atresia. Overall, our data challenge the idea that the stroma is the major contributor to the fibroinflammatory phenotype in the aging ovary. Instead, we propose that the follicle contributes to fibroinflammation, creating a stiffness–inflammation positive feedback loop. Further studies to investigate the pathways that link increased stiffness with the development of a fibroinflammatory response in the follicle are warranted.

We were interested in determining whether microenvironment stiffness had any effect on the mechanosensitive proteins YAP and TAZ. When other cell types are exposed to stiff environments, these transcriptional co-activators are transported to the nucleus where they bind to TEAD transcription factors to regulate essential biological processes such as cell proliferation, migration, and differentiation (Clark et al., 2022; Panciera et al., 2017). A common upstream regulator of YAP/TAZ is the Hippo pathway. In follicles, the Hippo/YAP pathway regulates follicle activation, granulosa cell proliferation, and oocyte cytoplasmic maturation (Clark et al., 2022; Kawamura et al., 2013; Lv et al., 2019; Plewes et al., 2019; Yu et al., 2016). Additionally, single nucleotide polymorphisms in *Yap1* have been identified in patients with polycystic ovarian syndrome (Hayes et al., 2015; Lidaka et al., 2022). In contrast to what we anticipated, we did not observe differences in expression of the core components of the Hippo signaling pathway (*Lats1/2, Yap1, and Taz*) between follicles in 0.5% alginate and those in 2% alginate. Instead, we detected stiffness-associated decreased expression of some of the Yap1 downstream genes (*Ctgf*, *Ankrd1*, and *Cyr61*). This finding contrasts with observations in cell culture that a stiff environment induces nuclear transport and activation of *Yap1*. One possibility is that, in the follicle, *Yap1* activity is inhibited to limit granulosa cell proliferation because follicles in 2% alginate are exposed to a confined environment. Consistent with this idea, our transcriptional analysis detected stiffness-induced upregulation of mechanosensory genes, including *neurexin 1* and *2*, which are involved in cell–cell communication (Guzman et al., 2024; Trotter et al., 2019), and the transcription factor ETV1, which regulates cell growth, migration, and differentiation (Okazawa et al., 2016; Ziojla et al., 2025). Future efforts will be directed at exploring the interplay between increased stiffness and the activation or inhibition of these pathways in the follicle.

We found that long-term exposure to increased stiffness inhibited follicle growth. Granulosa cells were the first to be affected by the stiff microenvironment, as evidenced by reduced viability and increased cell death. This finding is not surprising, as granulosa cells are in contact with the basement membrane and extra-follicular environment. In fact, theca cells, which surround the follicles, can exert a compressive stress on the follicles to regulate granulosa cell proliferation and growth (Biswas et al., 2025). Previous studies have revealed effects of aging on granulosa cells. For example, ovaries from both reproductively old mice (Duncan et al., 2017) and from women of advanced maternal age (Santoro et al., 2003) contain follicles with a smaller granulosa cell layer. Our findings that increased ovarian stiffness impaired granulosa cell viability and follicle size supports the idea that a stiff microenvironment can mediate the age-associated decline in granulosa cell viability, and therefore, oocyte quality, in ovaries from reproductively old mice. To begin to examine the mechanism by which the effects of tissue stiffness are transmitted from the granulosa cells to the oocyte, we examined TZPs. TZPs are actin-based filaments that mediate oocyte–granulosa communication and participate in delivering essential nutrients, such as pyruvate, from the granulosa cells to the oocyte (Doherty et al., 2022). Additionally, the oocyte uses TZPs to signal to granulosa cells and regulate the rate of follicle development (Eppig et al., 2002). We found that follicles cultured in stiff hydrogel had significantly fewer TZPs than follicles cultured in soft hydrogel. Thus, TZPs, or the loss of TZPs, may transmit the stiffness signals from granulosa cells to the oocyte.

Our study had two major strengths. First, our *ex vivo* follicle culture system allowed us to explore the impact of increased stiffness on folliculogenesis without the contribution of other age-associated factors that would be present in *in vivo* experiments. Second, we tested the effect of stiffness in two mouse strains, the hybrid CB6F1 strain and the outbred CD1 strain. Follicles from both strains were severely affected by microenvironment stiffness, though we detected a few strain-specific differences. For example, follicles from the CD1 strain in 2% alginate showed a reduction in expansion earlier than CB6F1 follicles. However, the granulated morphology appeared earlier in follicles from CB6F1 mice. These strain-associated differences suggest that some strains are more resistant to changes in the mechanical properties of the ovarian microenvironment and have longer reproductive lifespans.

Together, the work presented here demonstrates that the increased stiffness observed in the aging ovary has a direct impact on ovarian function and oocyte quality. Our findings suggest a model (Figure 7) in which the follicle quickly responds to mechanical signals, leading to a fibroinflammatory environment and reduced granulosa cell number and viability. This, in turn, may impair oocyte quality by disrupting the bidirectional communication between the granulosa cells and the oocyte via TZPs. Importantly, all of the phenotypes we observed in response to 2% alginate have been observed in ovaries from women of advanced maternal age, suggesting that ovarian stiffness alone can trigger an aging phenotype in the follicle. In the long term, these findings may open the door to treatment, such as ovarian drilling (Ferraretti et al., 2001; Kawamura et al., 2013), that can reduce stiffness or mechanical stress to improve ovarian function in conditions such as polycystic ovarian syndrome, primary ovarian insufficiency, or ovarian aging.

## Supplementary Material

Suppl Tables

Suppl Figures

Supplementary material is available at *Reproduction* online.

## Figures and Tables

**Figure 1:**
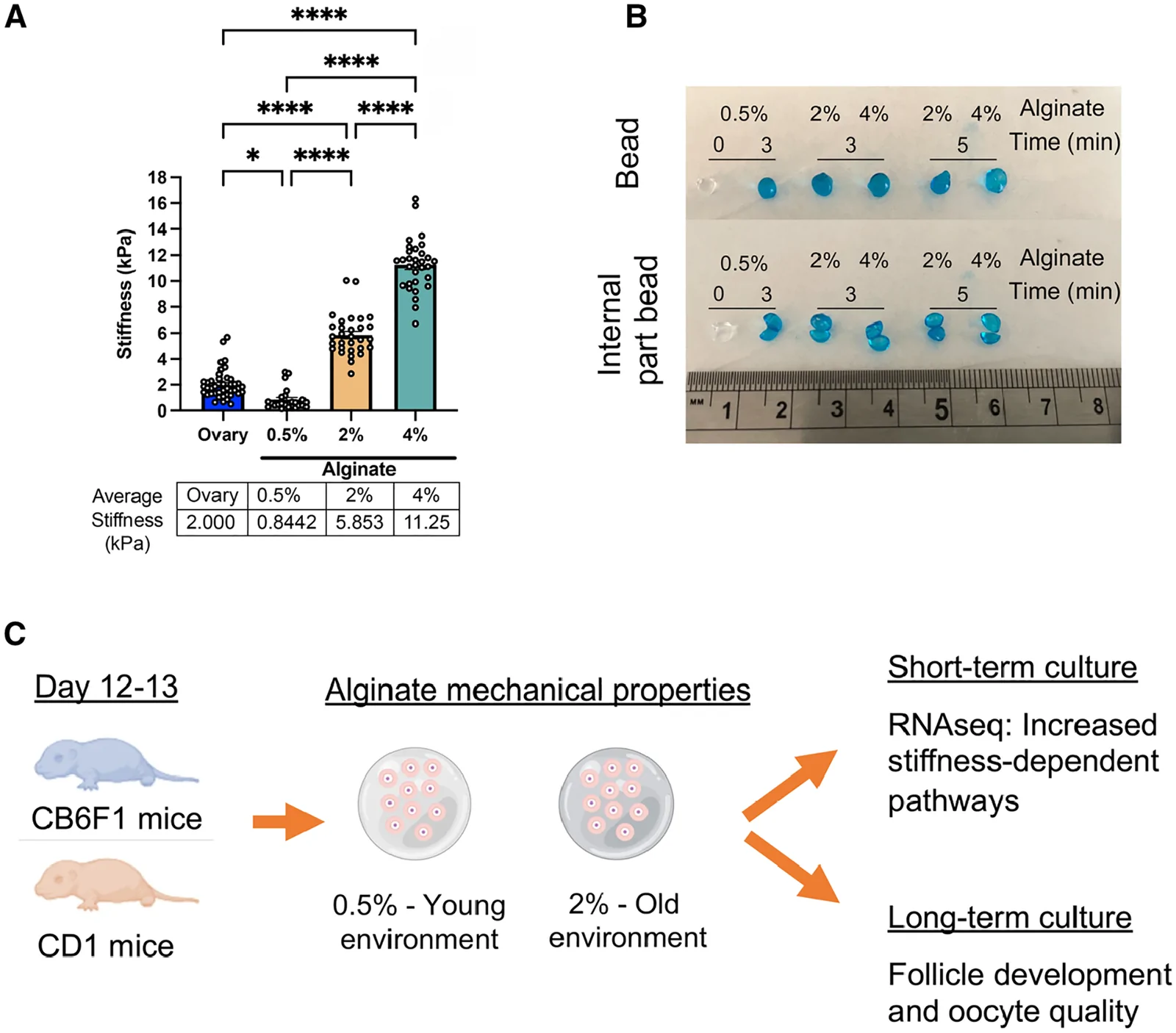
Alginate hydrogels recapitulate the stiffness of reproductively young and old mouse ovaries. (A) Quantification of the stiffness of whole ovaries from reproductively young CB6F1 mice and 0.5%, 2%, and 4% w/v alginate gels. *p < 0.005, ****p < 0.0001. (B) Representative images of 0.5%, 2%, and 4% w/v alginate gels before and after staining with Alcian blue for the indicated times. Top image, whole beads; bottom image, bisected beads. (C) Schematic representation of the *in vitro* system used to examine the impact of microenvironment stiffnesses on follicle development and oocyte quality.

**Figure 2:**
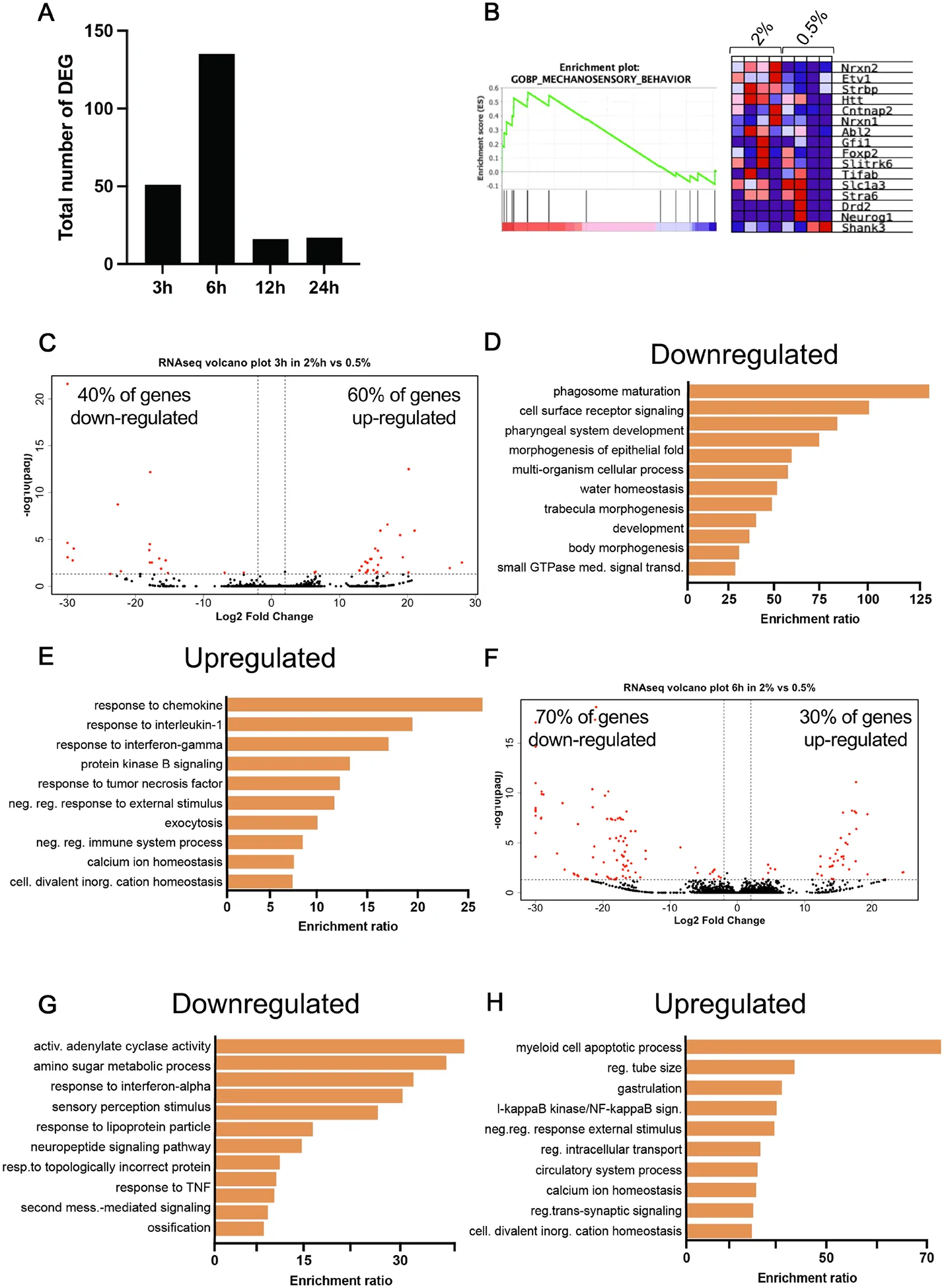
Follicles rapidly respond to stiffness and express genes involved in inflammation. (A) Graph showing the total number of differentially expressed genes (DEGs) between the 0.5% and 2% alginate-encapsulated follicles at the indicated time points. (B) Graph showing the gene set enrichment analysis of the mechanosensory behavior pathway and its associated genes. Red indicates over-represented genes, and blue indicates under-represented genes. (C) Volcano plot showing DEGs in follicles cultured in 2% alginate compared to those cultured in 0.5% at 3 hr. (D, E) Graphs showing gene ontology (GO) analysis of down-regulated (D) and up-regulated (E) DEGs in 2% alginate compared to 0.5% at 3 hr. (F) Volcano plot showing DEGs in follicles cultured in 2% alginate compared to those cultured in 0.5% at 6 hr. (G, H) Graphs showing GO analysis of down-regulated (G) and up-regulated (H) DEGs in 2% alginate gels compared to 0.5% at 6 hr.

**Figure 3:**
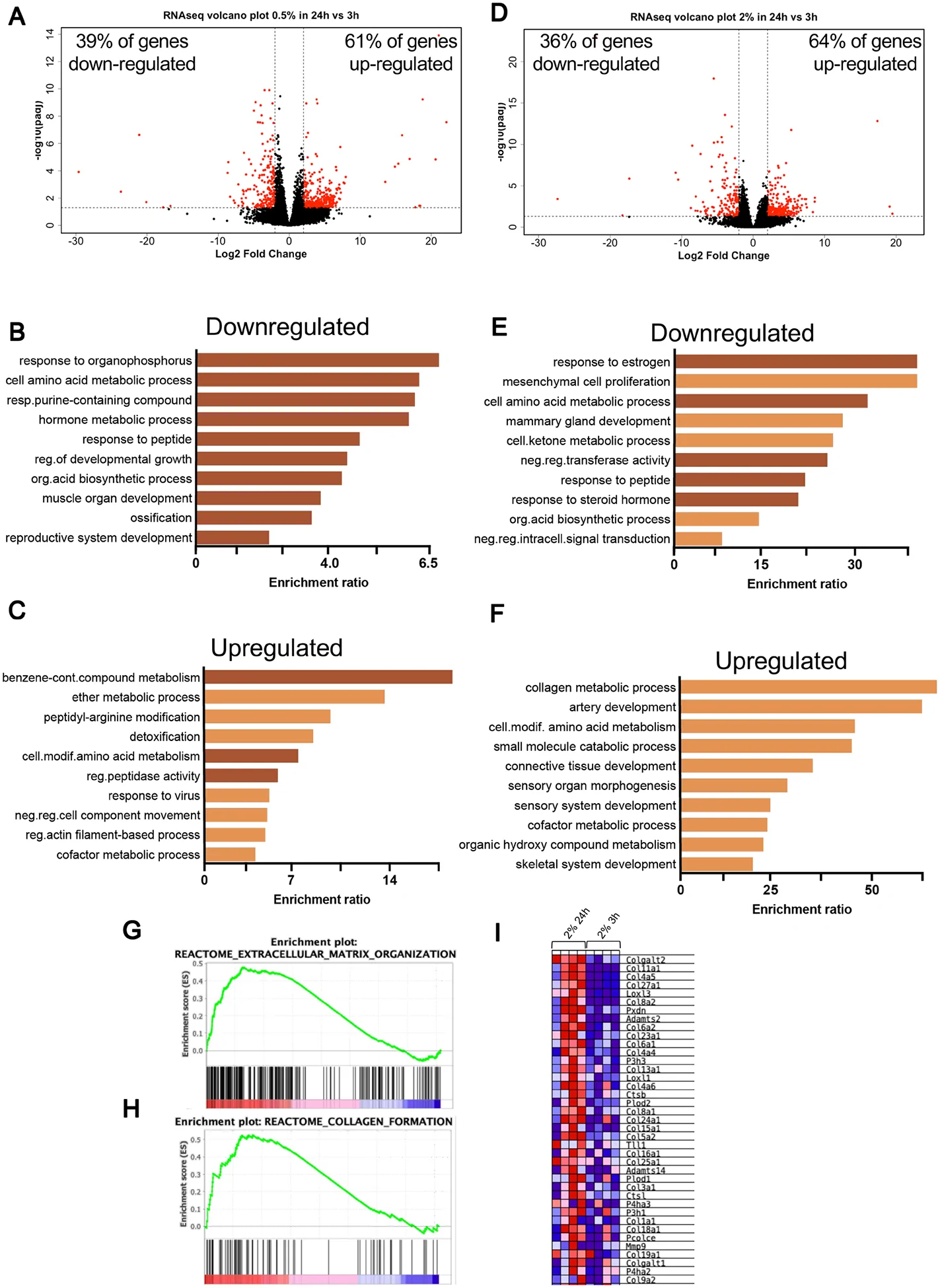
Microenvironment stiffness changes the follicle transcriptome in a time-dependent manner. (A) Volcano plot showing DEGs in follicles cultured in 0.5% alginate gels at 24 hr vs. 3 hr. (B, C) Graphs showing gene ontology (GO) analysis of down-regulated (B) and up-regulated (C) DEGs at 24 hr compared to 3 hr in 0.5% alginate. (D) Volcano plot showing DEGs in follicles cultured in 2% alginate at 24 hr vs. 3 hr. (E, F) Graphs showing GO analysis of down-regulated (E) and up-regulated (F) DEGs at 24 hr compared to 3 hr in 2% alginate. (G) Gene set enrichment analysis (GSEA) plot demonstrating up-regulated expression of the extracellular matrix organization pathway in 2% alginate at 24 hr relative to 3 hr. (H, I) GSEA plot demonstrating up-regulated expression of the collagen formation pathway (H) and its associated significantly enriched genes (I). Only genes that are significantly different (*p* < 0.05) are shown in the cluster plots. Red indicates over-represented genes and blue indicates under-represented genes.

**Figure 4:**
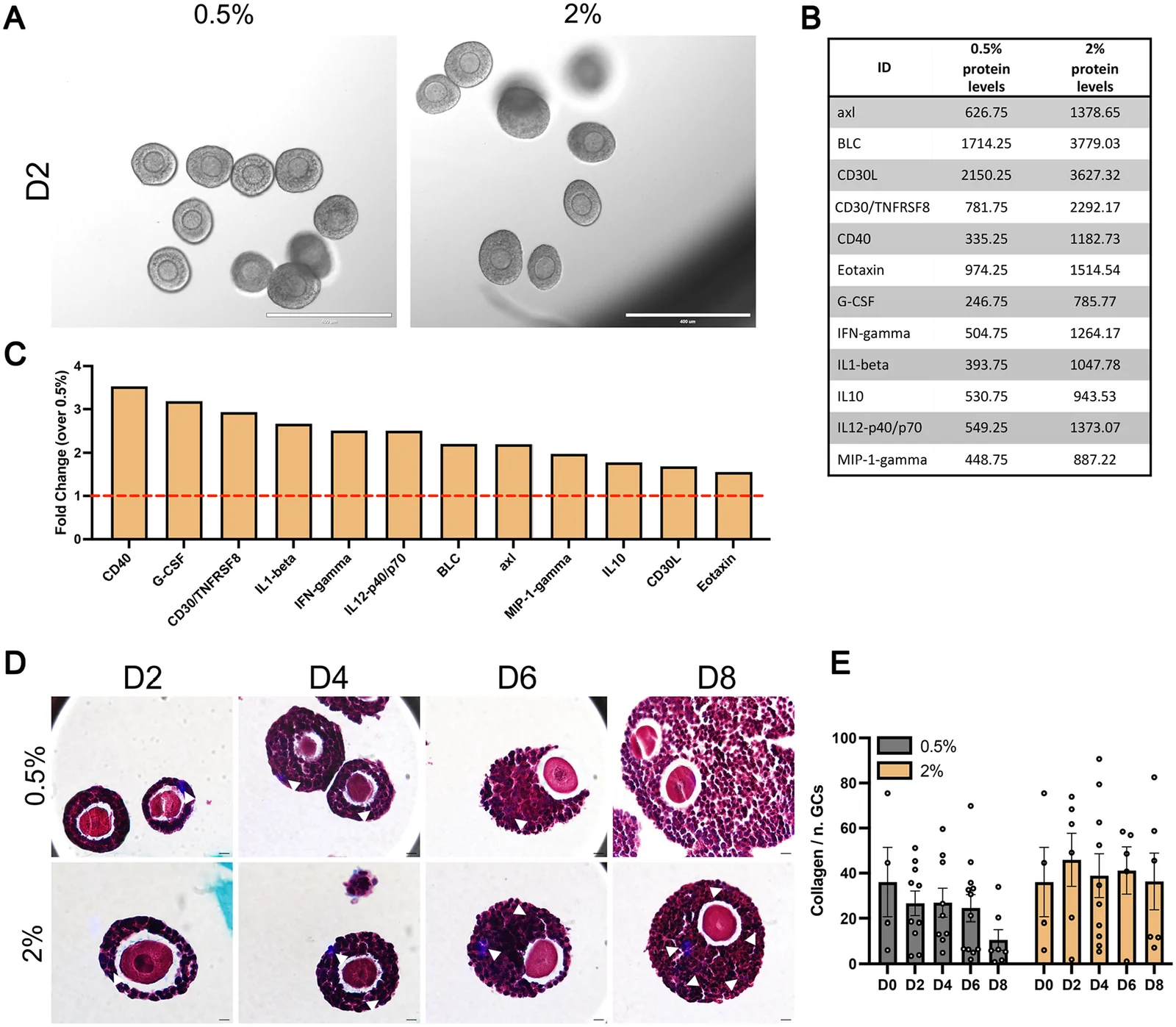
Microenvironment stiffness induces a fibroinflammatory phenotype in the follicle. (A) Images showing follicles cultured for 2 days (D2) in 0.5% and 2% alginate. Scale bar, 400 μm. (B, C) Table (B) and graph (C) showing abundance of cytokines that are ≥1.5-fold enriched in 2% alginate compared to 0.5% alginate. The red line marks the 1.5-fold change threshold. *n* = 4 independent experimental replicates were performed (10 follicles per condition for a total of 80 follicles processed). (D) Representative images of follicles cultured up to 8 days in a 0.5% or 2% alginate and stained with Trichrome assay reagents (collagen is blue). Scale bar, 100 μm. (E) Graph showing the intensity of collagen staining per number of granulosa cells in follicles cultured in 0.5% and 2% alginate. *n* = 2 independent replicates, with 4 to 12 follicles analyzed per condition.

**Figure 5:**
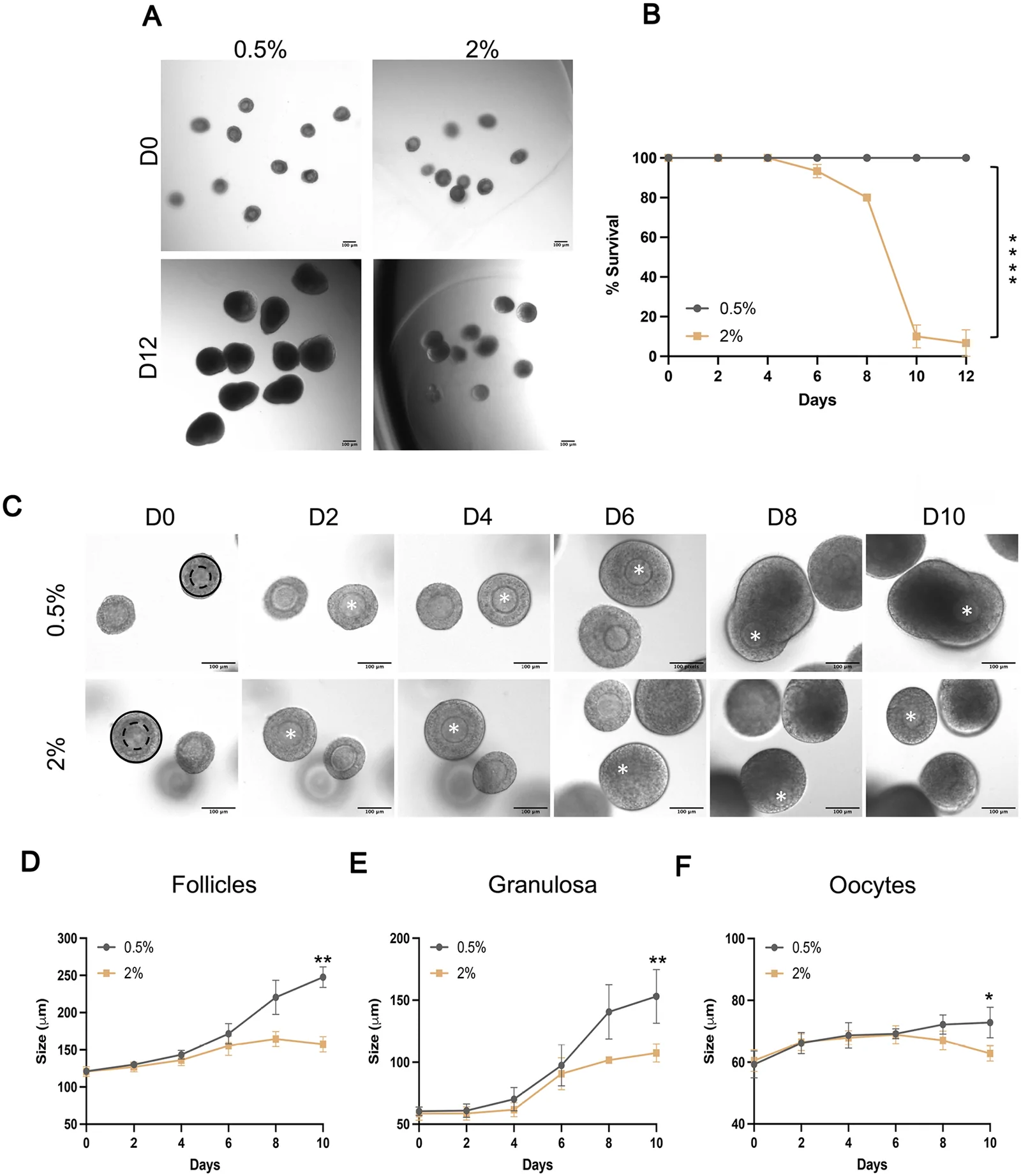
Increased microenvironment stiffness impairs follicle viability and growth *in vitro*. (A) Representative transmitted light images of CB6F1 follicles cultured in 0.5% and 2% alginate beads at day 0 (D0) and day 12 (D12). (B) Graphs showing the CB6F1 follicle survival percentage during the culture period. *n* = 3 independent experiments (*n* = 30 follicles per condition). ****p < 0.0001. (C) Representative images of CB6F1 follicles grown in the indicated alginate concentrations over 10 days. Within the follicles, granulosa cells are marked in black and oocytes are marked with white asterisks. (D–F) Graph showing the sizes of CB6F1 follicles (D), granulosa cells (E), and oocytes (F) during 10 days of culture. At least *n* = 3 independent experiments, with 10 follicles each, were performed. Scale bar, 100 μm. *p < 0.05, **p < 0.01.

**Figure 6:**
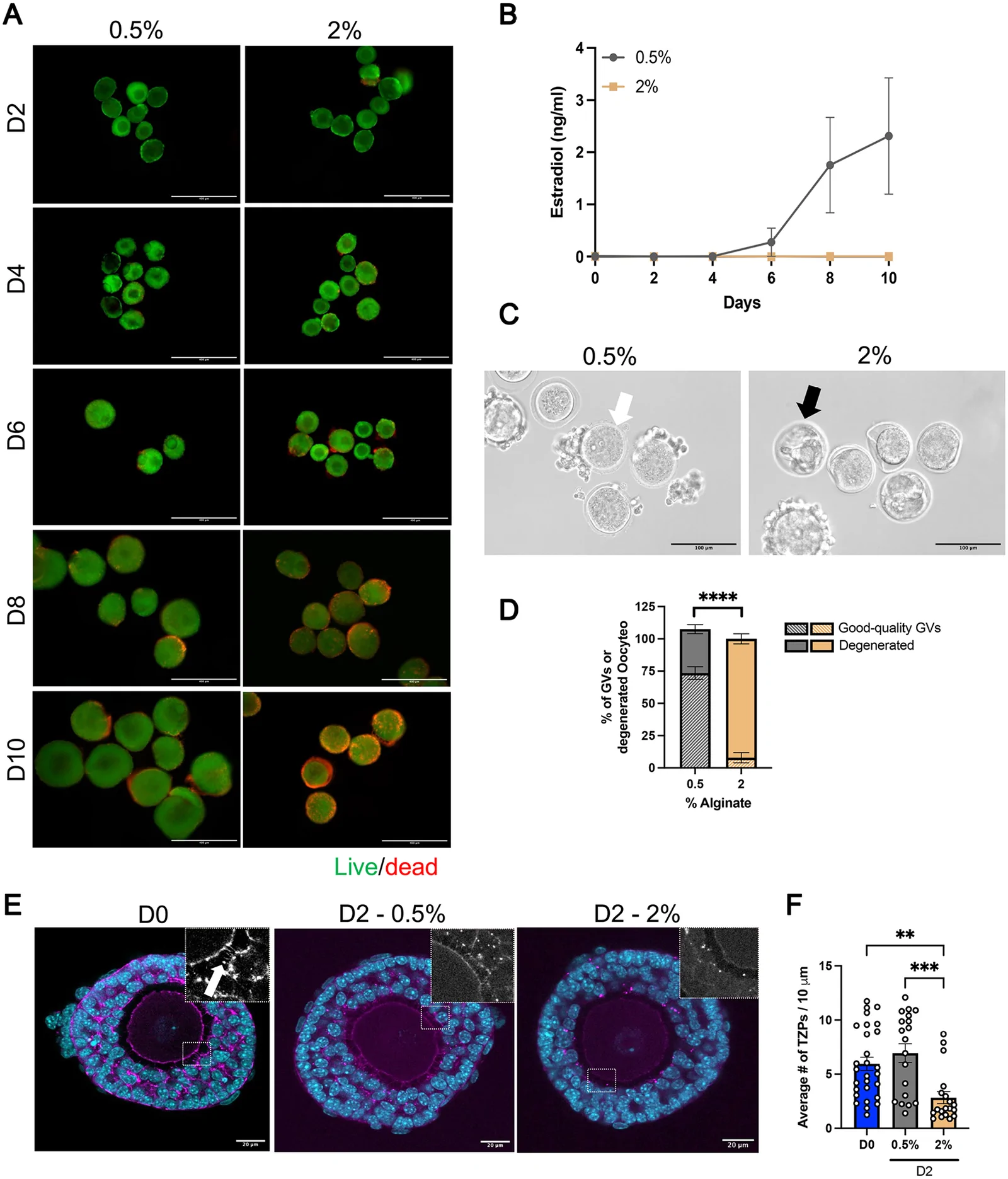
Granulosa cell survival and function and oocyte quality are severely affected by culture in a stiff microenvironment. (A) Representative images of CB6F1 follicles cultured in 0.5% and 2% alginate analyzed with a live (green)/dead (red) assay. n ≥ 10 follicles per condition. Scale bar, 400 μm. (B) Quantification of estradiol concentration in media from CB6F1 follicles. n ≥ 3 independent experiments, with 10 follicles each. (C) Representative images of CB6F1 oocytes isolated from follicles cultured in 0.5% and 2% alginate for 12 days. White arrow indicates good-quality oocyte. Black arrow indicates a degenerated oocyte. (D) Quantification of the percentage of CB6F1 germinal vesicle-stage oocytes (GVs) that were of good quality or degenerated. n ≥ 3 independent experiments, with 10 follicles each. (E) Representative images of CB6F1 follicles at day 0 (D0) and day 2 (D2) of culture stained with DNA dye (cyan) and Phalloidin (magenta). Insets show magnified images in greyscale. White arrow indicates transzonal projections (TZPs). Scale bar, 20 μm. (F) Graph showing the number of TZPs per 10 μm. n ≥ 3 independent experiments with 13 to 17 follicles per condition. **p < 0.01, ***p < 0.001, ****p < 0.0001.

**Figure 7:**
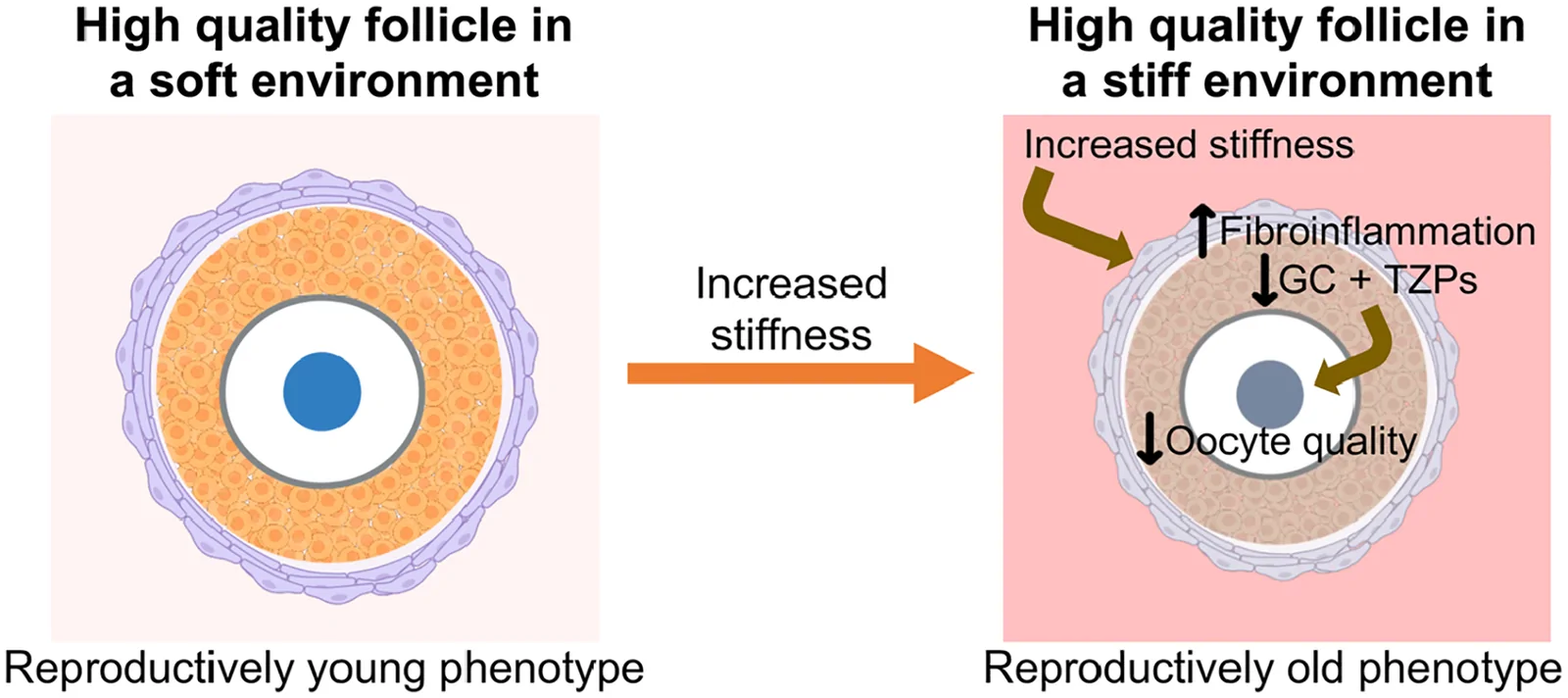
Model. High-quality follicles cultured in a stiff environment acquire a reproductively aged phenotype, characterized by a fibroinflammatory profile, fewer transzonal projections (TZPs), and decreased follicle size and oocyte quality. We hypothesize that granulosa cells (GCs) generate the fibroinflammatory profile. Potential molecular pathways involved in sensing and responding to stiffness might include mechanosensitive pathways such as PI3K/Akt, Hippo, and/or Rho-ROCK. The changes in the mechanical properties of the ovary might be transmitted to the oocyte via TZPs, which mediate communication between granulosa cells and oocytes. Further studies are needed to identify these molecular players.

## Data Availability

All data generated in this study are available upon request from the corresponding author. RNA-seq data is available on Gene Expression Omnibus repository (GSE317288).
