# Supplementary material for: Increased stiffness mimicking ovarian aging induces a fibroinflammatory response in follicles and impairs oocyte quality
Source: Reproduction. Author manuscript; Available in PMC 2026 Oct 25. (PMC13695885; doi:10.1093/reprod/xaaf026)

Supplementary Figure S1

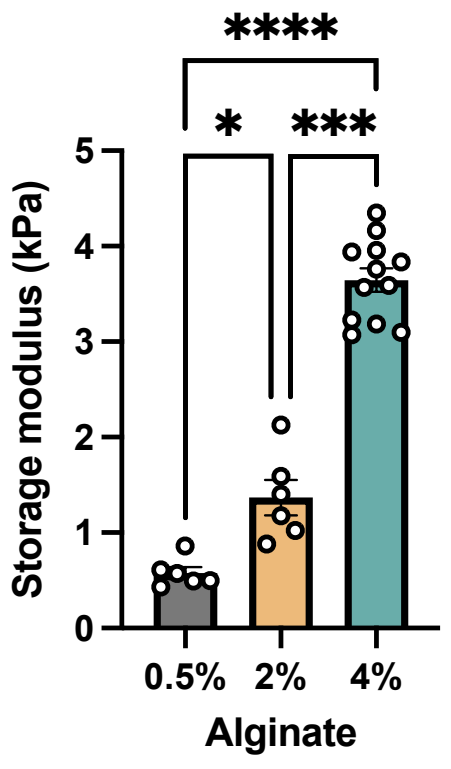

Supplementary Figure S2

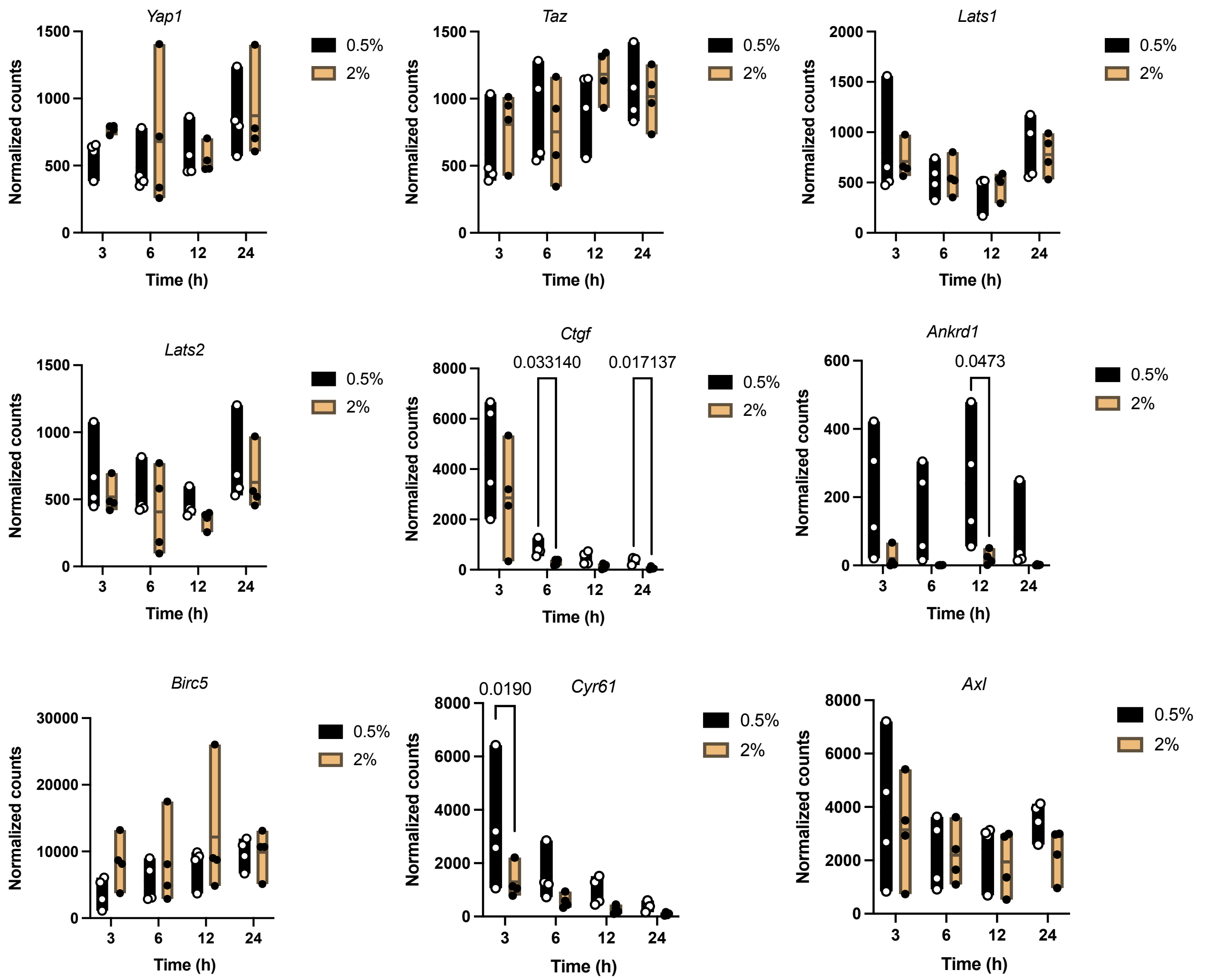

Supplementary Figure S3

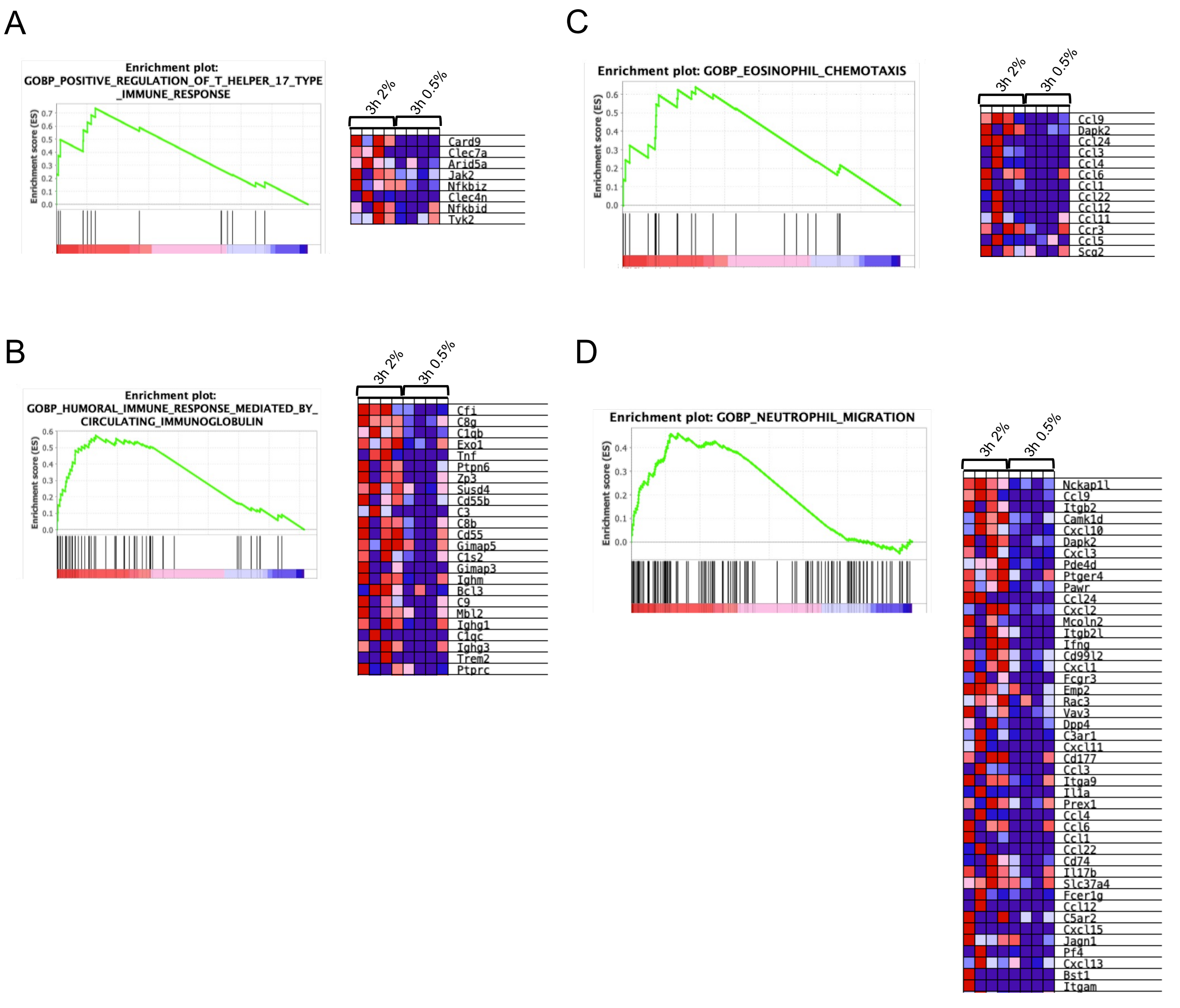

Supplementary Figure S4

A

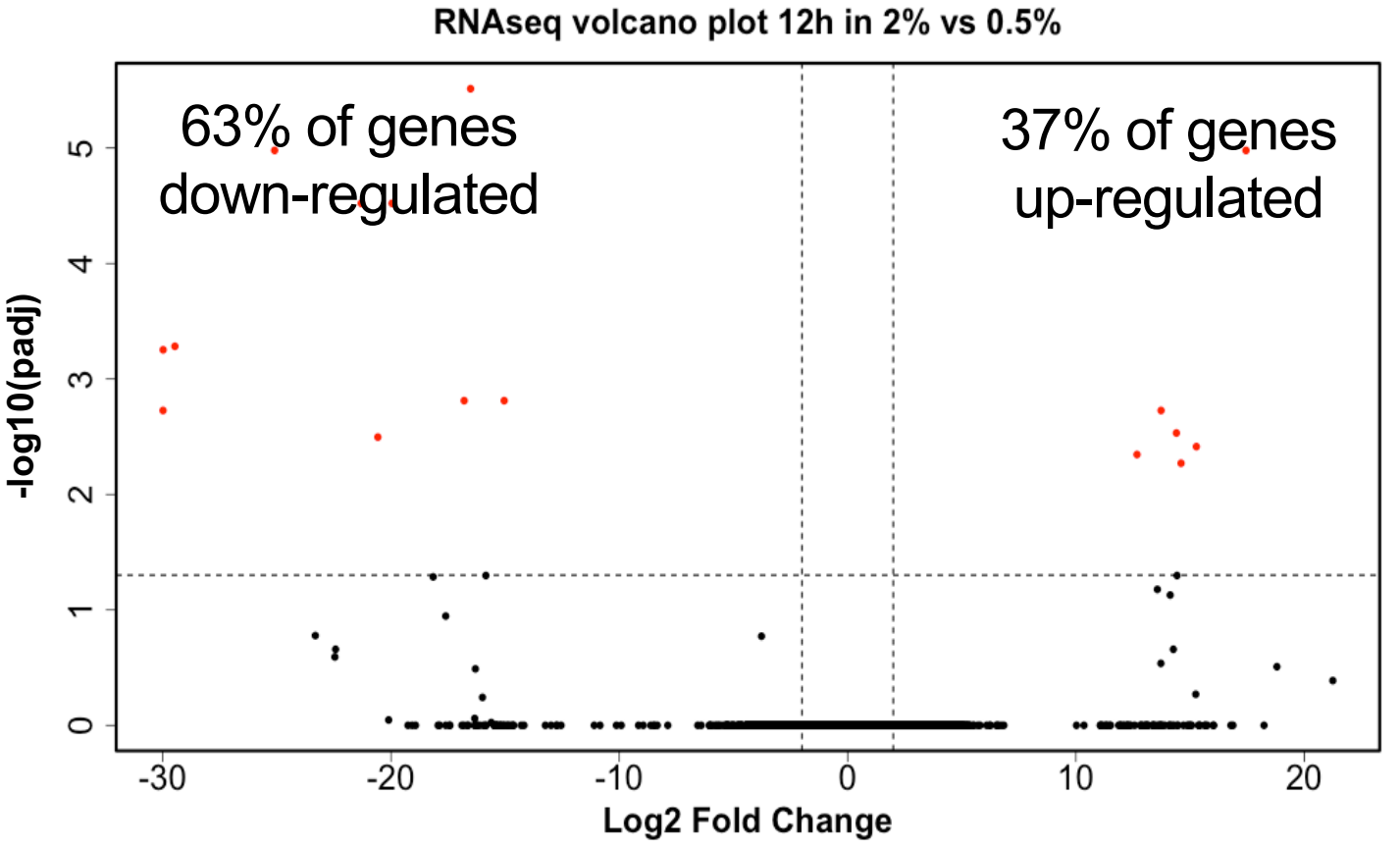

B

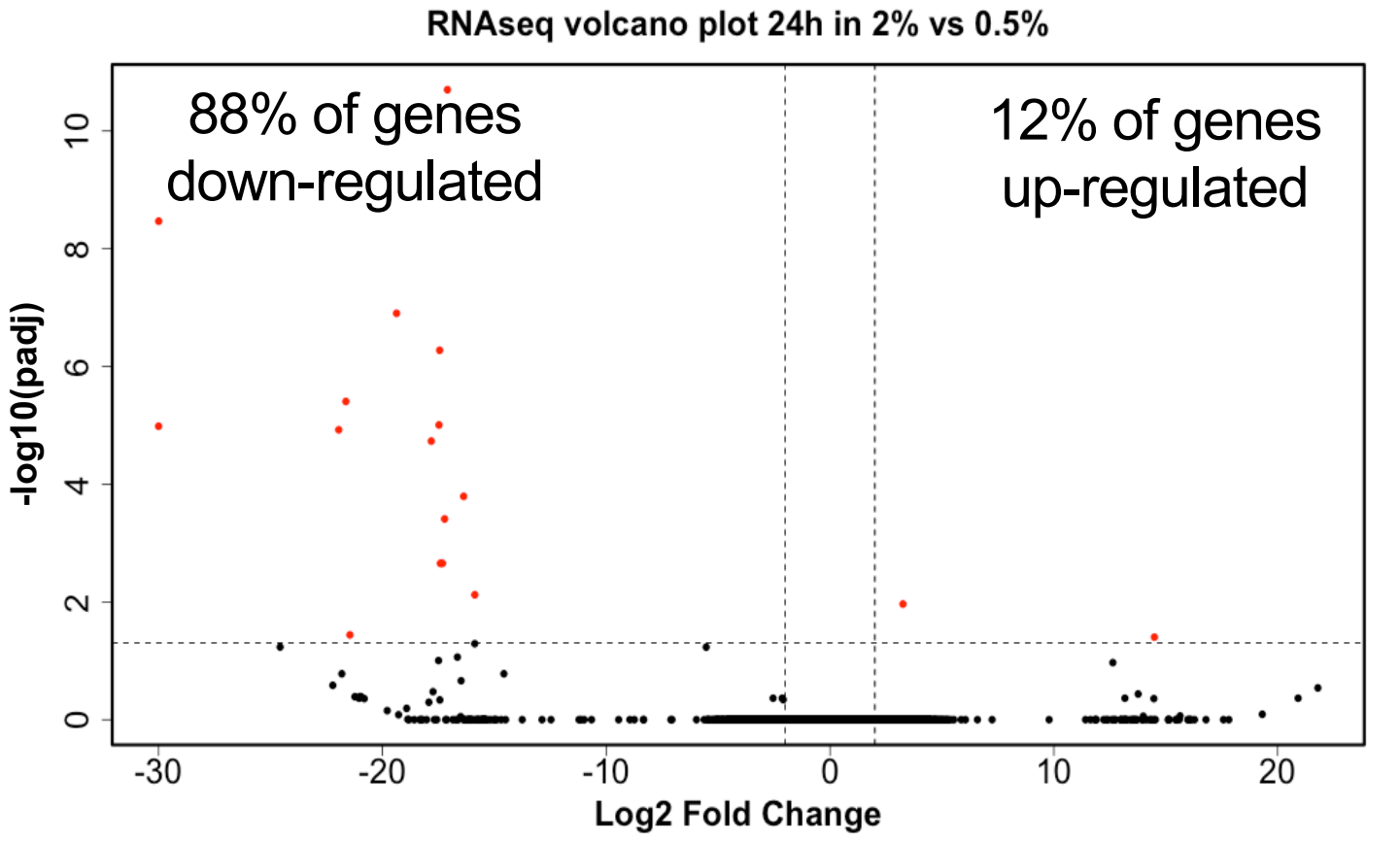

C

Ensembl_ID	Symbol	Gene name	log2FoldChange	padj
ENSMUSG00000030235	*Slco1c1*	Solute Carrier Organic Anion Transporter Family Member 1C1	-25.102	1.048E-05
ENSMUSG00000042812	*Foxf1*	Forkhead Box F1	17.451	1.048E-05
ENSMUSG00000024397	*Aif1*	Allograft Inflammatory Factor 1	-19.956	3.012E-05
ENSMUSG00000043385	*Olfr267*	Olfactory Receptor Family 2 Subfamily K Member 2	-21.308	3.012E-05
ENSMUSG00000096351	*Samd11*	Sterile Alpha Motif Domain Containing 11	-29.470	5.212E-04
ENSMUSG00000036816	*Atoh7*	Atonal BHLH Transcription Factor 7	-29.986	5.589E-04
ENSMUSG00000050134	*Olfr430*	Olfactory Receptor Family 6 Subfamily N Member 2	-16.798	1.541E-03
ENSMUSG00000075104	*Olfr1219*	Olfactory Receptor Family 4 Subfamily C Member 15	-15.044	1.541E-03
ENSMUSG00000038530	*Rgs4*	Regulator Of G Protein Signaling 4	13.731	1.875E-03
ENSMUSG00000024910	*Ctsw*	Cathepsin W	14.403	2.937E-03
ENSMUSG00000021620	*Acot12*	Acyl-CoA Thioesterase 12	12.677	4.513E-03

D

Ensembl_ID	Symbol	Gene name	log2FoldChange	padj
ENSMUSG00000024027	*Glp1r*	Glucagon Like Peptide 1 Receptor	-21.634	3.928E-06
ENSMUSG00000002341	*Ncan*	Neurocan	-16.371	1.609E-04
ENSMUSG00000035283	*Adrb1*	Adrenoceptor Beta 1	-17.317	2.211E-03
ENSMUSG00000043385	*Olfr267*	Olfactory Receptor Family 2 Subfamily K Member 2	-17.409	2.211E-03
ENSMUSG00000045731	*Pnoc*	Prepronociceptin	3.257	1.086E-02
ENSMUSG00000044055	*Otos*	Otospiralin	14.492	3.958E-02

E

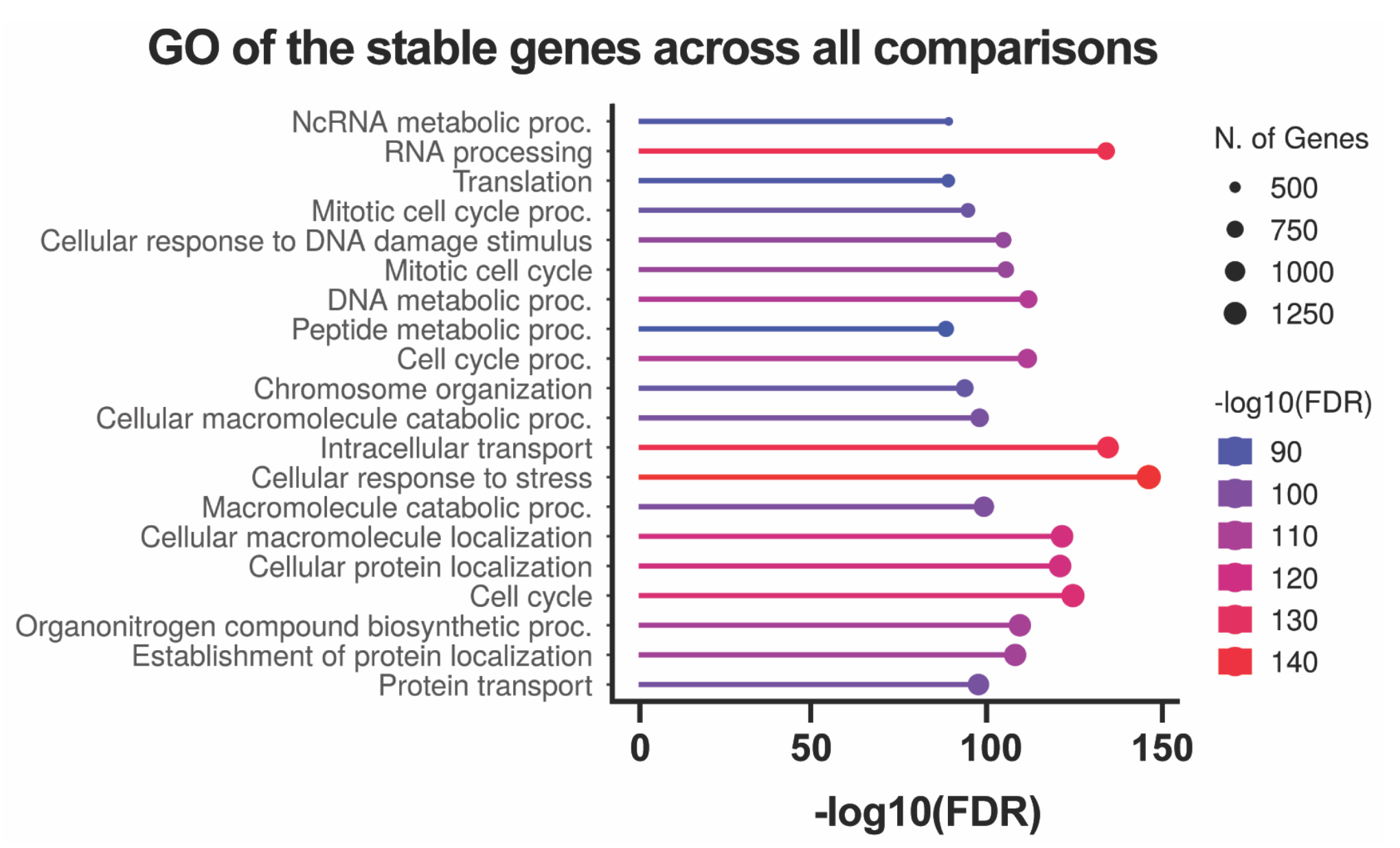

Supplementary Figure S5

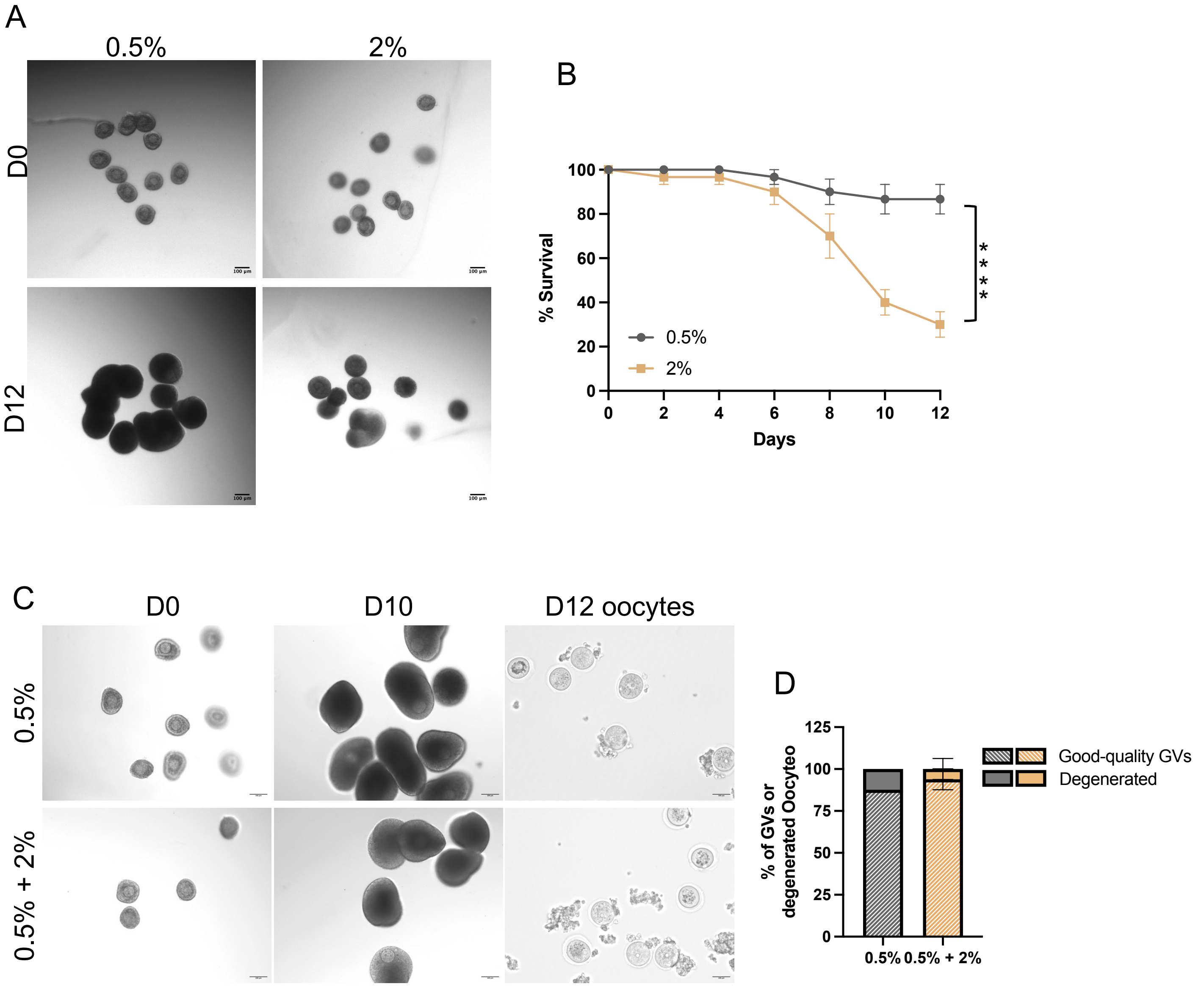

Supplementary Figure S6

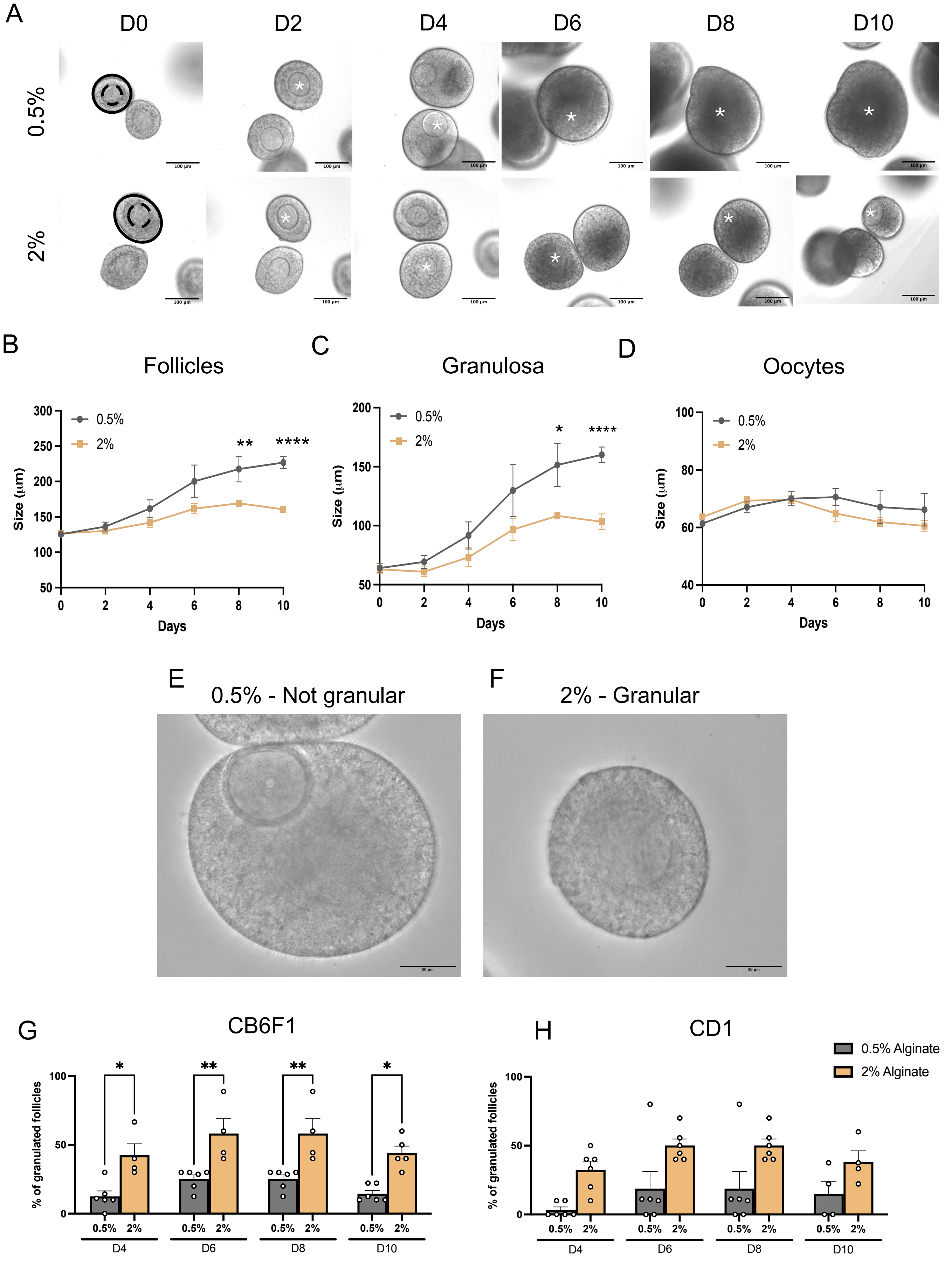

Supplementary Figure S7

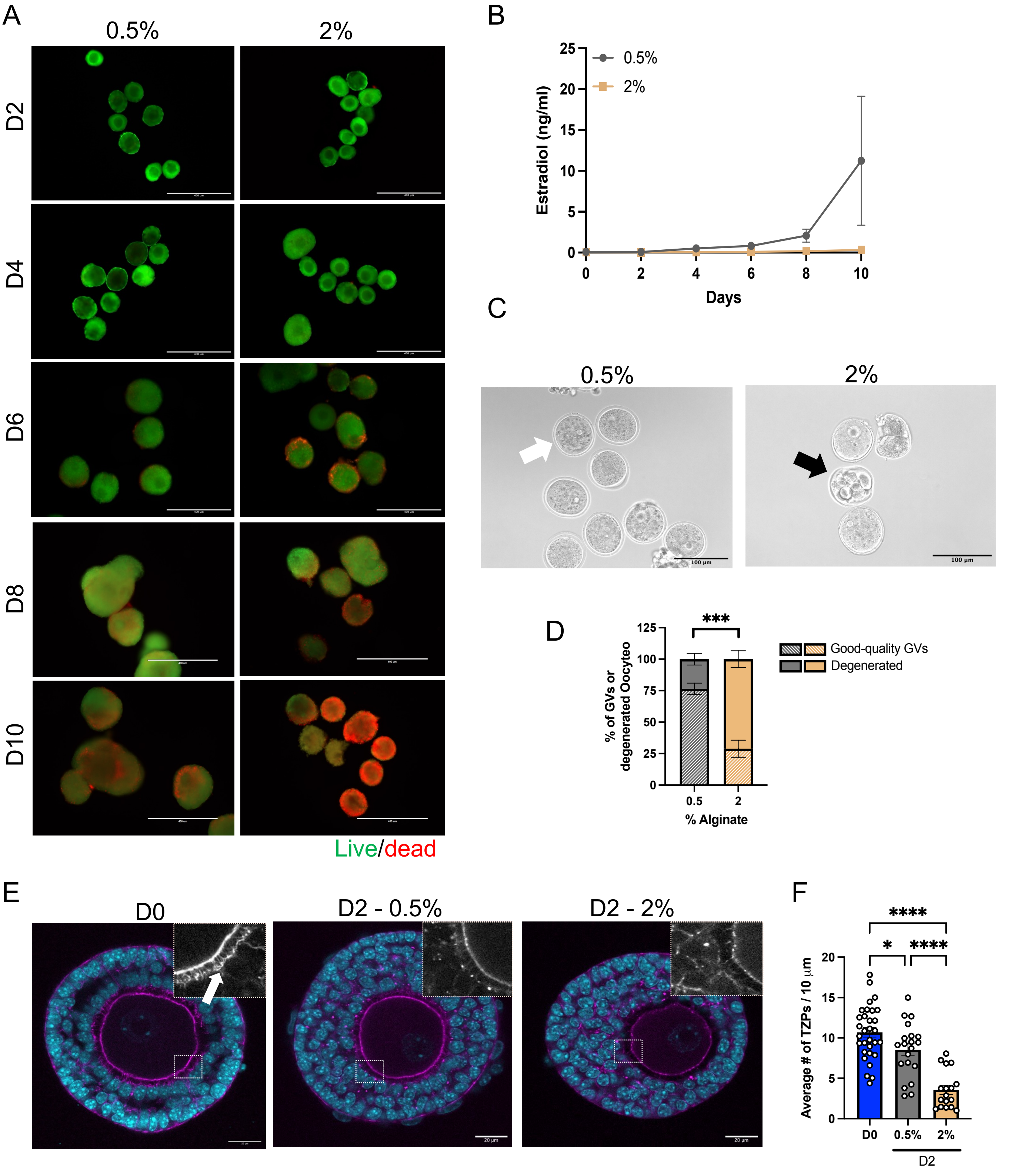

Supplement: Supplementary file 1 [file NIHMS2217685-supplement-Suppl_Figures.pdf]
