# Supplementary material for: Increased stiffness mimicking ovarian aging induces a fibroinflammatory response in follicles and impairs oocyte quality
Source: Reproduction. Author manuscript; Available in PMC 2026 Oct 25. (PMC13695885; doi:10.1093/reprod/xaaf026)
Supplement: Supplementary file 2 [file NIHMS2217685-supplement-Suppl_Tables.pdf]

Supplementary Table S1.

List of analyzed samples

Sample ID	Condition	Replicate
S0_5pct_12h_Replica_1_S5	0_5_12h	1
S0_5pct_12h_Replica_2_S13	0_5_12h	2
S0_5pct_12h_Replica_3_S21	0_5_12h	3
S0_5pct_12h_Replica_4_S29	0_5_12h	4
S0_5pct_24hh_Replica_1_S7	0_5_24h	1
S0_5pct_24hh_Replica_2_S15	0_5_24h	2
S0_5pct_24h_Replica_3_S23	0_5_24h	3
S0_5pct_24h_Replica_4_S31	0_5_24h	4
S0_5pct_3h_Replica_1_S1	0_5_3h	1
S0_5pct_3h_Replica_2_S9	0_5_3h	2
S0_5pct_3h_Replica_3_S17	0_5_3h	3
S0_5pct_3h_Replica_4_S25	0_5_3h	4
S0_5pct_6h_Replica_1_S3	0_5_6h	1
S0_5pct_6h_Replica_2_S11	0_5_6h	2
S0_5pct_6h_Replica_3_S19	0_5_6h	3
S0_5pct_6h_Replica_4_S27	0_5_6h	4
S2pct_12h_Replica_1_S6	2_12h	1
S2pct_12h_Replica_2_S14	2_12h	2
S2pct_12h_Replica_3_S22	2_12h	3
S2pct_12h_Replica_4_S30	2_12h	4
S2pct_24h_Replica_1_S8	2_24h	1
S2pct_24h_Replica_2_S16	2_24h	2
S2pct_24h_Replica_3_S24	2_24h	3
S2pct_24h_Replica_4_S32	2_24h	4
S2pct_3h_Replica_1_S2	2_3h	1
S2pct_3h_Replica_2_S10	2_3h	2
S2pct_3h_Replica_3_S18	2_3h	3
S2pct_3h_Replica_4_S26	2_3h	4
S2pct_6h_Replica_1_S4	2_6h	1
S2pct_6h_Replica_2_S12	2_6h	2
S2pct_6h_Replica_3_S20	2_6h	3
S2pct_6h_Replica_4_S28	2_6h	4

Supplementary Table S2.

List of DEGs between 0.5% and 2% at 3h

Ensembl_ID	SYMBOL	log2FoldChang	pvalue	padj
ENSMUSG00000055069	Rab39	-30.000	5.66E-27	2.46E-22
ENSMUSG00000028037	Ifi44	20.185	1.45E-17	3.15E-13
ENSMUSG00000028841	Cnksr1	-17.824	4.50E-17	6.52E-13
ENSMUSG00000116220	AC147041.1	-22.603	1.73E-13	1.87E-09
ENSMUSG00000074311	Vmn1r139	17.062	2.98E-11	2.59E-07
ENSMUSG00000058976	Usp17lc	16.021	1.89E-10	1.17E-06
ENSMUSG00000069516	Lyz2	21.039	1.81E-10	1.17E-06
ENSMUSG00000040747	Cd53	18.919	6.42E-10	3.48E-06
ENSMUSG00000115509	Gm49012	-29.997	4.90E-09	2.36E-05
ENSMUSG00000115108	Gm49228	-17.883	7.41E-09	3.22E-05
ENSMUSG00000022875	Kng1	15.263	2.64E-08	9.56E-05
ENSMUSG00000091930	Vmn2r52	-29.081	2.62E-08	9.56E-05
ENSMUSG00000053977	Cd8a	-17.971	4.22E-08	1.41E-04
ENSMUSG00000042812	Foxf1	15.652	5.10E-08	1.58E-04
ENSMUSG00000004730	Adgre1	19.307	3.15E-07	8.16E-04
ENSMUSG00000102701	9430083B18	-29.991	3.19E-07	8.16E-04
ENSMUSG00000102912	Gm20731	-29.990	3.18E-07	8.16E-04
ENSMUSG00000087374	Gm15457	16.137	3.80E-07	9.17E-04
ENSMUSG00000085791	Rpl30-ps9	-16.508	4.78E-07	1.09E-03
ENSMUSG00000002341	Ncan	14.540	6.02E-07	1.24E-03
ENSMUSG00000098439	Hm629797	14.691	5.79E-07	1.24E-03
ENSMUSG00000032257	Ankk1	-29.233	8.88E-07	1.73E-03
ENSMUSG00000116804	AC115797.3	-15.562	9.16E-07	1.73E-03
ENSMUSG00000085479	9430073C21	13.811	1.33E-06	2.41E-03
ENSMUSG00000059279	Olfr224	28.011	1.83E-06	2.95E-03
ENSMUSG00000113746	Gm48242	-17.602	1.80E-06	2.95E-03
ENSMUSG00000114649	3110006O06	-17.890	1.70E-06	2.95E-03
ENSMUSG00000068048	Rhox9	14.092	2.04E-06	3.16E-03
ENSMUSG00000043329	Gm8849	14.114	2.16E-06	3.23E-03
ENSMUSG00000073929	Trim30e-ps1	15.671	3.69E-06	5.33E-03
ENSMUSG00000114157	Gm47870	13.255	5.33E-06	7.46E-03
ENSMUSG00000035352	Ccl12	26.223	8.47E-06	1.15E-02
ENSMUSG00000087681	Gm13565	-16.237	1.04E-05	1.36E-02
ENSMUSG00000066688	Gm13083	15.014	1.49E-05	1.90E-02
ENSMUSG00000108891	Olfr1291-ps1	14.162	1.55E-05	1.92E-02
ENSMUSG00000028750	Pla2g2c	12.921	1.65E-05	1.99E-02
ENSMUSG00000103877	Gm21358	12.964	1.78E-05	2.09E-02
ENSMUSG00000110162	Gm45356	-22.147	2.21E-05	2.53E-02
ENSMUSG00000000982	Ccl3	6.486	2.64E-05	2.86E-02
ENSMUSG00000027880	Slc25a54	14.065	2.63E-05	2.86E-02
ENSMUSG00000002781	Tmem143	1.987	2.84E-05	2.93E-02

ENSMUSG00000057903	Olfr739	15.568	2.81E-05	2.93E-02
ENSMUSG00000021768	Dusp13	6.350	3.32E-05	3.35E-02
ENSMUSG00000063779	Chil4	20.156	3.39E-05	3.35E-02
ENSMUSG00000048616	Nog	-6.883	3.67E-05	3.48E-02
ENSMUSG00000057766	Ankrd29	-4.069	3.84E-05	3.48E-02
ENSMUSG00000076939	Iglv3	13.885	3.85E-05	3.48E-02
ENSMUSG00000097903	Gm26679	17.051	3.80E-05	3.48E-02
ENSMUSG00000116894	AC154473.1	15.420	4.16E-05	3.69E-02
ENSMUSG00000114752	Gm35110	-15.234	4.38E-05	3.81E-02
ENSMUSG00000027483	Bpifa1	-23.704	5.70E-05	4.86E-02

Supplementary Table S3.

List of DEGs between 0.5% and 2% at 6h

Ensembl_ID	SYMBOL	og2FoldChang	pvalue	padj
ENSMUSG00000029378	Areg	-20.996	6.85E-24	2.40E-19
ENSMUSG00000078922	Tgtp1	-21.151	2.55E-22	4.47E-18
ENSMUSG00000114422	Gm30411	-30.000	7.15E-22	8.37E-18
ENSMUSG00000030366	Ceacam12	-29.999	2.54E-19	2.23E-15
ENSMUSG00000104698	Gm42602	17.633	1.14E-15	8.02E-12
ENSMUSG00000091923	Gm8267	-29.998	1.71E-15	9.99E-12
ENSMUSG00000114157	Gm47870	-21.558	8.32E-15	4.18E-11
ENSMUSG00000058976	Usp17lc	-19.183	1.60E-14	7.03E-11
ENSMUSG00000102676	Gm37435	-29.102	1.84E-14	7.20E-11
ENSMUSG00000116884	AC127341.5	-29.106	3.63E-14	1.27E-10
ENSMUSG00000035780	Ugt2a3	-28.837	4.38E-14	1.40E-10
ENSMUSG00000109031	Gm45201	-19.679	6.23E-14	1.82E-10
ENSMUSG00000099906	Gm28653	-25.991	3.85E-13	1.04E-09
ENSMUSG00000028750	Pla2g2c	-21.518	1.08E-12	2.70E-09
ENSMUSG00000104821	Gm42481	-30.000	1.39E-12	3.24E-09
ENSMUSG00000079356	Mettl4-ps1	-29.999	2.27E-12	4.98E-09
ENSMUSG00000057710	9630041A04	17.041	2.86E-12	5.91E-09
ENSMUSG00000021620	Acot12	17.441	3.49E-12	6.82E-09
ENSMUSG00000058114	Olfr127	-30.000	3.77E-12	6.97E-09
ENSMUSG00000020395	Itk	-16.480	4.08E-12	7.16E-09
ENSMUSG00000038257	Glra3	15.712	5.63E-12	9.41E-09
ENSMUSG00000089854	Gm16133	17.642	6.13E-12	9.78E-09
ENSMUSG00000002341	Ncan	19.337	8.72E-12	1.33E-08
ENSMUSG00000063394	Olfr293	-29.998	1.30E-11	1.90E-08
ENSMUSG00000075105	Olfr1218	-29.156	2.24E-11	3.02E-08
ENSMUSG00000109299	Gm45164	-17.450	2.16E-11	3.02E-08
ENSMUSG00000088789	Scarna13	-17.769	2.77E-11	3.61E-08
ENSMUSG00000077711	AF357399	-29.194	2.94E-11	3.68E-08
ENSMUSG00000106543	Gm43378	-18.791	3.16E-11	3.83E-08
ENSMUSG00000097444	Gm10489	-19.366	3.37E-11	3.95E-08
ENSMUSG00000104903	Gm43707	-17.295	3.64E-11	4.12E-08
ENSMUSG00000067813	Xkr9	-18.604	4.35E-11	4.63E-08
ENSMUSG00000109446	Gm9195	-17.004	4.32E-11	4.63E-08
ENSMUSG00000075020	Mir670hg	-18.159	5.01E-11	5.17E-08
ENSMUSG00000025804	Ccr1	-23.703	1.32E-10	1.32E-07
ENSMUSG00000042812	Foxf1	17.683	4.15E-10	4.05E-07
ENSMUSG00000030669	Calca	-15.845	7.41E-10	6.85E-07
ENSMUSG00000105820	Gm43400	-15.171	7.38E-10	6.85E-07
ENSMUSG00000115564	Gm49199	-29.997	1.15E-09	1.03E-06
ENSMUSG00000021070	Bdkrb2	16.661	1.49E-09	1.31E-06
ENSMUSG00000068048	Rhox9	-17.346	2.51E-09	2.15E-06
ENSMUSG00000103772	Gm36933	-18.025	3.92E-09	3.27E-06

ENSMUSG00000115330	Gm49221	15.380	4.00E-09	3.27E-06
ENSMUSG00000046717	Igbp1b	-16.686	4.13E-09	3.30E-06
ENSMUSG00000047108	Dnajb7	-18.940	4.83E-09	3.77E-06
ENSMUSG00000103877	Gm21358	-17.633	8.56E-09	6.54E-06
ENSMUSG00000101268	2010310C07	-16.948	9.42E-09	7.04E-06
ENSMUSG00000104406	Gm38014	16.109	1.37E-08	1.00E-05
ENSMUSG00000097934	6720483E21	16.380	1.98E-08	1.42E-05
ENSMUSG00000081357	Xlr4e-ps	-16.438	2.45E-08	1.72E-05
ENSMUSG00000111312	Gm47205	-21.274	3.36E-08	2.31E-05
ENSMUSG00000024803	Ankrd1	-8.461	4.22E-08	2.85E-05
ENSMUSG00000090304	Vmn2r99	14.851	7.63E-08	5.06E-05
ENSMUSG00000066688	Gm13083	-18.310	9.47E-08	6.16E-05
ENSMUSG00000087622	Gm12290	-13.641	9.86E-08	6.30E-05
ENSMUSG00000111065	Gm47676	-16.716	1.25E-07	7.82E-05
ENSMUSG00000110738	Gm47092	-26.797	1.92E-07	1.18E-04
ENSMUSG00000022878	Adipoq	14.611	2.09E-07	1.27E-04
ENSMUSG00000086061	Gm13075	-17.170	2.89E-07	1.72E-04
ENSMUSG00000027857	Tshb	15.772	3.90E-07	2.25E-04
ENSMUSG00000053977	Cd8a	-16.891	3.93E-07	2.25E-04
ENSMUSG00000084899	Gm15344	12.356	3.98E-07	2.25E-04
ENSMUSG00000102713	Gm29994	-16.990	4.31E-07	2.40E-04
ENSMUSG00000027801	Tm4sf4	15.496	4.51E-07	2.47E-04
ENSMUSG00000110363	Gm45576	-29.999	4.57E-07	2.47E-04
ENSMUSG00000115686	Gm49310	-21.460	5.29E-07	2.81E-04
ENSMUSG00000103159	F830112A20	13.784	6.67E-07	3.50E-04
ENSMUSG00000070704	Ugt2b36	15.787	7.99E-07	4.13E-04
ENSMUSG00000070077	Mir491	-13.636	8.23E-07	4.19E-04
ENSMUSG00000113746	Gm48242	-17.910	1.12E-06	5.60E-04
ENSMUSG00000096655	1700065D16	14.934	1.21E-06	5.97E-04
ENSMUSG00000111874	Gm36539	-16.282	1.25E-06	6.11E-04
ENSMUSG00000110702	Gm45767	17.581	1.45E-06	6.99E-04
ENSMUSG00000070501	Ifi214	14.348	1.53E-06	7.25E-04
ENSMUSG00000096690	Olfr1296-ps1	-19.863	1.91E-06	8.93E-04
ENSMUSG00000097023	Mir9-3hg	-15.790	2.49E-06	1.15E-03
ENSMUSG00000033208	S100b	4.577	3.47E-06	1.58E-03
ENSMUSG00000029915	Clec5a	15.702	3.65E-06	1.65E-03
ENSMUSG00000114752	Gm35110	-16.962	3.70E-06	1.65E-03
ENSMUSG00000108228	6430584L05	14.166	4.50E-06	1.97E-03
ENSMUSG00000025431	Crisp1	-16.488	4.66E-06	2.02E-03
ENSMUSG00000110618	Gm39822	14.031	5.10E-06	2.18E-03
ENSMUSG00000114253	Gm47798	-16.716	5.68E-06	2.37E-03
ENSMUSG00000114382	Gm4814	12.367	5.67E-06	2.37E-03
ENSMUSG00000048764	Tmprss11f	13.739	6.00E-06	2.48E-03
ENSMUSG00000030827	Fgf21	-6.230	7.41E-06	3.03E-03
ENSMUSG00000024548	Setbp1	4.930	8.97E-06	3.62E-03
ENSMUSG00000020057	Dram1	5.593	1.18E-05	4.65E-03
ENSMUSG00000083228	Gm11779	16.614	1.17E-05	4.65E-03

ENSMUSG00000004837	Grap	-17.021	1.27E-05	4.90E-03
ENSMUSG00000099891	Gm5575	-3.388	1.27E-05	4.90E-03
ENSMUSG00000056600	Olfr90	-25.676	1.30E-05	4.97E-03
ENSMUSG00000053916	Nanp	-3.515	1.51E-05	5.71E-03
ENSMUSG00000086118	Gm14169	-16.235	1.66E-05	6.18E-03
ENSMUSG00000034839	Larp6	-3.695	2.25E-05	8.33E-03
ENSMUSG00000096773	Olfr675	24.748	2.41E-05	8.81E-03
ENSMUSG00000109548	Gm45066	24.652	2.59E-05	9.39E-03
ENSMUSG00000055159	4930583K01	-15.143	2.63E-05	9.43E-03
ENSMUSG00000047363	Cstad	4.596	2.91E-05	1.03E-02
ENSMUSG00000078921	Tgtp2	-5.910	2.96E-05	1.04E-02
ENSMUSG00000085385	Snhg17	-1.536	3.17E-05	1.10E-02
ENSMUSG00000095248	Olfr681	-24.326	3.44E-05	1.18E-02
ENSMUSG00000022875	Kng1	11.899	3.75E-05	1.28E-02
ENSMUSG00000020154	Ptprb	-4.054	3.80E-05	1.28E-02
ENSMUSG00000104527	Gm37332	19.351	4.41E-05	1.46E-02
ENSMUSG00000107140	Gm43811	-20.954	4.38E-05	1.46E-02
ENSMUSG00000102597	Gm38382	-24.220	4.66E-05	1.53E-02
ENSMUSG00000058400	Qrfpr	-23.782	5.37E-05	1.74E-02
ENSMUSG00000055109	Gm15155	12.532	5.83E-05	1.88E-02
ENSMUSG00000094745	Olfr954	-17.018	6.85E-05	2.19E-02
ENSMUSG00000105701	Gm42587	-23.665	6.93E-05	2.19E-02
ENSMUSG00000085379	2310058D17	-2.771	7.36E-05	2.31E-02
ENSMUSG00000063779	Chil4	-19.337	7.97E-05	2.48E-02
ENSMUSG00000070306	Ccdc153	-15.982	9.16E-05	2.82E-02
ENSMUSG00000047225	Olfr684	-14.433	9.71E-05	2.97E-02
ENSMUSG00000027499	Pkia	-2.477	1.03E-04	3.11E-02
ENSMUSG00000073902	Gm1966	-19.142	1.13E-04	3.36E-02
ENSMUSG00000104937	Gm43057	-17.974	1.13E-04	3.36E-02
ENSMUSG00000105811	Gm42707	-16.594	1.14E-04	3.36E-02
ENSMUSG00000061410	Zcchc14	1.183	1.35E-04	3.94E-02
ENSMUSG00000109147	4930431P19	-1.890	1.36E-04	3.96E-02
ENSMUSG00000109733	Gm33940	-22.621	1.43E-04	4.11E-02
ENSMUSG00000067642	Adgrf3	14.033	1.47E-04	4.18E-02
ENSMUSG00000095903	Olfr968	-18.814	1.49E-04	4.21E-02
ENSMUSG00000112139	Gm47431	-22.543	1.51E-04	4.23E-02
ENSMUSG00000100315	1700031P21	-22.526	1.52E-04	4.25E-02
ENSMUSG00000085763	Smc2os	3.802	1.57E-04	4.34E-02
ENSMUSG00000037390	Muc3	14.296	1.63E-04	4.46E-02
ENSMUSG00000036052	Dnajb5	-3.482	1.66E-04	4.51E-02
ENSMUSG00000075107	Olfr1216	-22.353	1.71E-04	4.62E-02
ENSMUSG00000104938	Gm42840	-18.058	1.73E-04	4.64E-02
ENSMUSG00000062082	Cd200r4	-22.306	1.77E-04	4.66E-02
ENSMUSG00000086675	Plxna4os2	-22.314	1.76E-04	4.66E-02
ENSMUSG00000022129	Dct	-18.275	1.79E-04	4.70E-02
ENSMUSG00000042433	Pih1h3b	21.942	1.81E-04	4.71E-02

Supplementary Table S4.

List of DEGs between 3h-24h in 0.5%

Ensembl_ID	SYMBOL	og2FoldChang	pvalue	padj
ENSMUSG00000069917	Hba-a2	-29.630	6.30E-07	1.20E-04
ENSMUSG00000024027	Glp1r	-21.106	2.44E-10	2.32E-07
ENSMUSG00000073940	Hbb-bt	-20.113	7.21E-04	1.98E-02
ENSMUSG00000042433	Pih1h3b	-17.736	2.74E-03	4.74E-02
ENSMUSG00000096606	Tpbgl	-16.707	1.95E-03	3.77E-02
ENSMUSG00000021680	Crhbp	-8.674	8.27E-07	1.42E-04
ENSMUSG00000022296	Baalc	-8.581	7.84E-08	2.34E-05
ENSMUSG00000020911	Krt19	-8.326	1.83E-04	7.81E-03
ENSMUSG00000115620	Gm45924	-7.793	4.85E-04	1.51E-02
ENSMUSG00000001622	Csn3	-7.643	8.69E-04	2.25E-02
ENSMUSG00000062345	Serpinb2	-7.162	1.10E-05	9.77E-04
ENSMUSG00000031635	Anxa10	-7.141	1.81E-03	3.61E-02
ENSMUSG00000085183	Wincr1	-6.700	2.86E-04	1.06E-02
ENSMUSG00000068697	Myoz1	-6.657	6.76E-04	1.90E-02
ENSMUSG00000030890	Ilk	-6.601	1.88E-03	3.69E-02
ENSMUSG00000028031	Dkk2	-6.525	4.20E-05	2.70E-03
ENSMUSG00000070645	Ren1	-6.464	1.04E-03	2.48E-02
ENSMUSG00000028328	Tmod1	-6.436	1.00E-04	5.07E-03
ENSMUSG00000028172	Tacr3	-6.400	5.47E-05	3.27E-03
ENSMUSG00000050359	Sprr1a	-6.364	9.58E-09	4.86E-06
ENSMUSG00000111467	Gm47775	-6.081	2.50E-04	9.58E-03
ENSMUSG00000032487	Ptgs2	-6.057	1.20E-06	1.88E-04
ENSMUSG00000050541	Adra1b	-6.044	4.76E-05	2.94E-03
ENSMUSG00000007946	Phox2a	-5.896	2.49E-03	4.46E-02
ENSMUSG00000033730	Egr3	-5.789	3.37E-05	2.30E-03
ENSMUSG00000029304	Spp1	-5.643	4.53E-08	1.45E-05
ENSMUSG00000022602	Arc	-5.494	3.32E-05	2.28E-03
ENSMUSG00000021403	Serpinb9b	-5.372	1.13E-07	3.23E-05
ENSMUSG00000047638	Nr1h4	-5.138	1.45E-06	2.17E-04
ENSMUSG00000097084	Foxl1	-5.092	1.10E-03	2.57E-02
ENSMUSG00000046223	Plaur	-4.973	2.34E-12	3.93E-09
ENSMUSG00000033174	Mgll	-4.960	3.39E-05	2.31E-03
ENSMUSG00000022805	Maats1	-4.901	1.36E-05	1.16E-03
ENSMUSG00000032394	Igdcc3	-4.854	2.57E-03	4.54E-02
ENSMUSG00000030854	Ptpn5	-4.793	4.77E-07	9.55E-05
ENSMUSG00000050335	Lgals3	-4.733	3.05E-13	9.52E-10
ENSMUSG00000027068	Dhrs9	-4.654	1.71E-03	3.47E-02
ENSMUSG00000024912	Fosl1	-4.628	1.84E-05	1.47E-03
ENSMUSG00000097746	Gm6225	-4.624	2.87E-03	4.89E-02
ENSMUSG00000047171	Helt	-4.551	1.54E-03	3.21E-02
ENSMUSG00000074199	Krtdap	-4.442	5.39E-05	3.24E-03
ENSMUSG00000010797	Wnt2	-4.404	1.22E-03	2.76E-02

ENSMUSG00000050578	Mmp13	-4.400	1.99E-03	3.84E-02
ENSMUSG00000015652	Steap1	-4.389	2.14E-11	2.74E-08
ENSMUSG00000030827	Fgf21	-4.371	1.20E-04	5.75E-03
ENSMUSG00000037868	Egr2	-4.306	1.07E-04	5.29E-03
ENSMUSG00000003283	Hck	-4.214	2.48E-06	3.24E-04
ENSMUSG00000063531	Sema3e	-4.170	1.71E-03	3.46E-02
ENSMUSG00000031297	Slc7a3	-4.143	1.49E-06	2.20E-04
ENSMUSG00000031383	Dusp9	-4.097	4.34E-04	1.39E-02
ENSMUSG00000007655	Cav1	-4.089	2.34E-11	2.83E-08
ENSMUSG00000023905	Tnfrsf12a	-4.012	8.10E-13	1.61E-09
ENSMUSG00000115009	G930009F23	-3.986	2.33E-03	4.26E-02
ENSMUSG00000091020	Gm5828	-3.964	1.37E-03	2.98E-02
ENSMUSG00000028128	F3	-3.963	1.25E-07	3.42E-05
ENSMUSG00000112134	Gm40723	-3.924	1.20E-03	2.73E-02
ENSMUSG00000005148	Klf5	-3.920	9.95E-05	5.05E-03
ENSMUSG00000066270	Gm10157	-3.906	2.38E-03	4.33E-02
ENSMUSG00000029752	Asns	-3.883	5.62E-12	8.75E-09
ENSMUSG00000055407	Map6	-3.855	5.77E-04	1.70E-02
ENSMUSG00000056031	9330154J02I	-3.850	1.92E-03	3.74E-02
ENSMUSG00000028776	Tinagl1	-3.826	1.91E-10	1.90E-07
ENSMUSG00000026479	Lamc2	-3.822	1.33E-06	2.05E-04
ENSMUSG00000115243	Gm5207	-3.808	4.43E-06	5.17E-04
ENSMUSG00000024640	Psat1	-3.741	2.77E-11	3.14E-08
ENSMUSG00000042734	Ttc9	-3.738	2.08E-03	3.95E-02
ENSMUSG00000039126	Prune2	-3.732	1.76E-09	1.07E-06
ENSMUSG00000028480	Glipr2	-3.722	9.05E-04	2.30E-02
ENSMUSG00000019960	Dusp6	-3.681	4.24E-08	1.40E-05
ENSMUSG00000055301	Adh7	-3.667	9.77E-04	2.39E-02
ENSMUSG00000022686	B3gnt5	-3.635	2.27E-03	4.20E-02
ENSMUSG00000019997	Ctgf	-3.626	4.36E-07	9.14E-05
ENSMUSG00000074743	Thbd	-3.612	2.22E-03	4.13E-02
ENSMUSG00000020256	Aldh1l2	-3.611	6.19E-05	3.56E-03
ENSMUSG00000035799	Twist1	-3.592	1.74E-03	3.51E-02
ENSMUSG00000039462	Col10a1	-3.568	2.77E-03	4.78E-02
ENSMUSG00000073274	Gm14636	-3.568	3.98E-04	1.31E-02
ENSMUSG00000027313	Chac1	-3.549	1.30E-06	2.01E-04
ENSMUSG00000023046	Igfbp6	-3.470	1.72E-14	1.25E-10
ENSMUSG00000029334	Prkg2	-3.469	2.16E-08	8.56E-06
ENSMUSG00000032715	Trib3	-3.469	6.53E-06	6.75E-04
ENSMUSG00000031530	Dusp4	-3.436	1.49E-04	6.78E-03
ENSMUSG00000078249	Hmga1b	-3.402	3.88E-07	8.55E-05
ENSMUSG00000047139	Cd24a	-3.398	8.73E-04	2.25E-02
ENSMUSG00000000058	Cav2	-3.371	6.55E-09	3.48E-06
ENSMUSG00000024990	Rbp4	-3.362	2.14E-04	8.70E-03
ENSMUSG00000031367	Ap1s2	-3.305	3.80E-04	1.29E-02
ENSMUSG00000037946	Fgd3	-3.253	7.31E-04	2.00E-02
ENSMUSG00000051111	Sv2c	-3.197	5.29E-04	1.60E-02

ENSMUSG00000048482	Bdnf	-3.153	1.40E-03	3.00E-02
ENSMUSG00000027611	Procr	-3.138	2.89E-03	4.92E-02
ENSMUSG00000024907	Gal	-3.126	1.29E-04	6.07E-03
ENSMUSG00000028195	Cyr61	-3.082	2.31E-07	5.74E-05
ENSMUSG00000024883	Rin1	-3.062	3.92E-04	1.31E-02
ENSMUSG00000031355	Arhgap6	-3.049	1.69E-03	3.44E-02
ENSMUSG00000032501	Trib1	-3.041	2.30E-03	4.22E-02
ENSMUSG00000024587	Nars	-3.035	1.14E-09	7.33E-07
ENSMUSG00000017144	Rnd3	-3.033	2.19E-06	2.95E-04
ENSMUSG00000021835	Bmp4	-3.032	1.61E-07	4.23E-05
ENSMUSG00000023043	Krt18	-3.018	3.02E-05	2.10E-03
ENSMUSG00000026547	Tagln2	-2.996	2.49E-04	9.55E-03
ENSMUSG00000029161	Cgref1	-2.984	7.55E-06	7.36E-04
ENSMUSG00000021822	Plau	-2.971	3.99E-04	1.31E-02
ENSMUSG00000037855	Zfp365	-2.949	2.70E-05	1.95E-03
ENSMUSG00000055737	Ghr	-2.937	2.26E-08	8.80E-06
ENSMUSG00000050222	Il17d	-2.937	5.58E-06	6.12E-04
ENSMUSG00000022241	Tars	-2.931	2.06E-08	8.49E-06
ENSMUSG00000034271	Jdp2	-2.927	2.15E-05	1.65E-03
ENSMUSG00000046711	Hmga1	-2.907	2.97E-05	2.09E-03
ENSMUSG00000005124	Wisp1	-2.906	2.58E-05	1.88E-03
ENSMUSG00000022180	Slc7a8	-2.901	2.48E-03	4.44E-02
ENSMUSG00000031574	Star	-2.898	1.52E-07	4.10E-05
ENSMUSG00000047821	Trim16	-2.894	2.81E-03	4.82E-02
ENSMUSG00000025140	Pycr1	-2.867	9.24E-04	2.31E-02
ENSMUSG00000028680	Plk3	-2.848	2.49E-05	1.83E-03
ENSMUSG00000028811	Yars	-2.841	2.39E-07	5.80E-05
ENSMUSG00000089812	Gm15867	-2.839	1.29E-03	2.87E-02
ENSMUSG00000038418	Egr1	-2.829	1.31E-03	2.90E-02
ENSMUSG00000026204	Ptprn	-2.818	4.24E-04	1.37E-02
ENSMUSG00000083512	Gm12749	-2.816	2.93E-04	1.08E-02
ENSMUSG00000019970	Sgk1	-2.812	3.57E-05	2.42E-03
ENSMUSG00000101431	Gm7901	-2.798	2.82E-05	2.02E-03
ENSMUSG00000029446	Psph	-2.792	1.43E-14	1.25E-10
ENSMUSG00000062661	Ncs1	-2.776	4.53E-06	5.23E-04
ENSMUSG00000020312	Shc2	-2.746	1.07E-03	2.53E-02
ENSMUSG00000072944	Nup62cl	-2.735	3.66E-05	2.47E-03
ENSMUSG00000073016	Uprt	-2.729	2.75E-03	4.76E-02
ENSMUSG00000106390	Gm5551	-2.724	7.74E-05	4.17E-03
ENSMUSG00000081729	Hspa9-ps1	-2.694	3.61E-04	1.24E-02
ENSMUSG00000052837	Junb	-2.671	2.55E-03	4.53E-02
ENSMUSG00000025403	Shmt2	-2.670	7.08E-09	3.68E-06
ENSMUSG00000031434	Morc4	-2.652	1.02E-04	5.14E-03
ENSMUSG00000086711	Gm15482	-2.645	2.81E-03	4.82E-02
ENSMUSG00000096740	Lbhd1	-2.642	1.23E-03	2.77E-02
ENSMUSG00000006442	Srm	-2.637	7.18E-08	2.18E-05
ENSMUSG00000081819	Gm12722	-2.625	2.36E-03	4.29E-02

ENSMUSG00000010755	Cars	-2.619	9.92E-12	1.44E-08
ENSMUSG00000029777	Gars	-2.618	7.06E-10	4.97E-07
ENSMUSG00000005667	Mthfd2	-2.612	1.16E-07	3.25E-05
ENSMUSG00000026749	Nek6	-2.606	6.52E-07	1.22E-04
ENSMUSG00000037960	Card19	-2.603	3.89E-08	1.35E-05
ENSMUSG00000041324	Inhba	-2.599	8.90E-04	2.28E-02
ENSMUSG00000032802	Srxn1	-2.598	4.48E-05	2.81E-03
ENSMUSG00000020325	Fstl3	-2.583	2.39E-03	4.34E-02
ENSMUSG00000057604	Lmcd1	-2.573	2.93E-04	1.08E-02
ENSMUSG00000034839	Larp6	-2.561	2.44E-03	4.40E-02
ENSMUSG00000003541	Ier3	-2.560	5.09E-06	5.75E-04
ENSMUSG00000037060	Cavin3	-2.554	1.39E-03	2.99E-02
ENSMUSG00000043336	Filip1l	-2.540	5.70E-04	1.69E-02
ENSMUSG00000083465	Gm11652	-2.517	9.80E-06	8.88E-04
ENSMUSG00000003348	Mob3a	-2.515	5.10E-05	3.10E-03
ENSMUSG00000097180	2700038G22	-2.500	2.38E-08	9.12E-06
ENSMUSG00000022557	Bop1	-2.457	1.91E-06	2.70E-04
ENSMUSG00000037465	Klf10	-2.445	1.04E-05	9.32E-04
ENSMUSG00000113769	5033406O09	-2.437	8.99E-05	4.74E-03
ENSMUSG00000030717	Nupr1	-2.433	2.74E-03	4.74E-02
ENSMUSG00000060950	Trmt61a	-2.430	9.29E-05	4.81E-03
ENSMUSG00000097000	Gm17435	-2.405	6.51E-04	1.85E-02
ENSMUSG00000039419	Cntnap2	-2.388	2.49E-06	3.24E-04
ENSMUSG00000040322	Slc25a24	-2.385	3.25E-06	4.03E-04
ENSMUSG00000029096	Htra3	-2.383	1.36E-03	2.96E-02
ENSMUSG00000025007	Aldh18a1	-2.373	6.90E-07	1.27E-04
ENSMUSG00000090394	4930523C07	-2.367	9.28E-05	4.81E-03
ENSMUSG00000030222	Rerg	-2.362	1.04E-04	5.19E-03
ENSMUSG00000038335	Tsr1	-2.345	4.71E-13	1.17E-09
ENSMUSG00000038279	Nop2	-2.334	3.23E-10	2.61E-07
ENSMUSG00000050017	Pitpnb	-2.321	2.20E-05	1.68E-03
ENSMUSG00000102048	Gm6075	-2.315	1.83E-04	7.80E-03
ENSMUSG00000053398	Phgdh	-2.313	5.60E-07	1.09E-04
ENSMUSG00000028069	Gpatch4	-2.305	5.12E-09	2.80E-06
ENSMUSG00000028063	Lmna	-2.295	9.13E-05	4.78E-03
ENSMUSG00000025287	Acot9	-2.273	2.03E-07	5.14E-05
ENSMUSG00000022114	Spry2	-2.266	5.19E-04	1.58E-02
ENSMUSG00000045763	Basp1	-2.247	8.51E-06	8.01E-04
ENSMUSG00000068739	Sars	-2.239	1.02E-06	1.70E-04
ENSMUSG00000104560	Gm8115	-2.237	6.56E-04	1.86E-02
ENSMUSG00000085666	Gm9855	-2.234	3.56E-04	1.22E-02
ENSMUSG00000026784	Pdss1	-2.234	8.56E-10	5.83E-07
ENSMUSG00000000561	Wdr77	-2.224	4.22E-07	9.04E-05
ENSMUSG00000041506	Rrp9	-2.223	2.39E-07	5.80E-05
ENSMUSG00000032051	Fdx1	-2.214	7.57E-05	4.11E-03
ENSMUSG00000051223	Bzw1	-2.203	2.59E-07	6.00E-05
ENSMUSG00000027610	Gss	-2.196	6.57E-07	1.22E-04

ENSMUSG00000024493	Lars	-2.194	2.88E-11	3.14E-08
ENSMUSG00000024037	Wdr4	-2.173	5.63E-06	6.15E-04
ENSMUSG00000028896	Rcc1	-2.162	2.53E-06	3.27E-04
ENSMUSG00000024381	Bin1	-2.161	1.60E-04	7.15E-03
ENSMUSG00000053746	Ptrh1	-2.160	3.95E-05	2.59E-03
ENSMUSG00000070167	Snora57	-2.159	4.00E-04	1.31E-02
ENSMUSG00000013089	Etv5	-2.152	9.96E-05	5.05E-03
ENSMUSG00000032265	Fam46a	-2.137	1.08E-03	2.54E-02
ENSMUSG00000056501	Cebpb	-2.137	5.17E-04	1.58E-02
ENSMUSG00000010067	Rassf1	-2.136	6.87E-05	3.84E-03
ENSMUSG00000020303	Stc2	-2.129	1.78E-05	1.44E-03
ENSMUSG00000010048	Ifrd2	-2.122	4.77E-04	1.49E-02
ENSMUSG00000025511	Tspan4	-2.113	1.44E-06	2.17E-04
ENSMUSG00000032185	Carm1	-2.105	1.11E-03	2.57E-02
ENSMUSG00000042608	Stk40	-2.102	2.97E-04	1.09E-02
ENSMUSG00000031700	Gpt2	-2.091	1.05E-04	5.19E-03
ENSMUSG00000042515	Mum1l1	-2.089	1.11E-06	1.81E-04
ENSMUSG00000025132	Arhgdia	-2.060	1.36E-03	2.96E-02
ENSMUSG00000053560	Ier2	-2.058	7.60E-04	2.04E-02
ENSMUSG00000081302	Gm12020	-2.042	5.05E-04	1.55E-02
ENSMUSG00000025171	Ubtd1	-2.027	5.99E-04	1.75E-02
ENSMUSG00000091898	Tnnc1	-2.008	6.10E-05	3.53E-03
ENSMUSG00000029171	Pgm1	-2.002	8.35E-05	4.45E-03
ENSMUSG00000041360	Pum3	-1.997	4.71E-06	5.35E-04
ENSMUSG00000000916	Nsun5	-1.991	6.65E-06	6.85E-04
ENSMUSG00000029599	Ddx54	-1.989	9.43E-04	2.34E-02
ENSMUSG00000038552	Fndc4	-1.975	2.79E-03	4.79E-02
ENSMUSG00000028010	Gar1	-1.968	1.16E-08	5.76E-06
ENSMUSG00000032966	Fkbp1a	-1.967	2.07E-05	1.60E-03
ENSMUSG00000110126	Gm9347	-1.956	1.72E-03	3.47E-02
ENSMUSG00000063229	Ldha	-1.937	6.90E-05	3.85E-03
ENSMUSG00000038894	Irs2	-1.937	4.07E-04	1.33E-02
ENSMUSG00000024312	Wdr46	-1.932	4.67E-05	2.90E-03
ENSMUSG00000029033	Acap3	-1.927	8.31E-04	2.19E-02
ENSMUSG00000031490	Eif4ebp1	-1.923	2.85E-04	1.06E-02
ENSMUSG00000078862	Gm14326	-1.920	1.38E-03	2.98E-02
ENSMUSG00000023988	Bysl	-1.913	5.44E-06	6.01E-04
ENSMUSG00000015312	Gadd45b	-1.910	2.37E-04	9.19E-03
ENSMUSG00000040354	Mars	-1.909	1.16E-06	1.84E-04
ENSMUSG00000024772	Ehd1	-1.908	3.96E-04	1.31E-02
ENSMUSG00000041774	Ydjc	-1.904	4.45E-04	1.41E-02
ENSMUSG00000002343	Armc6	-1.896	1.45E-03	3.07E-02
ENSMUSG00000003500	Impdh1	-1.893	1.74E-05	1.43E-03
ENSMUSG00000039405	Prss23	-1.879	1.10E-03	2.57E-02
ENSMUSG00000095567	Noc2l	-1.876	3.51E-06	4.31E-04
ENSMUSG00000053801	Grwd1	-1.873	8.40E-07	1.43E-04
ENSMUSG00000011179	Odc1	-1.869	2.89E-07	6.63E-05

ENSMUSG00000060477	Irak2	-1.868	8.88E-04	2.28E-02
ENSMUSG00000026637	Traf5	-1.855	1.17E-03	2.68E-02
ENSMUSG00000027405	Nop56	-1.852	1.49E-08	6.79E-06
ENSMUSG00000048261	Gm4879	-1.848	1.07E-04	5.29E-03
ENSMUSG00000001305	Rrp15	-1.838	3.05E-06	3.85E-04
ENSMUSG00000018932	Map2k3	-1.835	4.47E-04	1.42E-02
ENSMUSG00000025855	Prkar1b	-1.835	2.37E-04	9.19E-03
ENSMUSG00000047565	Acot10	-1.835	1.04E-03	2.48E-02
ENSMUSG00000033706	Smyd5	-1.834	1.95E-05	1.52E-03
ENSMUSG00000025485	Ric8a	-1.828	3.89E-05	2.57E-03
ENSMUSG00000025869	Nop16	-1.821	2.30E-05	1.74E-03
ENSMUSG00000046865	Fbl	-1.816	1.43E-05	1.20E-03
ENSMUSG00000055044	Pdlim1	-1.815	1.91E-03	3.73E-02
ENSMUSG00000003848	Nob1	-1.813	6.79E-05	3.83E-03
ENSMUSG00000020205	Phlda1	-1.808	7.98E-06	7.64E-04
ENSMUSG00000034765	Dusp5	-1.808	1.92E-04	8.06E-03
ENSMUSG00000021595	Nsun2	-1.803	1.35E-05	1.16E-03
ENSMUSG00000074280	Gm6166	-1.801	5.65E-04	1.68E-02
ENSMUSG00000028495	Rps6	-1.790	4.00E-05	2.60E-03
ENSMUSG00000026019	Wdr12	-1.790	2.48E-06	3.24E-04
ENSMUSG00000085385	Snhg17	-1.789	1.18E-06	1.87E-04
ENSMUSG00000038539	Atf5	-1.776	3.44E-04	1.20E-02
ENSMUSG00000037601	Nme1	-1.774	5.45E-06	6.01E-04
ENSMUSG00000042354	Gnl3	-1.772	5.75E-06	6.22E-04
ENSMUSG00000026234	Ncl	-1.772	6.31E-10	4.59E-07
ENSMUSG00000058355	Abce1	-1.768	7.21E-06	7.26E-04
ENSMUSG00000062937	Mtap	-1.767	8.98E-06	8.30E-04
ENSMUSG00000081732	Gm15495	-1.765	5.32E-04	1.61E-02
ENSMUSG00000060647	Gm7099	-1.765	1.22E-03	2.75E-02
ENSMUSG00000042406	Atf4	-1.751	2.38E-05	1.79E-03
ENSMUSG00000001131	Timp1	-1.748	2.06E-04	8.51E-03
ENSMUSG00000059325	Hopx	-1.747	1.27E-03	2.82E-02
ENSMUSG00000022369	Mtbp	-1.732	2.42E-04	9.32E-03
ENSMUSG00000029364	Wsb2	-1.723	8.72E-04	2.25E-02
ENSMUSG00000057541	Pus7	-1.720	1.35E-05	1.16E-03
ENSMUSG00000106664	Gm17936	-1.719	2.23E-04	8.89E-03
ENSMUSG00000031352	Hccs	-1.718	3.74E-06	4.48E-04
ENSMUSG00000057561	Eif1a	-1.715	5.38E-07	1.06E-04
ENSMUSG00000028670	Lypla2	-1.713	6.78E-04	1.90E-02
ENSMUSG00000024190	Dusp1	-1.710	1.35E-03	2.96E-02
ENSMUSG00000083477	Gm5555	-1.706	6.16E-04	1.78E-02
ENSMUSG00000081999	Gm13461	-1.703	6.59E-07	1.22E-04
ENSMUSG00000078789	Dph1	-1.700	9.95E-05	5.05E-03
ENSMUSG00000097769	Snhg4	-1.694	2.54E-04	9.70E-03
ENSMUSG00000021196	Pfkp	-1.693	1.77E-04	7.66E-03
ENSMUSG00000005103	Wdr1	-1.692	4.66E-04	1.46E-02
ENSMUSG00000055612	Cdca7	-1.690	8.72E-07	1.46E-04

ENSMUSG00000070284	Gmppb	-1.686	1.36E-04	6.33E-03
ENSMUSG00000032231	Anxa2	-1.682	9.22E-05	4.80E-03
ENSMUSG00000052688	Rab7b	-1.681	5.67E-04	1.68E-02
ENSMUSG00000024999	Noc3l	-1.679	1.08E-04	5.30E-03
ENSMUSG00000008384	Sertad1	-1.679	1.33E-04	6.22E-03
ENSMUSG00000020547	Bzw2	-1.677	7.32E-04	2.00E-02
ENSMUSG00000048612	Myof	-1.673	8.47E-04	2.22E-02
ENSMUSG00000052151	Plpp2	-1.673	1.01E-05	9.09E-04
ENSMUSG00000051557	Pusl1	-1.668	3.42E-04	1.20E-02
ENSMUSG00000058809	Hspd1-ps3	-1.649	4.23E-05	2.71E-03
ENSMUSG00000043192	Gm1840	-1.641	1.16E-03	2.67E-02
ENSMUSG00000024841	Eif1ad	-1.636	2.53E-07	5.98E-05
ENSMUSG00000040463	Mybbp1a	-1.625	1.73E-08	7.69E-06
ENSMUSG00000030609	Aen	-1.617	1.26E-03	2.81E-02
ENSMUSG00000078812	Eif5a	-1.616	2.90E-04	1.07E-02
ENSMUSG00000048285	Frmd6	-1.616	4.78E-04	1.49E-02
ENSMUSG00000031578	Mak16	-1.614	1.35E-06	2.07E-04
ENSMUSG00000032078	Zpr1	-1.614	1.62E-04	7.22E-03
ENSMUSG00000031960	Aars	-1.611	1.86E-05	1.47E-03
ENSMUSG00000006498	Ptbp1	-1.611	2.63E-03	4.62E-02
ENSMUSG00000063316	Rpl27	-1.609	5.29E-07	1.05E-04
ENSMUSG00000057614	Gnai1	-1.609	5.67E-04	1.68E-02
ENSMUSG00000028729	Ebna1bp2	-1.607	1.20E-07	3.31E-05
ENSMUSG00000025364	Pa2g4	-1.606	1.16E-03	2.66E-02
ENSMUSG00000040675	Mthfd1l	-1.603	1.66E-09	1.03E-06
ENSMUSG00000026020	Nop58	-1.597	3.09E-10	2.59E-07
ENSMUSG00000061024	Rrs1	-1.595	7.41E-07	1.34E-04
ENSMUSG00000112825	Gm9118	-1.594	1.99E-03	3.83E-02
ENSMUSG00000024697	Gna14	-1.591	5.14E-04	1.58E-02
ENSMUSG00000026617	Bpnt1	-1.591	2.04E-08	8.49E-06
ENSMUSG00000019082	Slc25a22	-1.590	5.38E-06	5.99E-04
ENSMUSG00000043424	Eif3j2	-1.588	2.76E-10	2.48E-07
ENSMUSG00000026305	Lrrfip1	-1.588	1.58E-03	3.26E-02
ENSMUSG00000031657	Heatr3	-1.587	1.50E-06	2.20E-04
ENSMUSG00000031568	Rwdd4a	-1.579	6.51E-06	6.75E-04
ENSMUSG00000020116	Pno1	-1.579	4.16E-08	1.40E-05
ENSMUSG00000003355	Fkbp11	-1.574	1.58E-03	3.27E-02
ENSMUSG00000006732	Mettl1	-1.572	2.19E-06	2.95E-04
ENSMUSG00000019849	Prep	-1.571	5.01E-05	3.06E-03
ENSMUSG00000024785	Rcl1	-1.566	7.90E-04	2.11E-02
ENSMUSG00000002825	Qtrt1	-1.564	9.70E-04	2.37E-02
ENSMUSG00000029447	Cct6a	-1.559	2.27E-06	3.04E-04
ENSMUSG00000048007	Timm8a1	-1.557	2.31E-04	9.08E-03
ENSMUSG00000029610	Aimp2	-1.555	3.68E-06	4.46E-04
ENSMUSG00000022148	Fyb	-1.552	2.10E-03	3.98E-02
ENSMUSG00000020075	Ddx21	-1.547	4.26E-10	3.32E-07
ENSMUSG00000037295	Ldlrap1	-1.541	5.39E-04	1.63E-02

ENSMUSG00000024359	Hspa9	-1.540	1.14E-09	7.33E-07
ENSMUSG00000008450	Nutf2	-1.539	7.36E-06	7.30E-04
ENSMUSG00000049957	Ccdc137	-1.539	4.62E-04	1.46E-02
ENSMUSG00000073775	Kti12	-1.538	4.44E-04	1.41E-02
ENSMUSG00000020869	Lrrc59	-1.538	1.25E-04	5.92E-03
ENSMUSG00000026509	Capn2	-1.538	1.69E-03	3.44E-02
ENSMUSG00000079614	Seh1l	-1.533	4.87E-04	1.51E-02
ENSMUSG00000062014	Gmfb	-1.527	3.26E-04	1.17E-02
ENSMUSG00000031792	Usb1	-1.521	1.15E-03	2.65E-02
ENSMUSG00000021288	Klc1	-1.519	3.18E-04	1.15E-02
ENSMUSG00000082121	Gm8199	-1.519	1.57E-03	3.25E-02
ENSMUSG00000066839	Ecsit	-1.516	7.21E-04	1.98E-02
ENSMUSG00000105987	AI506816	-1.516	2.33E-03	4.26E-02
ENSMUSG00000106943	Dancr	-1.514	1.82E-03	3.62E-02
ENSMUSG00000018446	C1qbp	-1.513	3.73E-08	1.34E-05
ENSMUSG00000030079	Ruvbl1	-1.513	7.53E-06	7.36E-04
ENSMUSG00000027804	Ppid	-1.504	2.11E-06	2.92E-04
ENSMUSG00000018433	Nol11	-1.504	3.65E-04	1.24E-02
ENSMUSG00000114419	5430414B19	-1.496	8.93E-04	2.28E-02
ENSMUSG00000056076	Eif3b	-1.487	2.44E-04	9.37E-03
ENSMUSG00000015176	Nolc1	-1.487	2.61E-05	1.90E-03
ENSMUSG00000039910	Cited2	-1.484	2.75E-04	1.03E-02
ENSMUSG00000071359	Tbpl1	-1.480	1.42E-04	6.53E-03
ENSMUSG00000028179	Cth	-1.480	2.29E-03	4.22E-02
ENSMUSG00000026245	Farsb	-1.480	3.00E-04	1.09E-02
ENSMUSG00000005481	Ddx39	-1.476	2.44E-05	1.80E-03
ENSMUSG00000028741	Mrto4	-1.475	1.61E-06	2.33E-04
ENSMUSG00000039682	Lap3	-1.474	5.44E-05	3.26E-03
ENSMUSG00000063888	Rpl7l1	-1.473	1.45E-04	6.63E-03
ENSMUSG00000027533	Fabp5	-1.473	2.71E-03	4.70E-02
ENSMUSG00000029507	Pus1	-1.472	1.14E-04	5.47E-03
ENSMUSG00000001627	Ifrd1	-1.470	1.10E-04	5.33E-03
ENSMUSG00000028318	Polr1e	-1.463	7.61E-05	4.12E-03
ENSMUSG00000014873	Surf2	-1.463	1.34E-03	2.94E-02
ENSMUSG00000021877	Arf4	-1.461	1.28E-03	2.85E-02
ENSMUSG00000024535	Snx24	-1.461	2.85E-03	4.87E-02
ENSMUSG00000027808	Serp1	-1.459	3.10E-06	3.89E-04
ENSMUSG00000029500	Pgam5	-1.453	3.83E-04	1.29E-02
ENSMUSG00000074656	Eif2s2	-1.450	2.14E-06	2.93E-04
ENSMUSG00000021149	Gtpbp4	-1.448	4.78E-09	2.68E-06
ENSMUSG00000026615	Eprs	-1.448	1.63E-07	4.23E-05
ENSMUSG00000000078	Klf6	-1.445	6.40E-04	1.83E-02
ENSMUSG00000024805	Pcgf5	-1.444	2.30E-05	1.74E-03
ENSMUSG00000038510	Rpf2	-1.442	1.66E-04	7.32E-03
ENSMUSG00000031917	Nip7	-1.441	1.61E-07	4.23E-05
ENSMUSG00000030432	Rpl28	-1.429	6.39E-05	3.65E-03
ENSMUSG00000030738	Eif3c	-1.425	2.89E-06	3.66E-04

ENSMUSG00000030268	Bcat1	-1.424	9.37E-04	2.33E-02
ENSMUSG00000026192	Atic	-1.423	2.19E-04	8.81E-03
ENSMUSG00000025980	Hspd1	-1.418	1.55E-12	2.81E-09
ENSMUSG00000028180	Zranb2	-1.411	6.10E-06	6.44E-04
ENSMUSG00000025981	Coq10b	-1.410	1.40E-04	6.47E-03
ENSMUSG00000028683	Eif2b3	-1.406	7.59E-07	1.35E-04
ENSMUSG00000030189	Ybx3	-1.401	8.57E-04	2.23E-02
ENSMUSG00000056131	Pgm3	-1.400	1.38E-03	2.98E-02
ENSMUSG00000026670	Uap1	-1.399	9.33E-06	8.52E-04
ENSMUSG00000032435	Dync1li1	-1.392	1.24E-03	2.79E-02
ENSMUSG00000019814	Ltv1	-1.392	8.11E-05	4.34E-03
ENSMUSG00000064264	Zfp428	-1.389	3.90E-06	4.65E-04
ENSMUSG00000018848	Rars	-1.388	5.39E-05	3.24E-03
ENSMUSG00000024360	Etf1	-1.387	8.72E-04	2.25E-02
ENSMUSG00000040688	Tbl3	-1.387	4.65E-04	1.46E-02
ENSMUSG00000038736	Nudcd1	-1.387	3.03E-05	2.10E-03
ENSMUSG00000028953	Abcf2	-1.386	6.73E-05	3.82E-03
ENSMUSG00000067367	Lyar	-1.384	1.78E-05	1.44E-03
ENSMUSG00000068922	Msto1	-1.384	6.33E-04	1.82E-02
ENSMUSG00000038388	Mpp6	-1.379	1.38E-03	2.98E-02
ENSMUSG00000016554	Eif3d	-1.376	4.60E-07	9.38E-05
ENSMUSG00000021116	Eif2s1	-1.371	1.94E-05	1.52E-03
ENSMUSG00000001785	Pwp1	-1.360	3.80E-05	2.53E-03
ENSMUSG00000039356	Exosc2	-1.360	2.07E-04	8.53E-03
ENSMUSG00000030759	Far1	-1.359	4.57E-07	9.38E-05
ENSMUSG00000020691	Mettl2	-1.358	5.97E-04	1.75E-02
ENSMUSG00000040028	Elavl1	-1.358	1.60E-03	3.29E-02
ENSMUSG00000003868	Ruvbl2	-1.354	4.65E-05	2.89E-03
ENSMUSG00000027944	Hax1	-1.353	1.05E-06	1.74E-04
ENSMUSG00000057236	Rbbp4	-1.352	1.23E-03	2.78E-02
ENSMUSG00000030983	Bccip	-1.351	2.79E-03	4.81E-02
ENSMUSG00000041057	Wdr43	-1.350	1.32E-05	1.15E-03
ENSMUSG00000020780	Srp68	-1.348	3.66E-04	1.24E-02
ENSMUSG00000011114	Tbrg1	-1.345	1.54E-04	6.94E-03
ENSMUSG00000025995	Wdr75	-1.344	4.16E-05	2.69E-03
ENSMUSG00000034343	Ube2f	-1.343	5.38E-08	1.65E-05
ENSMUSG00000025591	Tma16	-1.342	1.91E-06	2.70E-04
ENSMUSG00000029430	Ran	-1.342	7.67E-07	1.35E-04
ENSMUSG00000027940	Tpm3	-1.341	9.58E-04	2.36E-02
ENSMUSG00000068039	Tcp1	-1.338	2.10E-08	8.50E-06
ENSMUSG00000042215	Bag2	-1.336	4.20E-05	2.70E-03
ENSMUSG00000063802	Hspbp1	-1.336	1.05E-03	2.50E-02
ENSMUSG00000001380	Hars	-1.334	8.53E-04	2.23E-02
ENSMUSG00000031432	Prps1	-1.333	6.97E-04	1.93E-02
ENSMUSG00000020358	Hnrnpab	-1.331	1.23E-04	5.86E-03
ENSMUSG00000023110	Prmt5	-1.329	2.56E-04	9.74E-03
ENSMUSG00000036427	Gpi1	-1.326	9.12E-04	2.31E-02

ENSMUSG00000068856	Sf3b4	-1.325	1.05E-03	2.50E-02
ENSMUSG00000025240	Sacm1l	-1.321	7.65E-04	2.05E-02
ENSMUSG00000035530	Eif1	-1.313	3.95E-08	1.35E-05
ENSMUSG00000062070	Pgk1	-1.311	3.98E-04	1.31E-02
ENSMUSG00000034120	Srsf2	-1.310	3.82E-07	8.50E-05
ENSMUSG00000026492	Tfb2m	-1.309	2.32E-03	4.26E-02
ENSMUSG00000052825	Gm9892	-1.309	3.26E-04	1.17E-02
ENSMUSG00000026356	Dars	-1.299	1.30E-04	6.13E-03
ENSMUSG00000029415	Sdad1	-1.298	9.24E-05	4.80E-03
ENSMUSG00000035726	Supt16	-1.295	1.77E-04	7.66E-03
ENSMUSG00000035828	Pim3	-1.293	1.20E-03	2.73E-02
ENSMUSG00000047649	Cd3eap	-1.292	1.88E-05	1.48E-03
ENSMUSG00000030888	Rrp8	-1.292	2.12E-04	8.60E-03
ENSMUSG00000036693	Nop14	-1.290	2.51E-07	5.98E-05
ENSMUSG00000078877	Gm14295	-1.289	3.76E-05	2.52E-03
ENSMUSG00000096403	Gm9825	-1.288	1.07E-03	2.52E-02
ENSMUSG00000037376	Trmt6	-1.287	1.37E-05	1.17E-03
ENSMUSG00000053289	Ddx10	-1.283	1.89E-04	7.99E-03
ENSMUSG00000034667	Xpot	-1.280	7.66E-04	2.05E-02
ENSMUSG00000081603	Gm14681	-1.279	4.75E-05	2.93E-03
ENSMUSG00000006699	Cdc42	-1.279	2.50E-04	9.58E-03
ENSMUSG00000026520	Pycr2	-1.276	2.68E-03	4.69E-02
ENSMUSG00000027624	Epb41l1	-1.276	4.29E-04	1.38E-02
ENSMUSG00000030057	Cnbp	-1.274	4.28E-05	2.71E-03
ENSMUSG00000041488	Stx3	-1.271	2.98E-05	2.09E-03
ENSMUSG00000063785	Utp14a	-1.269	2.54E-03	4.51E-02
ENSMUSG00000040472	Rabggta	-1.268	1.92E-04	8.06E-03
ENSMUSG00000053907	Mat2a	-1.263	6.29E-14	3.43E-10
ENSMUSG00000067150	Xpo5	-1.259	9.15E-04	2.31E-02
ENSMUSG00000020089	Ppa1	-1.258	1.10E-07	3.19E-05
ENSMUSG00000032557	Uba5	-1.257	5.73E-05	3.39E-03
ENSMUSG00000031278	Acsl4	-1.257	6.83E-05	3.83E-03
ENSMUSG00000031485	Plpbp	-1.256	7.61E-04	2.04E-02
ENSMUSG00000019487	Trip10	-1.255	1.02E-03	2.45E-02
ENSMUSG00000027236	Eif3j1	-1.255	6.25E-07	1.20E-04
ENSMUSG00000024732	Ccdc86	-1.254	1.83E-04	7.81E-03
ENSMUSG00000000339	Rtca	-1.252	7.39E-04	2.01E-02
ENSMUSG00000031167	Rbm3	-1.250	1.10E-04	5.33E-03
ENSMUSG00000016319	Slc25a5	-1.248	2.51E-05	1.84E-03
ENSMUSG00000020925	Ccdc43	-1.247	7.81E-05	4.19E-03
ENSMUSG00000026377	Nifk	-1.238	8.35E-06	7.88E-04
ENSMUSG00000022234	Cct5	-1.226	1.54E-05	1.28E-03
ENSMUSG00000022184	Fbxo4	-1.224	4.14E-04	1.35E-02
ENSMUSG00000046027	Stard5	-1.223	4.40E-04	1.41E-02
ENSMUSG00000021282	Eif5	-1.223	1.41E-05	1.19E-03
ENSMUSG00000054000	Tusc1	-1.222	2.45E-03	4.41E-02
ENSMUSG00000036285	Noa1	-1.221	1.31E-04	6.13E-03

ENSMUSG00000040550	Otud6b	-1.217	1.17E-03	2.69E-02
ENSMUSG00000015363	Trabd	-1.216	2.18E-03	4.07E-02
ENSMUSG00000038612	Mcl1	-1.210	4.00E-04	1.31E-02
ENSMUSG00000028277	Ube2j1	-1.210	2.02E-03	3.86E-02
ENSMUSG00000017999	Ddx27	-1.206	7.22E-06	7.26E-04
ENSMUSG00000010095	Slc3a2	-1.204	1.53E-05	1.28E-03
ENSMUSG00000039114	Nrn1	-1.203	2.15E-03	4.03E-02
ENSMUSG00000022744	Cldnd1	-1.197	3.81E-05	2.53E-03
ENSMUSG00000001707	Eef1e1	-1.197	2.34E-05	1.77E-03
ENSMUSG00000022407	Adsl	-1.195	5.17E-04	1.58E-02
ENSMUSG00000002881	Nab1	-1.193	7.18E-05	3.96E-03
ENSMUSG00000027854	Sike1	-1.190	1.61E-03	3.31E-02
ENSMUSG00000026806	Ddx31	-1.190	3.29E-04	1.17E-02
ENSMUSG00000004127	Trmt10a	-1.190	5.86E-04	1.72E-02
ENSMUSG00000025264	Tsr2	-1.190	1.74E-04	7.58E-03
ENSMUSG00000020250	Txnrd1	-1.189	2.80E-03	4.81E-02
ENSMUSG00000063694	Cycs	-1.189	6.59E-04	1.86E-02
ENSMUSG00000032042	Srpr	-1.188	1.68E-03	3.43E-02
ENSMUSG00000028330	Ncbp1	-1.187	3.41E-04	1.20E-02
ENSMUSG00000029036	Atad3a	-1.182	8.55E-04	2.23E-02
ENSMUSG00000020534	Shmt1	-1.182	2.57E-03	4.54E-02
ENSMUSG00000039841	Zfp800	-1.182	2.71E-04	1.02E-02
ENSMUSG00000002984	Tomm40	-1.179	1.01E-03	2.44E-02
ENSMUSG00000041747	Utp15	-1.176	3.70E-06	4.46E-04
ENSMUSG00000020922	Lsm12	-1.176	1.13E-06	1.83E-04
ENSMUSG00000034274	Thoc5	-1.175	2.61E-03	4.60E-02
ENSMUSG00000024501	Dpysl3	-1.174	3.60E-04	1.24E-02
ENSMUSG00000018697	Aatf	-1.172	5.23E-05	3.17E-03
ENSMUSG00000031948	Kars	-1.171	1.35E-03	2.96E-02
ENSMUSG00000061477	Rps7	-1.170	1.21E-03	2.74E-02
ENSMUSG00000027531	Impa1	-1.169	9.05E-04	2.30E-02
ENSMUSG00000039033	Tasp1	-1.169	1.32E-03	2.92E-02
ENSMUSG00000017801	Mlx	-1.167	2.44E-05	1.80E-03
ENSMUSG00000030327	Necap1	-1.167	4.06E-04	1.33E-02
ENSMUSG00000025613	Cct8	-1.166	7.96E-07	1.38E-04
ENSMUSG00000024436	Mrps18b	-1.165	5.96E-05	3.49E-03
ENSMUSG00000017561	Crlf3	-1.164	2.11E-04	8.60E-03
ENSMUSG00000020430	Pes1	-1.162	1.05E-04	5.21E-03
ENSMUSG00000047714	Ppp1r2	-1.160	2.47E-03	4.43E-02
ENSMUSG00000001774	Chordc1	-1.160	3.01E-08	1.10E-05
ENSMUSG00000058126	Tpm3-rs7	-1.160	1.87E-03	3.68E-02
ENSMUSG00000111877	Gm6477	-1.158	1.65E-04	7.27E-03
ENSMUSG00000030505	Prmt3	-1.155	2.19E-03	4.09E-02
ENSMUSG00000031146	Plp2	-1.155	2.86E-03	4.88E-02
ENSMUSG00000050697	Prkaa1	-1.151	1.69E-04	7.43E-03
ENSMUSG00000021692	Dimt1	-1.150	2.05E-04	8.49E-03
ENSMUSG00000037563	Rps16	-1.146	4.07E-04	1.33E-02

ENSMUSG00000042508	Dmtf1	-1.146	2.29E-04	9.04E-03
ENSMUSG00000022538	Lsg1	-1.146	2.64E-04	9.99E-03
ENSMUSG00000002458	Rgs19	-1.144	1.70E-03	3.45E-02
ENSMUSG00000042660	Wdr55	-1.142	2.20E-03	4.10E-02
ENSMUSG00000006599	Gtf2h1	-1.142	8.27E-04	2.18E-02
ENSMUSG00000004264	Phb2	-1.141	9.06E-04	2.30E-02
ENSMUSG00000036902	Neto2	-1.140	1.84E-04	7.81E-03
ENSMUSG00000022663	Atg3	-1.138	6.45E-05	3.67E-03
ENSMUSG00000025134	Alyref	-1.138	2.76E-05	1.98E-03
ENSMUSG00000030007	Cct7	-1.138	3.53E-04	1.22E-02
ENSMUSG00000028982	Slc25a33	-1.137	1.45E-03	3.06E-02
ENSMUSG00000042225	Ammecr1	-1.134	2.13E-05	1.64E-03
ENSMUSG00000034681	Rnps1	-1.132	4.90E-04	1.52E-02
ENSMUSG00000027076	Timm10	-1.131	1.42E-03	3.01E-02
ENSMUSG00000057113	Npm1	-1.124	7.15E-06	7.26E-04
ENSMUSG00000033554	Dph5	-1.123	3.05E-04	1.11E-02
ENSMUSG00000028990	Lzic	-1.117	3.25E-04	1.17E-02
ENSMUSG00000035960	Apex1	-1.115	2.52E-03	4.49E-02
ENSMUSG00000014226	Cacybp	-1.114	1.84E-03	3.64E-02
ENSMUSG00000063524	Eno1	-1.113	2.29E-03	4.22E-02
ENSMUSG00000033285	Wdr3	-1.112	6.23E-05	3.58E-03
ENSMUSG00000059796	Eif4a1	-1.111	6.37E-05	3.65E-03
ENSMUSG00000038046	Mrm3	-1.110	3.44E-04	1.20E-02
ENSMUSG00000032279	Idh3a	-1.110	1.55E-06	2.26E-04
ENSMUSG00000024966	Stip1	-1.110	5.70E-04	1.69E-02
ENSMUSG00000025190	Got1	-1.105	2.41E-05	1.80E-03
ENSMUSG00000079435	Rpl36a	-1.102	2.03E-03	3.88E-02
ENSMUSG00000030662	Ipo5	-1.097	2.16E-06	2.94E-04
ENSMUSG00000061079	Zfp143	-1.097	1.10E-03	2.57E-02
ENSMUSG00000003070	Efna2	-1.097	2.57E-03	4.54E-02
ENSMUSG00000003808	Farsa	-1.095	6.19E-04	1.79E-02
ENSMUSG00000039660	Spout1	-1.095	2.24E-03	4.16E-02
ENSMUSG00000021958	Pinx1	-1.094	1.57E-04	7.04E-03
ENSMUSG00000032714	Syde1	-1.091	2.91E-03	4.94E-02
ENSMUSG00000027671	Actl6a	-1.090	6.11E-05	3.53E-03
ENSMUSG00000007029	Vars	-1.085	9.53E-04	2.36E-02
ENSMUSG00000020484	Xbp1	-1.082	1.42E-03	3.01E-02
ENSMUSG00000070319	Eif3g	-1.081	1.41E-06	2.14E-04
ENSMUSG00000022752	Tomm70a	-1.080	3.95E-04	1.31E-02
ENSMUSG00000042369	Rbm45	-1.077	1.68E-04	7.38E-03
ENSMUSG00000024528	Srfbp1	-1.077	4.62E-04	1.46E-02
ENSMUSG00000031403	Dkc1	-1.073	8.40E-05	4.46E-03
ENSMUSG00000021578	Ccdc127	-1.071	5.89E-06	6.30E-04
ENSMUSG00000007739	Cct4	-1.068	1.24E-04	5.89E-03
ENSMUSG00000015748	Prpf3	-1.066	2.70E-03	4.70E-02
ENSMUSG00000028252	Ccnc	-1.063	1.05E-03	2.49E-02
ENSMUSG00000025630	Hprt	-1.063	1.25E-03	2.81E-02

ENSMUSG00000033781	Asb13	-1.063	5.51E-04	1.65E-02
ENSMUSG00000034203	Chchd4	-1.061	1.53E-05	1.28E-03
ENSMUSG00000056962	Jmjd6	-1.060	1.91E-03	3.73E-02
ENSMUSG00000027429	Sec23b	-1.057	1.46E-04	6.67E-03
ENSMUSG00000001783	Rtcb	-1.051	3.50E-04	1.21E-02
ENSMUSG00000030045	Mrpl19	-1.050	1.24E-03	2.79E-02
ENSMUSG00000030521	Mphosph10	-1.050	1.39E-03	2.99E-02
ENSMUSG00000028224	Nbn	-1.047	1.09E-03	2.56E-02
ENSMUSG00000022336	Eif3e	-1.046	2.13E-03	4.01E-02
ENSMUSG00000038482	Tfdp1	-1.038	2.84E-05	2.02E-03
ENSMUSG00000006998	Psmd2	-1.037	1.96E-03	3.80E-02
ENSMUSG00000071172	Srsf3	-1.035	1.69E-03	3.45E-02
ENSMUSG00000097195	Snhg5	-1.034	2.73E-03	4.72E-02
ENSMUSG00000030357	Fkbp4	-1.034	2.40E-03	4.36E-02
ENSMUSG00000027122	Arl14ep	-1.033	1.39E-03	2.99E-02
ENSMUSG00000030942	Thumpd1	-1.028	7.75E-05	4.17E-03
ENSMUSG00000021621	Zcchc9	-1.028	1.54E-03	3.21E-02
ENSMUSG00000047945	Marcksl1	-1.025	2.68E-03	4.69E-02
ENSMUSG00000023883	Phf10	-1.021	5.61E-04	1.67E-02
ENSMUSG00000028790	Khdrbs1	-1.019	1.11E-03	2.58E-02
ENSMUSG00000063884	Ptcd3	-1.016	9.93E-05	5.05E-03
ENSMUSG00000034424	Gcsh	-1.015	1.25E-03	2.80E-02
ENSMUSG00000064181	Rab3ip	-1.014	2.44E-03	4.40E-02
ENSMUSG00000001674	Ddx18	-1.012	1.25E-04	5.92E-03
ENSMUSG00000019782	Rwdd1	-1.011	1.91E-04	8.06E-03
ENSMUSG00000030264	Thumpd3	-1.009	1.79E-04	7.70E-03
ENSMUSG00000028869	Gnl2	-1.007	5.71E-04	1.69E-02
ENSMUSG00000035575	Utp6	-1.006	5.94E-04	1.74E-02
ENSMUSG00000022814	Umps	-1.006	3.15E-05	2.17E-03
ENSMUSG00000025393	Atp5b	-1.003	3.46E-04	1.21E-02
ENSMUSG00000008373	Prpf31	-1.000	5.59E-04	1.67E-02
ENSMUSG00000013701	Timm23	-0.999	7.54E-05	4.11E-03
ENSMUSG00000030881	Arfip2	-0.998	6.41E-04	1.83E-02
ENSMUSG00000032026	Rexo2	-0.995	1.52E-03	3.19E-02
ENSMUSG00000040124	Gorab	-0.990	1.21E-03	2.74E-02
ENSMUSG00000041733	Coq5	-0.988	2.76E-03	4.77E-02
ENSMUSG00000022800	Fyttd1	-0.988	1.80E-03	3.60E-02
ENSMUSG00000030224	Strap	-0.987	5.51E-04	1.65E-02
ENSMUSG00000026159	Agfg1	-0.985	5.87E-05	3.45E-03
ENSMUSG00000042719	Naa25	-0.984	2.20E-04	8.83E-03
ENSMUSG00000021018	Polr2h	-0.981	7.64E-06	7.41E-04
ENSMUSG00000021737	Psmd6	-0.980	2.95E-07	6.72E-05
ENSMUSG00000023147	Wrb	-0.979	6.75E-05	3.83E-03
ENSMUSG00000028333	Anp32b	-0.974	1.82E-05	1.46E-03
ENSMUSG00000018326	Ywhab	-0.973	3.39E-04	1.20E-02
ENSMUSG00000017781	Pitpna	-0.973	1.85E-03	3.66E-02
ENSMUSG00000002319	Ipo4	-0.969	2.70E-03	4.70E-02

ENSMUSG00000001891	Ugp2	-0.969	3.28E-04	1.17E-02
ENSMUSG00000022466	Rpap3	-0.965	7.03E-04	1.94E-02
ENSMUSG00000022035	Ccdc25	-0.962	1.42E-04	6.53E-03
ENSMUSG00000038827	Fam206a	-0.962	4.12E-06	4.83E-04
ENSMUSG00000067722	BC003965	-0.960	1.72E-03	3.47E-02
ENSMUSG00000030061	Uba3	-0.959	1.02E-04	5.14E-03
ENSMUSG00000020088	Sar1a	-0.958	6.92E-04	1.92E-02
ENSMUSG00000028034	Fubp1	-0.956	9.58E-04	2.36E-02
ENSMUSG00000030603	Psmc4	-0.956	1.64E-04	7.25E-03
ENSMUSG00000032058	Ppp2r1b	-0.955	5.22E-04	1.59E-02
ENSMUSG00000049751	Rpl36al	-0.937	1.70E-03	3.46E-02
ENSMUSG00000049517	Rps23	-0.936	3.32E-04	1.18E-02
ENSMUSG00000078652	Psme3	-0.936	9.63E-05	4.97E-03
ENSMUSG00000040738	Ints8	-0.935	2.05E-04	8.51E-03
ENSMUSG00000033931	Rbm34	-0.935	4.23E-04	1.37E-02
ENSMUSG00000028719	Cmpk1	-0.935	2.72E-03	4.72E-02
ENSMUSG00000062203	Gspt1	-0.933	4.10E-04	1.34E-02
ENSMUSG00000004788	Eif2b2	-0.930	2.35E-03	4.29E-02
ENSMUSG00000032041	Tirap	-0.924	2.85E-03	4.88E-02
ENSMUSG00000033953	Ppp3r1	-0.919	2.33E-04	9.14E-03
ENSMUSG00000026240	Cops7b	-0.918	2.41E-03	4.38E-02
ENSMUSG00000027162	Lin7c	-0.917	9.40E-04	2.34E-02
ENSMUSG00000038845	Phb	-0.917	1.32E-03	2.92E-02
ENSMUSG00000024081	Cebpz	-0.914	6.29E-04	1.81E-02
ENSMUSG00000020224	Llph	-0.911	1.04E-03	2.48E-02
ENSMUSG00000027357	Crls1	-0.910	4.84E-05	2.97E-03
ENSMUSG00000020720	Psmd12	-0.907	3.47E-04	1.21E-02
ENSMUSG00000030298	Sec13	-0.904	9.44E-04	2.34E-02
ENSMUSG00000032458	Copb2	-0.899	2.90E-03	4.93E-02
ENSMUSG00000004268	Emg1	-0.895	9.16E-05	4.78E-03
ENSMUSG00000025439	Clns1a	-0.895	1.21E-03	2.74E-02
ENSMUSG00000043998	Mgat2	-0.893	9.03E-05	4.75E-03
ENSMUSG00000001525	Tubb5	-0.892	8.45E-04	2.22E-02
ENSMUSG00000044763	Trmt10c	-0.889	1.04E-03	2.48E-02
ENSMUSG00000028322	Exosc3	-0.885	1.77E-04	7.66E-03
ENSMUSG00000021276	Cinp	-0.884	1.36E-03	2.96E-02
ENSMUSG00000049755	Zfp672	-0.882	2.52E-03	4.49E-02
ENSMUSG00000031575	Ash2l	-0.882	7.61E-04	2.04E-02
ENSMUSG00000021810	Ecd	-0.881	3.92E-04	1.31E-02
ENSMUSG00000018965	Ywhah	-0.881	2.92E-03	4.95E-02
ENSMUSG00000021660	Btf3	-0.879	7.07E-04	1.95E-02
ENSMUSG00000014504	Srp19	-0.875	2.93E-03	4.96E-02
ENSMUSG00000024878	Cbwd1	-0.871	3.73E-05	2.51E-03
ENSMUSG00000027170	Eif3m	-0.870	3.49E-04	1.21E-02
ENSMUSG00000061613	U2af1	-0.868	2.02E-03	3.87E-02
ENSMUSG00000024712	Rfk	-0.866	6.36E-04	1.82E-02
ENSMUSG00000055531	Cpsf6	-0.865	1.26E-03	2.81E-02

ENSMUSG00000061207	Stk19	-0.863	1.36E-03	2.96E-02
ENSMUSG00000038822	Hace1	-0.863	2.62E-03	4.61E-02
ENSMUSG00000028187	Rpf1	-0.862	8.24E-04	2.18E-02
ENSMUSG00000020349	Ppp2ca	-0.862	9.20E-04	2.31E-02
ENSMUSG00000024231	Cul2	-0.861	1.67E-03	3.42E-02
ENSMUSG00000027823	Gmps	-0.860	5.99E-04	1.75E-02
ENSMUSG00000036398	Ppp1r11	-0.860	1.88E-03	3.68E-02
ENSMUSG00000028907	Utp11	-0.859	2.36E-04	9.19E-03
ENSMUSG00000038274	Fau	-0.858	1.82E-04	7.80E-03
ENSMUSG00000034321	Exosc1	-0.857	1.13E-03	2.61E-02
ENSMUSG00000029326	Enoph1	-0.856	2.97E-03	5.00E-02
ENSMUSG00000021546	Hnrnpk	-0.848	2.12E-03	4.01E-02
ENSMUSG00000006057	Atp5g1	-0.840	9.08E-04	2.30E-02
ENSMUSG00000020248	Nfyb	-0.837	3.39E-04	1.20E-02
ENSMUSG00000001416	Cct3	-0.836	7.45E-05	4.07E-03
ENSMUSG00000026036	Nif3l1	-0.834	1.10E-03	2.57E-02
ENSMUSG00000022052	Ppp2r2a	-0.831	2.13E-03	4.01E-02
ENSMUSG00000030880	Polr3e	-0.822	6.86E-04	1.91E-02
ENSMUSG00000027905	Ddx20	-0.819	1.13E-04	5.45E-03
ENSMUSG00000021868	Ppif	-0.817	1.70E-03	3.45E-02
ENSMUSG00000022285	Ywhaz	-0.817	1.04E-04	5.19E-03
ENSMUSG00000007338	Mrpl49	-0.817	4.62E-04	1.46E-02
ENSMUSG00000036371	Serbp1	-0.816	1.56E-04	7.03E-03
ENSMUSG00000047879	Usp14	-0.816	9.00E-04	2.30E-02
ENSMUSG00000015290	Ubl4a	-0.814	1.51E-04	6.84E-03
ENSMUSG00000026869	Psmd5	-0.812	6.84E-04	1.91E-02
ENSMUSG00000035021	Baz1a	-0.811	4.45E-04	1.41E-02
ENSMUSG00000028889	Yrdc	-0.810	2.95E-03	4.98E-02
ENSMUSG00000030879	Mrpl17	-0.808	2.69E-04	1.01E-02
ENSMUSG00000061315	Naca	-0.804	1.33E-03	2.94E-02
ENSMUSG00000027601	Mtfr1	-0.803	3.03E-04	1.10E-02
ENSMUSG00000061950	Ppp4r1	-0.799	2.73E-03	4.72E-02
ENSMUSG00000034024	Cct2	-0.798	2.55E-07	5.98E-05
ENSMUSG00000019795	Pcmt1	-0.797	2.63E-05	1.91E-03
ENSMUSG00000040618	Pck2	-0.796	1.47E-03	3.08E-02
ENSMUSG00000027981	Rnpc3	-0.794	2.19E-04	8.81E-03
ENSMUSG00000027714	Exosc9	-0.793	1.17E-03	2.69E-02
ENSMUSG00000055762	Eef1d	-0.791	2.28E-04	9.04E-03
ENSMUSG00000074797	Itpa	-0.790	9.81E-04	2.39E-02
ENSMUSG00000020775	Mrpl38	-0.789	1.73E-04	7.54E-03
ENSMUSG00000036323	Srp72	-0.788	9.39E-04	2.34E-02
ENSMUSG00000033813	Tcea1	-0.785	1.78E-03	3.57E-02
ENSMUSG00000028902	Sf3a3	-0.780	1.40E-04	6.47E-03
ENSMUSG00000028988	Ctnnbip1	-0.775	1.31E-03	2.90E-02
ENSMUSG00000014195	Dnajc7	-0.773	7.44E-04	2.02E-02
ENSMUSG00000032563	Mrpl3	-0.771	4.29E-05	2.71E-03
ENSMUSG00000025580	Eif4a3	-0.753	1.93E-03	3.76E-02

ENSMUSG00000031422	Morf4l2	-0.741	2.78E-04	1.03E-02
ENSMUSG00000031256	Cstf2	-0.734	9.86E-05	5.04E-03
ENSMUSG00000029551	Psmg3	-0.732	8.46E-04	2.22E-02
ENSMUSG00000070697	Utp3	-0.732	1.44E-03	3.04E-02
ENSMUSG00000038446	Cdc40	-0.732	2.13E-03	4.01E-02
ENSMUSG00000020708	Psmc5	-0.727	1.38E-03	2.98E-02
ENSMUSG00000021131	Erh	-0.723	2.88E-03	4.89E-02
ENSMUSG00000038762	Abcf1	-0.720	1.56E-03	3.24E-02
ENSMUSG00000074781	Ube2n	-0.718	1.81E-03	3.61E-02
ENSMUSG00000018565	Elp5	-0.707	5.99E-05	3.49E-03
ENSMUSG00000016018	Skiv2l2	-0.706	1.29E-03	2.86E-02
ENSMUSG00000025917	Cops5	-0.700	5.28E-04	1.60E-02
ENSMUSG00000020180	Snrpd3	-0.696	1.14E-03	2.64E-02
ENSMUSG00000063480	Snu13	-0.694	2.19E-04	8.81E-03
ENSMUSG00000042229	Rabif	-0.692	2.46E-03	4.42E-02
ENSMUSG00000024580	Grpel2	-0.691	3.15E-04	1.14E-02
ENSMUSG00000030754	Copb1	-0.678	9.24E-04	2.31E-02
ENSMUSG00000022774	Ncbp2	-0.671	1.92E-03	3.74E-02
ENSMUSG00000026229	Psmd1	-0.653	1.82E-03	3.62E-02
ENSMUSG00000032002	Dcun1d5	-0.634	9.07E-04	2.30E-02
ENSMUSG00000039640	Mrpl12	-0.634	6.18E-04	1.79E-02
ENSMUSG00000030471	Zdhhc13	-0.627	2.65E-03	4.66E-02
ENSMUSG00000046434	Hnrnpa1	-0.619	2.57E-03	4.54E-02
ENSMUSG00000015120	Ube2i	-0.618	1.55E-03	3.22E-02
ENSMUSG00000029038	Ssu72	-0.616	1.01E-03	2.45E-02
ENSMUSG00000005683	Cs	-0.600	2.86E-03	4.89E-02
ENSMUSG00000024668	Sdhaf2	-0.595	2.96E-04	1.09E-02
ENSMUSG00000044221	Grsf1	-0.579	2.48E-03	4.44E-02
ENSMUSG00000020952	Scfd1	-0.572	1.44E-03	3.04E-02
ENSMUSG00000022204	Ngdn	-0.526	2.19E-03	4.08E-02
ENSMUSG00000004364	Cul3	-0.501	1.81E-03	3.61E-02
ENSMUSG00000027104	Atf2	0.579	1.42E-03	3.01E-02
ENSMUSG00000009030	Pdcl	0.601	2.16E-03	4.05E-02
ENSMUSG00000024248	Cox7a2l	0.699	2.21E-04	8.83E-03
ENSMUSG00000030750	Nsmce1	0.712	6.00E-05	3.49E-03
ENSMUSG00000027304	Rtf1	0.722	1.16E-03	2.67E-02
ENSMUSG00000032398	Snapc5	0.725	6.80E-04	1.91E-02
ENSMUSG00000078570	1110065P20	0.741	2.36E-03	4.30E-02
ENSMUSG00000020738	Sumo2	0.747	2.36E-03	4.29E-02
ENSMUSG00000019942	Cdk1	0.750	1.37E-03	2.98E-02
ENSMUSG00000025102	3110040N11	0.751	7.78E-05	4.18E-03
ENSMUSG00000066724	Gm10175	0.757	2.05E-03	3.90E-02
ENSMUSG00000021109	Hif1a	0.772	1.40E-03	3.00E-02
ENSMUSG00000031360	Ctps2	0.785	1.60E-05	1.33E-03
ENSMUSG00000032599	Ip6k2	0.797	2.00E-03	3.84E-02
ENSMUSG00000019370	Calm3	0.808	1.21E-03	2.74E-02
ENSMUSG00000026568	Mpc2	0.813	5.91E-04	1.74E-02

ENSMUSG00000050732	Vamp8	0.834	1.50E-03	3.14E-02
ENSMUSG00000039159	Ube2h	0.845	1.28E-03	2.85E-02
ENSMUSG00000050552	Lamtor4	0.865	1.94E-03	3.76E-02
ENSMUSG00000028066	Pmf1	0.867	2.13E-05	1.64E-03
ENSMUSG00000031023	Akip1	0.868	6.11E-06	6.44E-04
ENSMUSG00000017286	Glod4	0.888	4.36E-04	1.39E-02
ENSMUSG00000028062	Lamtor2	0.890	9.07E-04	2.30E-02
ENSMUSG00000031534	Smim19	0.890	2.65E-04	9.99E-03
ENSMUSG00000036639	Nudt1	0.905	3.37E-04	1.19E-02
ENSMUSG00000033545	Znrf1	0.907	1.28E-03	2.86E-02
ENSMUSG00000031641	Cbr4	0.914	7.54E-04	2.04E-02
ENSMUSG00000034744	Nagk	0.929	2.18E-03	4.07E-02
ENSMUSG00000040767	Snrnp25	0.942	2.87E-03	4.89E-02
ENSMUSG00000030613	Ccdc90b	0.943	1.10E-04	5.33E-03
ENSMUSG00000022092	Ppp3cc	0.955	9.31E-04	2.32E-02
ENSMUSG00000056394	Lig1	0.972	1.46E-03	3.07E-02
ENSMUSG00000016940	Kctd2	0.979	2.66E-03	4.66E-02
ENSMUSG00000052915	Msl1	0.986	1.51E-03	3.17E-02
ENSMUSG00000078566	Bnip3	0.987	1.37E-03	2.98E-02
ENSMUSG00000045103	Dmd	0.989	1.76E-03	3.53E-02
ENSMUSG00000030301	Ccdc91	0.993	2.70E-03	4.70E-02
ENSMUSG00000053929	Cyhr1	1.009	1.93E-06	2.71E-04
ENSMUSG00000049881	2810025M15	1.016	4.00E-05	2.60E-03
ENSMUSG00000060147	Serpinb6a	1.017	1.76E-03	3.54E-02
ENSMUSG00000040771	Oard1	1.031	1.33E-04	6.20E-03
ENSMUSG00000043411	Usp48	1.033	8.72E-04	2.25E-02
ENSMUSG00000006021	Kptn	1.034	1.11E-03	2.58E-02
ENSMUSG00000030982	9030624J02I	1.035	2.77E-03	4.78E-02
ENSMUSG00000042364	Snx18	1.038	2.23E-04	8.89E-03
ENSMUSG00000022817	Itgb5	1.045	2.43E-03	4.39E-02
ENSMUSG00000005233	Spc25	1.046	5.99E-04	1.75E-02
ENSMUSG00000027160	Ccdc34	1.059	7.56E-05	4.11E-03
ENSMUSG00000040841	Six5	1.072	2.84E-04	1.06E-02
ENSMUSG00000003778	Brd8	1.075	3.88E-04	1.30E-02
ENSMUSG00000061887	Ssbp3	1.078	2.95E-03	4.98E-02
ENSMUSG00000033111	3830406C13	1.080	1.35E-05	1.16E-03
ENSMUSG00000029059	Fam213b	1.084	1.19E-03	2.72E-02
ENSMUSG00000035901	Dennd5a	1.090	3.79E-04	1.28E-02
ENSMUSG00000044201	Cdc25c	1.102	5.02E-04	1.55E-02
ENSMUSG00000042293	Gm5617	1.111	1.57E-03	3.26E-02
ENSMUSG00000049225	Pdp1	1.119	7.24E-06	7.26E-04
ENSMUSG00000028969	Cdk5	1.121	2.14E-03	4.03E-02
ENSMUSG00000032038	St3gal4	1.133	3.86E-04	1.30E-02
ENSMUSG00000035637	Grhpr	1.134	1.82E-04	7.80E-03
ENSMUSG00000030213	Atf7ip	1.136	2.83E-05	2.02E-03
ENSMUSG00000019689	Fmc1	1.137	9.68E-04	2.37E-02
ENSMUSG00000044252	Osbpl1a	1.146	2.21E-04	8.83E-03

ENSMUSG00000021033	Gstz1	1.173	7.41E-06	7.31E-04
ENSMUSG00000027459	Fam110a	1.173	2.36E-03	4.29E-02
ENSMUSG00000040549	Ckap5	1.175	2.99E-04	1.09E-02
ENSMUSG00000022051	Bnip3l	1.176	4.01E-06	4.73E-04
ENSMUSG00000078695	Cisd3	1.179	1.63E-03	3.35E-02
ENSMUSG00000052139	Babam2	1.180	2.08E-04	8.54E-03
ENSMUSG00000060538	Tmem219	1.180	1.65E-03	3.38E-02
ENSMUSG00000001403	Ube2c	1.180	6.11E-05	3.53E-03
ENSMUSG00000002580	Mien1	1.187	1.19E-05	1.04E-03
ENSMUSG00000006717	Acot13	1.187	8.59E-06	8.04E-04
ENSMUSG00000028832	Stmn1	1.188	8.61E-04	2.24E-02
ENSMUSG00000039795	Zfand1	1.192	1.59E-04	7.12E-03
ENSMUSG00000073481	44987	1.195	5.12E-06	5.75E-04
ENSMUSG00000022314	Rad21	1.195	6.22E-04	1.80E-02
ENSMUSG00000035992	Fnip1	1.200	1.92E-03	3.74E-02
ENSMUSG00000072772	Grcc10	1.204	1.63E-04	7.25E-03
ENSMUSG00000030990	Pgap2	1.206	9.15E-05	4.78E-03
ENSMUSG00000029177	Cenpa	1.209	3.43E-06	4.22E-04
ENSMUSG00000033713	Foxn3	1.210	1.86E-03	3.66E-02
ENSMUSG00000028070	Naxe	1.220	8.46E-07	1.43E-04
ENSMUSG00000019842	Traf3ip2	1.226	1.83E-03	3.63E-02
ENSMUSG00000073633	Fbxo36	1.229	1.61E-03	3.32E-02
ENSMUSG00000002804	Nudt14	1.230	9.18E-04	2.31E-02
ENSMUSG00000021215	Net1	1.231	2.03E-03	3.88E-02
ENSMUSG00000037499	Nenf	1.234	1.91E-03	3.73E-02
ENSMUSG00000024253	Dync2li1	1.237	5.94E-05	3.48E-03
ENSMUSG00000033685	Ucp2	1.257	3.49E-04	1.21E-02
ENSMUSG00000057103	Nat8f1	1.258	1.43E-03	3.04E-02
ENSMUSG00000023094	Msrb2	1.274	7.48E-04	2.03E-02
ENSMUSG00000025268	Maged2	1.278	1.53E-03	3.20E-02
ENSMUSG00000034449	Dhrs11	1.280	2.43E-03	4.40E-02
ENSMUSG00000024392	Bag6	1.283	3.84E-04	1.30E-02
ENSMUSG00000042712	Tceal9	1.285	2.00E-03	3.84E-02
ENSMUSG00000022323	Rida	1.287	1.98E-03	3.83E-02
ENSMUSG00000035048	Anapc13	1.296	1.09E-03	2.56E-02
ENSMUSG00000006715	Gmnn	1.296	6.03E-04	1.75E-02
ENSMUSG00000027165	B230118H07	1.302	1.10E-04	5.33E-03
ENSMUSG00000023055	Calcoco1	1.317	8.90E-04	2.28E-02
ENSMUSG00000033429	Mcee	1.317	4.25E-05	2.71E-03
ENSMUSG00000034354	Mtmr3	1.325	6.51E-04	1.85E-02
ENSMUSG00000023966	Rsph9	1.326	2.82E-06	3.59E-04
ENSMUSG00000027332	Ivd	1.326	1.10E-04	5.33E-03
ENSMUSG00000048922	Cdca2	1.332	4.05E-04	1.33E-02
ENSMUSG00000031644	Nek1	1.336	2.96E-03	4.99E-02
ENSMUSG00000036644	Tbc1d9b	1.340	2.61E-04	9.90E-03
ENSMUSG00000002058	Unc119	1.342	9.83E-05	5.04E-03
ENSMUSG00000046341	Gm11223	1.345	8.22E-04	2.18E-02

ENSMUSG00000097059	Fam120aos	1.350	2.29E-03	4.22E-02
ENSMUSG00000044080	S100a1	1.350	9.62E-04	2.36E-02
ENSMUSG00000026495	Efcab2	1.359	2.10E-03	3.98E-02
ENSMUSG00000030727	Rabep2	1.360	1.04E-03	2.48E-02
ENSMUSG00000045211	Nudt18	1.361	1.81E-04	7.79E-03
ENSMUSG00000044475	Ascc1	1.362	1.34E-04	6.23E-03
ENSMUSG00000027454	Gins1	1.372	2.81E-03	4.82E-02
ENSMUSG00000051817	Sox12	1.372	1.90E-04	8.01E-03
ENSMUSG00000022661	Cd200	1.373	1.98E-03	3.82E-02
ENSMUSG00000036782	Klhl13	1.373	1.74E-03	3.51E-02
ENSMUSG00000004951	Hspb1	1.374	1.24E-03	2.79E-02
ENSMUSG00000031099	Smarca1	1.376	9.29E-04	2.32E-02
ENSMUSG00000036764	Dnajc12	1.376	7.46E-04	2.02E-02
ENSMUSG00000020914	Top2a	1.380	1.26E-03	2.81E-02
ENSMUSG00000022982	Sod1	1.388	1.03E-03	2.47E-02
ENSMUSG00000025466	Fuom	1.394	4.76E-04	1.49E-02
ENSMUSG00000061461	Smim20	1.394	3.86E-04	1.30E-02
ENSMUSG00000028470	Hint2	1.397	3.87E-05	2.56E-03
ENSMUSG00000074749	Kiz	1.401	1.81E-05	1.46E-03
ENSMUSG00000062866	Phactr2	1.402	3.63E-04	1.24E-02
ENSMUSG00000035142	Nubpl	1.402	6.93E-05	3.86E-03
ENSMUSG00000034032	Rpap1	1.406	7.08E-04	1.95E-02
ENSMUSG00000000876	Pxmp4	1.431	5.50E-04	1.65E-02
ENSMUSG00000062031	Pgghg	1.432	2.34E-03	4.28E-02
ENSMUSG00000037628	Cdkn3	1.434	3.97E-04	1.31E-02
ENSMUSG00000040128	Pnrc1	1.434	1.50E-06	2.20E-04
ENSMUSG00000028795	Ccdc28b	1.436	1.82E-08	7.95E-06
ENSMUSG00000038286	Bphl	1.441	4.13E-05	2.68E-03
ENSMUSG00000001666	Ddt	1.446	8.78E-04	2.26E-02
ENSMUSG00000098090	2700099C18	1.447	1.67E-03	3.41E-02
ENSMUSG00000029022	Miip	1.451	4.45E-04	1.41E-02
ENSMUSG00000028549	Itgb3bp	1.453	8.86E-05	4.68E-03
ENSMUSG00000041308	Sntb2	1.453	3.09E-04	1.12E-02
ENSMUSG00000045312	Lhfpl2	1.458	1.18E-03	2.70E-02
ENSMUSG00000032175	Tyk2	1.470	2.11E-04	8.60E-03
ENSMUSG00000038718	Pbx3	1.483	3.25E-05	2.23E-03
ENSMUSG00000033577	Myo6	1.485	9.98E-04	2.42E-02
ENSMUSG00000026211	Obsl1	1.491	3.94E-05	2.59E-03
ENSMUSG00000051343	Rab11fip5	1.495	2.93E-03	4.96E-02
ENSMUSG00000097415	AU020206	1.497	5.04E-04	1.55E-02
ENSMUSG00000022550	Adck5	1.500	2.26E-04	9.00E-03
ENSMUSG00000066456	Hmgn3	1.515	4.05E-05	2.63E-03
ENSMUSG00000032295	Man2c1	1.517	1.10E-03	2.57E-02
ENSMUSG00000002043	Trappc6a	1.521	3.80E-05	2.53E-03
ENSMUSG00000043644	0610009L18	1.530	2.27E-03	4.20E-02
ENSMUSG00000045257	Morn2	1.531	3.42E-04	1.20E-02
ENSMUSG00000033222	Ttf2	1.535	1.04E-04	5.19E-03

ENSMUSG00000034522	Zfp395	1.556	1.96E-05	1.52E-03
ENSMUSG00000086587	Gm11837	1.559	1.85E-03	3.66E-02
ENSMUSG00000021775	Nr1d2	1.559	6.30E-06	6.61E-04
ENSMUSG00000028378	Ptgr1	1.560	2.66E-03	4.66E-02
ENSMUSG00000025404	R3hdm2	1.569	1.25E-04	5.92E-03
ENSMUSG00000029763	Exoc4	1.572	3.48E-04	1.21E-02
ENSMUSG00000026664	Phyh	1.580	3.61E-06	4.40E-04
ENSMUSG00000035765	Dym	1.587	2.88E-05	2.05E-03
ENSMUSG00000024866	Acy3	1.589	2.34E-04	9.16E-03
ENSMUSG00000033488	Cryzl2	1.592	2.31E-04	9.08E-03
ENSMUSG00000066705	Fxyd6	1.596	2.02E-03	3.86E-02
ENSMUSG00000032009	Sesn3	1.598	2.73E-04	1.02E-02
ENSMUSG00000022272	Myo10	1.602	9.50E-04	2.35E-02
ENSMUSG00000007837	Prrg2	1.605	2.33E-03	4.26E-02
ENSMUSG00000032121	Tmem218	1.606	2.08E-04	8.53E-03
ENSMUSG00000039745	Htatip2	1.609	1.74E-05	1.43E-03
ENSMUSG00000029385	Ccng2	1.609	7.32E-07	1.33E-04
ENSMUSG00000025993	Slc40a1	1.617	4.72E-07	9.53E-05
ENSMUSG00000002997	Prkar2b	1.631	1.10E-03	2.57E-02
ENSMUSG00000026939	Tmem141	1.638	5.66E-05	3.37E-03
ENSMUSG00000066357	Wdr6	1.643	2.00E-06	2.80E-04
ENSMUSG00000039671	Zmynd8	1.652	1.09E-03	2.56E-02
ENSMUSG00000039686	Zer1	1.664	2.13E-03	4.01E-02
ENSMUSG00000032067	Pts	1.671	4.64E-08	1.45E-05
ENSMUSG00000022587	Ly6e	1.673	3.97E-04	1.31E-02
ENSMUSG00000028789	Azin2	1.684	2.58E-03	4.55E-02
ENSMUSG00000041126	H2afv	1.687	1.22E-08	5.90E-06
ENSMUSG00000044986	Tst	1.688	1.07E-03	2.52E-02
ENSMUSG00000024646	Cyb5a	1.696	9.20E-04	2.31E-02
ENSMUSG00000020263	Appl2	1.701	4.92E-04	1.52E-02
ENSMUSG00000103962	Gm38213	1.702	2.17E-03	4.06E-02
ENSMUSG00000046329	Slc25a23	1.707	8.70E-04	2.25E-02
ENSMUSG00000058756	Thra	1.707	3.27E-04	1.17E-02
ENSMUSG00000028497	Hacd4	1.720	9.89E-04	2.41E-02
ENSMUSG00000042675	Ypel3	1.725	3.92E-09	2.25E-06
ENSMUSG00000024975	Pdcd4	1.726	1.02E-05	9.15E-04
ENSMUSG00000073755	5730409E04	1.726	1.31E-03	2.90E-02
ENSMUSG00000034908	Sidt2	1.727	2.06E-03	3.92E-02
ENSMUSG00000002055	Spag5	1.740	1.20E-03	2.74E-02
ENSMUSG00000021619	Atg10	1.740	8.46E-04	2.22E-02
ENSMUSG00000041219	Arhgap11a	1.742	1.38E-03	2.98E-02
ENSMUSG00000048581	E130311K13	1.744	2.54E-05	1.86E-03
ENSMUSG00000067336	Bmpr2	1.753	2.28E-03	4.22E-02
ENSMUSG00000020898	Ctc1	1.755	1.75E-05	1.43E-03
ENSMUSG00000022390	Zc3h7b	1.761	5.92E-04	1.74E-02
ENSMUSG00000038070	Cntln	1.770	2.05E-04	8.49E-03
ENSMUSG00000013236	Ptprs	1.792	1.50E-03	3.15E-02

ENSMUSG00000023505	Cdca3	1.798	1.29E-08	5.97E-06
ENSMUSG00000069835	Sat2	1.806	1.56E-03	3.23E-02
ENSMUSG00000022217	Emc9	1.807	5.08E-05	3.10E-03
ENSMUSG00000028597	Gpx7	1.810	2.45E-03	4.41E-02
ENSMUSG00000035642	Aamdc	1.816	4.10E-07	8.95E-05
ENSMUSG00000010362	Rdm1	1.821	4.59E-07	9.38E-05
ENSMUSG00000079598	Clec2l	1.823	1.43E-03	3.04E-02
ENSMUSG00000058135	Gstm1	1.831	1.36E-05	1.16E-03
ENSMUSG00000083307	AA414768	1.845	7.32E-04	2.00E-02
ENSMUSG00000032218	Ccnb2	1.848	2.41E-06	3.19E-04
ENSMUSG00000085457	1110046J04I	1.850	1.08E-04	5.30E-03
ENSMUSG00000038248	Sobp	1.869	4.24E-04	1.37E-02
ENSMUSG00000036523	Greb1	1.870	2.13E-03	4.01E-02
ENSMUSG00000107369	Gstm2-ps1	1.887	8.09E-06	7.69E-04
ENSMUSG00000029521	Chek2	1.889	2.22E-05	1.69E-03
ENSMUSG00000060985	Tdrd5	1.898	4.01E-04	1.32E-02
ENSMUSG00000039450	Dcxr	1.923	2.57E-08	9.66E-06
ENSMUSG00000031453	Rasa3	1.929	6.38E-06	6.67E-04
ENSMUSG00000003849	Nqo1	1.932	2.02E-03	3.86E-02
ENSMUSG00000040557	Mettl27	1.949	4.37E-08	1.42E-05
ENSMUSG00000000538	Tom1l2	1.955	1.42E-03	3.01E-02
ENSMUSG00000112876	Gm32443	1.958	1.08E-03	2.54E-02
ENSMUSG00000027227	Sord	1.961	2.00E-03	3.84E-02
ENSMUSG00000040296	Ddx58	1.971	1.02E-03	2.45E-02
ENSMUSG00000028199	Cryz	1.978	7.41E-05	4.07E-03
ENSMUSG00000027654	Fam83d	1.985	1.15E-06	1.84E-04
ENSMUSG00000035168	Tanc1	1.988	6.27E-04	1.81E-02
ENSMUSG00000094910	D430019H16	1.990	5.24E-04	1.60E-02
ENSMUSG00000068758	Il3ra	1.994	1.53E-03	3.20E-02
ENSMUSG00000033715	Akr1c14	1.994	1.57E-04	7.04E-03
ENSMUSG00000038252	Ncapd2	2.002	1.09E-04	5.31E-03
ENSMUSG00000030319	Cand2	2.005	6.83E-04	1.91E-02
ENSMUSG00000025545	Clybl	2.034	7.90E-06	7.61E-04
ENSMUSG00000020388	Pdlim4	2.037	1.83E-05	1.47E-03
ENSMUSG00000040249	Lrp1	2.037	1.60E-04	7.15E-03
ENSMUSG00000021065	Fut8	2.038	1.77E-05	1.44E-03
ENSMUSG00000021884	Hacl1	2.038	1.16E-05	1.02E-03
ENSMUSG00000087336	Gm15860	2.060	3.19E-04	1.15E-02
ENSMUSG00000085614	1700123M08	2.069	6.04E-04	1.76E-02
ENSMUSG00000024940	Ltbp3	2.075	1.46E-03	3.07E-02
ENSMUSG00000086382	Chrna1os	2.087	1.95E-04	8.14E-03
ENSMUSG00000046718	Bst2	2.092	1.23E-04	5.87E-03
ENSMUSG00000049086	Bmyc	2.103	2.10E-04	8.59E-03
ENSMUSG00000020486	45173	2.106	6.68E-04	1.88E-02
ENSMUSG00000027983	Cyp2u1	2.110	6.92E-04	1.92E-02
ENSMUSG00000059645	Gm7361	2.113	7.55E-04	2.04E-02
ENSMUSG00000041000	Trim62	2.123	2.27E-04	9.03E-03

ENSMUSG00000010307	Tmem86a	2.125	7.01E-05	3.89E-03
ENSMUSG00000022773	Ypel1	2.137	4.19E-07	9.04E-05
ENSMUSG00000026104	Stat1	2.155	9.83E-04	2.39E-02
ENSMUSG00000091561	Gm6665	2.158	5.20E-06	5.82E-04
ENSMUSG00000032224	Fam81a	2.159	1.40E-03	2.99E-02
ENSMUSG00000004035	Gstm7	2.164	1.44E-03	3.05E-02
ENSMUSG00000057143	Trim12c	2.188	2.06E-03	3.92E-02
ENSMUSG00000040562	Gstm2	2.201	5.70E-06	6.19E-04
ENSMUSG00000044167	Foxo1	2.223	1.76E-04	7.64E-03
ENSMUSG00000021094	Dhrs7	2.228	1.53E-03	3.19E-02
ENSMUSG00000010651	Acaa1b	2.244	2.19E-03	4.09E-02
ENSMUSG00000031666	Rbl2	2.254	1.27E-06	1.99E-04
ENSMUSG00000052676	Zmat1	2.265	2.42E-03	4.39E-02
ENSMUSG00000110935	Gm8834	2.278	1.99E-04	8.32E-03
ENSMUSG00000027890	Gstm4	2.289	6.74E-06	6.91E-04
ENSMUSG00000060675	Pla2g16	2.292	2.71E-06	3.48E-04
ENSMUSG00000014164	Klhl3	2.304	2.19E-04	8.81E-03
ENSMUSG00000001313	Rnd2	2.316	2.15E-03	4.03E-02
ENSMUSG00000031310	Zmym3	2.329	5.61E-05	3.34E-03
ENSMUSG00000033610	Pank1	2.333	1.31E-03	2.90E-02
ENSMUSG00000032221	Mns1	2.338	4.30E-05	2.71E-03
ENSMUSG00000111080	Gm20300	2.349	1.15E-05	1.02E-03
ENSMUSG00000098318	Lockd	2.357	1.86E-05	1.47E-03
ENSMUSG00000068551	Zfp467	2.385	2.21E-03	4.10E-02
ENSMUSG00000057176	Ccdc189	2.385	1.04E-03	2.48E-02
ENSMUSG00000054612	Mgmt	2.414	4.48E-10	3.37E-07
ENSMUSG00000064215	Ifi27	2.416	5.36E-13	1.17E-09
ENSMUSG00000041476	Smpx	2.418	1.26E-05	1.10E-03
ENSMUSG00000033420	Antxr1	2.434	9.57E-04	2.36E-02
ENSMUSG00000100155	Gm2109	2.444	2.93E-03	4.96E-02
ENSMUSG00000078350	Smim1	2.483	6.45E-04	1.84E-02
ENSMUSG00000079491	H2-T10	2.490	5.10E-04	1.57E-02
ENSMUSG00000051048	P4ha3	2.499	5.37E-05	3.24E-03
ENSMUSG00000105471	A430073D23	2.517	2.65E-04	9.99E-03
ENSMUSG00000073758	Sh3d21	2.521	1.37E-04	6.34E-03
ENSMUSG00000026875	Traf1	2.523	1.35E-03	2.96E-02
ENSMUSG00000020216	Jsrp1	2.526	7.54E-06	7.36E-04
ENSMUSG00000027253	Lrp4	2.554	1.72E-03	3.47E-02
ENSMUSG00000087380	2210408F21	2.556	1.04E-04	5.19E-03
ENSMUSG00000027966	Col11a1	2.592	9.85E-05	5.04E-03
ENSMUSG00000027330	Cdc25b	2.613	8.91E-06	8.27E-04
ENSMUSG00000028015	Ctso	2.619	7.38E-04	2.01E-02
ENSMUSG00000001520	Nrip2	2.634	1.31E-04	6.13E-03
ENSMUSG00000062901	Klhl24	2.649	1.59E-10	1.65E-07
ENSMUSG00000055692	Tmem191c	2.662	8.11E-04	2.15E-02
ENSMUSG00000085829	Gm4285	2.662	8.06E-04	2.14E-02
ENSMUSG00000002409	Dyrk1b	2.668	2.71E-04	1.02E-02

ENSMUSG00000034664	Itga2b	2.697	2.45E-03	4.41E-02
ENSMUSG00000037440	Vnn1	2.728	1.01E-03	2.44E-02
ENSMUSG00000103839	Gm37607	2.738	8.42E-04	2.21E-02
ENSMUSG00000036545	Adamts2	2.764	6.83E-05	3.83E-03
ENSMUSG00000022389	Tef	2.822	7.99E-07	1.38E-04
ENSMUSG00000085315	A430018G15	2.824	5.52E-04	1.65E-02
ENSMUSG00000035407	Kank4	2.834	1.32E-03	2.92E-02
ENSMUSG00000021416	Eci3	2.843	1.01E-03	2.44E-02
ENSMUSG00000040483	Xaf1	2.864	2.88E-03	4.90E-02
ENSMUSG00000041132	N4bp2l1	2.884	7.00E-04	1.94E-02
ENSMUSG00000019301	Hsd17b1	2.910	1.99E-03	3.83E-02
ENSMUSG00000097403	9230116N13	2.920	1.02E-03	2.45E-02
ENSMUSG00000113031	Gm34552	2.924	2.09E-03	3.97E-02
ENSMUSG00000001663	Gstt1	2.927	4.49E-06	5.21E-04
ENSMUSG00000030793	Pycard	2.953	4.65E-06	5.32E-04
ENSMUSG00000067698	Gm10220	2.994	4.54E-05	2.83E-03
ENSMUSG00000039533	Mmd2	3.006	6.58E-04	1.86E-02
ENSMUSG00000102979	Gm37350	3.010	2.70E-03	4.70E-02
ENSMUSG00000034706	Dnaic2	3.018	1.97E-03	3.80E-02
ENSMUSG00000020962	Gtf2a1	3.037	1.81E-06	2.60E-04
ENSMUSG00000112646	Gm5654	3.054	2.07E-04	8.53E-03
ENSMUSG00000040666	Sh3bgr	3.069	8.25E-04	2.18E-02
ENSMUSG00000085776	5430402O13	3.088	1.05E-03	2.49E-02
ENSMUSG00000002265	Peg3	3.090	2.84E-03	4.87E-02
ENSMUSG00000030380	Mzf1	3.100	8.54E-04	2.23E-02
ENSMUSG00000031808	Slc27a1	3.100	1.58E-03	3.26E-02
ENSMUSG00000067700	Gm5862	3.112	1.16E-03	2.66E-02
ENSMUSG00000044122	Proca1	3.137	9.20E-04	2.31E-02
ENSMUSG00000116097	AL590144.2	3.143	9.45E-06	8.59E-04
ENSMUSG00000028211	Trp53inp1	3.146	8.11E-06	7.69E-04
ENSMUSG00000036086	Zranb3	3.157	9.20E-04	2.31E-02
ENSMUSG00000035509	Fbxl21	3.166	2.51E-03	4.48E-02
ENSMUSG00000022235	Cmbl	3.172	1.16E-07	3.25E-05
ENSMUSG00000027217	Tspan18	3.179	3.74E-04	1.27E-02
ENSMUSG00000089686	Mnd1-ps	3.190	2.02E-03	3.87E-02
ENSMUSG00000003273	Car11	3.204	3.03E-04	1.10E-02
ENSMUSG00000001665	Gstt3	3.208	7.92E-06	7.61E-04
ENSMUSG00000024308	Tapbp	3.223	3.56E-04	1.22E-02
ENSMUSG00000028264	Spaca1	3.226	1.37E-03	2.98E-02
ENSMUSG00000039543	Cfap70	3.231	1.78E-03	3.57E-02
ENSMUSG00000038776	Ephx1	3.240	1.91E-03	3.73E-02
ENSMUSG00000107689	Gm44386	3.254	1.10E-06	1.80E-04
ENSMUSG00000097354	2310001H17	3.273	7.83E-04	2.09E-02
ENSMUSG00000066026	Dhrs3	3.302	6.44E-04	1.84E-02
ENSMUSG00000026817	Ak1	3.325	9.24E-06	8.47E-04
ENSMUSG00000022620	Arsa	3.341	2.60E-03	4.59E-02
ENSMUSG00000054580	Pla2r1	3.362	1.80E-03	3.60E-02

ENSMUSG00000030257	Srgap3	3.381	2.87E-04	1.06E-02
ENSMUSG00000034949	Zfr2	3.444	2.09E-03	3.97E-02
ENSMUSG00000027489	Necab3	3.445	1.04E-03	2.48E-02
ENSMUSG00000041536	Serpina3a	3.466	2.76E-04	1.03E-02
ENSMUSG00000021708	Rasgrf2	3.479	2.93E-03	4.96E-02
ENSMUSG00000022438	Parvb	3.499	2.11E-04	8.60E-03
ENSMUSG00000096727	Psmb9	3.533	7.65E-07	1.35E-04
ENSMUSG00000099354	1700124L16	3.561	9.55E-04	2.36E-02
ENSMUSG00000086003	B230206L02	3.602	1.41E-03	3.01E-02
ENSMUSG00000074771	Ankef1	3.614	1.92E-03	3.74E-02
ENSMUSG00000073821	8030451A03	3.673	9.90E-04	2.41E-02
ENSMUSG00000038453	Srcin1	3.706	6.85E-04	1.91E-02
ENSMUSG00000037709	Fam13a	3.721	6.34E-04	1.82E-02
ENSMUSG00000086900	Kcnab3os	3.725	1.04E-03	2.48E-02
ENSMUSG00000024818	Slc25a45	3.732	1.71E-04	7.46E-03
ENSMUSG00000027871	Hsd3b1	3.734	1.26E-08	5.97E-06
ENSMUSG00000026807	Ak8	3.756	7.05E-04	1.95E-02
ENSMUSG00000085354	Gm2044	3.766	1.14E-03	2.64E-02
ENSMUSG00000030708	Dnajb13	3.770	2.69E-03	4.70E-02
ENSMUSG00000028415	Spink4	3.776	1.41E-03	3.01E-02
ENSMUSG00000027870	Hao2	3.790	6.12E-04	1.78E-02
ENSMUSG00000110489	Gm31659	3.808	2.32E-03	4.25E-02
ENSMUSG00000085055	Gm15958	3.817	5.45E-04	1.64E-02
ENSMUSG00000086946	Gm15527	3.838	2.69E-03	4.70E-02
ENSMUSG00000026489	Coq8a	3.858	1.63E-13	5.91E-10
ENSMUSG00000020096	Tbata	3.866	5.28E-04	1.60E-02
ENSMUSG00000036585	Fgf1	3.869	1.53E-03	3.19E-02
ENSMUSG00000026831	1700007K13	3.885	7.79E-04	2.09E-02
ENSMUSG00000019933	Mrln	3.909	1.95E-03	3.77E-02
ENSMUSG00000048458	Fam212b	3.919	8.83E-04	2.27E-02
ENSMUSG00000074218	Cox7a1	3.921	2.03E-08	8.49E-06
ENSMUSG00000053388	Trim50	3.938	1.83E-03	3.64E-02
ENSMUSG00000074968	Ano3	3.941	2.46E-03	4.42E-02
ENSMUSG00000086735	Gm13977	3.946	2.30E-03	4.22E-02
ENSMUSG00000025468	Caly	3.949	8.82E-05	4.67E-03
ENSMUSG00000025934	Gsta3	3.961	4.81E-13	1.17E-09
ENSMUSG00000028931	Kcnab2	3.987	1.22E-03	2.75E-02
ENSMUSG00000112736	Gm48718	3.991	7.43E-04	2.02E-02
ENSMUSG00000028871	Rspo1	3.996	5.84E-05	3.45E-03
ENSMUSG00000038415	Foxq1	4.012	1.19E-03	2.72E-02
ENSMUSG00000068245	Phf11d	4.023	2.81E-03	4.82E-02
ENSMUSG00000057948	Unc13d	4.025	3.96E-06	4.70E-04
ENSMUSG00000022236	Ropn1l	4.043	3.46E-04	1.21E-02
ENSMUSG00000114617	Gm48766	4.046	2.69E-03	4.69E-02
ENSMUSG00000072941	Sod3	4.048	2.52E-04	9.62E-03
ENSMUSG00000039217	Il18	4.056	1.41E-03	3.01E-02
ENSMUSG00000026785	Pkn3	4.056	1.39E-03	2.99E-02

ENSMUSG00000074678	Defb25	4.062	1.93E-03	3.75E-02
ENSMUSG00000017453	Pipox	4.062	5.55E-04	1.66E-02
ENSMUSG00000023935	Spats1	4.072	3.13E-05	2.17E-03
ENSMUSG00000086813	Gm13657	4.092	2.64E-04	9.99E-03
ENSMUSG00000032343	Impg1	4.101	7.51E-04	2.03E-02
ENSMUSG00000039110	Mycbpap	4.112	8.64E-04	2.24E-02
ENSMUSG00000099583	Hist1h3d	4.143	8.02E-04	2.14E-02
ENSMUSG00000108592	Gm38973	4.174	2.53E-03	4.50E-02
ENSMUSG00000058656	Samd12	4.235	1.74E-03	3.51E-02
ENSMUSG00000033849	B3galt2	4.250	2.42E-03	4.38E-02
ENSMUSG00000001985	Grik3	4.259	4.15E-04	1.35E-02
ENSMUSG00000027347	Rasgrp1	4.259	1.11E-03	2.58E-02
ENSMUSG00000086405	9330198N18	4.276	2.76E-03	4.76E-02
ENSMUSG00000075410	Prcd	4.289	6.18E-07	1.19E-04
ENSMUSG00000108415	Gm30146	4.294	1.60E-03	3.29E-02
ENSMUSG00000038264	Sema7a	4.317	1.65E-05	1.36E-03
ENSMUSG00000033082	Clec1a	4.321	9.45E-04	2.34E-02
ENSMUSG00000012819	Cdh23	4.323	4.51E-05	2.82E-03
ENSMUSG00000079017	Ifi27l2a	4.328	3.25E-06	4.03E-04
ENSMUSG00000074595	Wfdc6a	4.357	5.80E-06	6.24E-04
ENSMUSG00000040812	Agbl2	4.359	7.05E-04	1.95E-02
ENSMUSG00000058952	Cfi	4.360	9.65E-05	4.97E-03
ENSMUSG00000038763	Alpk3	4.363	1.37E-03	2.98E-02
ENSMUSG00000022763	Aifm3	4.393	2.43E-03	4.40E-02
ENSMUSG00000043664	Tmem221	4.408	1.74E-03	3.51E-02
ENSMUSG00000032908	Sgpp2	4.417	9.55E-04	2.36E-02
ENSMUSG00000038246	Fam50b	4.425	1.89E-03	3.71E-02
ENSMUSG00000026241	Nppc	4.436	2.56E-03	4.54E-02
ENSMUSG00000036395	Glb1l2	4.503	9.29E-04	2.32E-02
ENSMUSG00000073413	Ly6g6d	4.508	3.27E-04	1.17E-02
ENSMUSG00000101378	Gm28086	4.513	6.79E-05	3.83E-03
ENSMUSG00000055809	Dnaaf3	4.527	1.41E-03	3.01E-02
ENSMUSG00000030935	Acsm3	4.547	5.81E-04	1.71E-02
ENSMUSG00000021185	Dglucy	4.574	3.57E-07	8.03E-05
ENSMUSG00000020963	Tshr	4.597	2.76E-03	4.76E-02
ENSMUSG00000035948	Acss3	4.611	8.64E-04	2.24E-02
ENSMUSG00000027656	Wisp2	4.623	1.65E-03	3.37E-02
ENSMUSG00000030474	Siglece	4.628	1.89E-04	7.99E-03
ENSMUSG00000086993	Rsf1os2	4.637	1.17E-03	2.67E-02
ENSMUSG00000041193	Pla2g5	4.721	1.37E-03	2.98E-02
ENSMUSG00000023153	Tmem52	4.732	2.51E-03	4.48E-02
ENSMUSG00000047591	Mafa	4.747	1.26E-03	2.81E-02
ENSMUSG00000055632	Hmcn2	4.762	2.39E-06	3.17E-04
ENSMUSG00000085419	Gm11734	4.796	7.16E-05	3.96E-03
ENSMUSG00000084942	7330404K18	4.798	1.44E-03	3.04E-02
ENSMUSG00000081670	Gm15697	4.816	1.86E-03	3.67E-02
ENSMUSG00000054763	Defb42	4.822	2.00E-04	8.34E-03

ENSMUSG00000092592	Gm20449	4.824	2.49E-03	4.46E-02
ENSMUSG00000112765	9430078K24	4.882	3.52E-04	1.21E-02
ENSMUSG00000089671	Gm16537	4.896	7.10E-05	3.93E-03
ENSMUSG00000042429	Adora1	4.908	4.92E-04	1.52E-02
ENSMUSG00000079363	Gbp4	4.925	8.73E-04	2.25E-02
ENSMUSG00000065715	Snord7	4.934	1.48E-04	6.74E-03
ENSMUSG00000028927	Padi2	4.937	2.02E-03	3.86E-02
ENSMUSG00000035916	Ptprq	4.939	4.87E-04	1.51E-02
ENSMUSG00000071531	Gprin2	4.945	7.29E-04	2.00E-02
ENSMUSG00000054418	2900041M22	5.039	5.69E-04	1.69E-02
ENSMUSG00000097459	Gm26895	5.041	6.89E-04	1.92E-02
ENSMUSG00000049625	Tifab	5.059	2.55E-03	4.52E-02
ENSMUSG00000111329	A830035O19	5.064	4.47E-04	1.42E-02
ENSMUSG00000097399	Gm26555	5.079	9.14E-04	2.31E-02
ENSMUSG00000065952	C330021F23	5.084	4.18E-04	1.35E-02
ENSMUSG00000113771	Gm36529	5.119	7.88E-04	2.11E-02
ENSMUSG00000025329	Padi1	5.122	2.20E-03	4.09E-02
ENSMUSG00000107876	Gm43936	5.125	2.31E-04	9.08E-03
ENSMUSG00000084411	Gm11510	5.128	6.67E-04	1.88E-02
ENSMUSG00000030317	Timp4	5.133	2.43E-05	1.80E-03
ENSMUSG00000112833	Gm36595	5.135	2.59E-03	4.58E-02
ENSMUSG00000109770	Gm30085	5.153	1.93E-03	3.75E-02
ENSMUSG00000058966	Fam57b	5.171	2.98E-04	1.09E-02
ENSMUSG00000027577	Chrna4	5.177	9.64E-04	2.36E-02
ENSMUSG00000108446	Gm44997	5.180	1.08E-04	5.30E-03
ENSMUSG00000073991	Cnbd1	5.203	2.24E-03	4.15E-02
ENSMUSG00000035686	Thrsp	5.218	4.35E-05	2.73E-03
ENSMUSG00000087175	Gm15133	5.225	6.72E-04	1.89E-02
ENSMUSG00000103572	Gm37115	5.233	8.25E-04	2.18E-02
ENSMUSG00000034829	Nxnl1	5.242	4.52E-04	1.43E-02
ENSMUSG00000026222	Sp100	5.246	1.86E-03	3.67E-02
ENSMUSG00000048038	Ccdc187	5.315	1.04E-04	5.19E-03
ENSMUSG00000049555	Tmie	5.337	1.71E-04	7.46E-03
ENSMUSG00000069814	Ccdc92b	5.339	2.30E-04	9.08E-03
ENSMUSG00000111256	Gm48094	5.358	2.50E-03	4.47E-02
ENSMUSG00000016942	Tmprss6	5.446	2.36E-04	9.19E-03
ENSMUSG00000030217	Art4	5.458	3.34E-05	2.29E-03
ENSMUSG00000096997	Gm26791	5.477	1.83E-03	3.63E-02
ENSMUSG00000104011	Gm32391	5.510	1.92E-04	8.06E-03
ENSMUSG00000039264	Gimap3	5.523	1.70E-03	3.45E-02
ENSMUSG00000103062	Gm37200	5.529	2.27E-03	4.20E-02
ENSMUSG00000078998	Bpifa6	5.538	1.82E-03	3.62E-02
ENSMUSG00000113687	AC123705.1	5.553	2.60E-03	4.59E-02
ENSMUSG00000112220	Gm36208	5.572	2.94E-05	2.08E-03
ENSMUSG00000033182	Kbtbd12	5.573	3.64E-04	1.24E-02
ENSMUSG00000100198	1700030O20	5.615	6.29E-04	1.81E-02
ENSMUSG00000105651	1700017M07	5.621	2.50E-03	4.46E-02

ENSMUSG00000116903	AC161607.1	5.625	2.59E-04	9.83E-03
ENSMUSG00000090164	BC035044	5.626	3.87E-04	1.30E-02
ENSMUSG00000038132	Rbm24	5.651	2.20E-03	4.09E-02
ENSMUSG00000087476	Rap1gapos	5.658	8.37E-04	2.20E-02
ENSMUSG00000007279	Scube2	5.670	4.97E-05	3.05E-03
ENSMUSG00000078653	Cntd1	5.680	3.24E-04	1.16E-02
ENSMUSG00000021223	Papln	5.682	8.03E-04	2.14E-02
ENSMUSG00000079013	Serpina3j	5.686	9.09E-06	8.37E-04
ENSMUSG00000087612	A230005M16	5.692	4.55E-06	5.23E-04
ENSMUSG00000035000	Dpp4	5.705	1.49E-03	3.13E-02
ENSMUSG00000027220	Syt13	5.705	9.80E-04	2.39E-02
ENSMUSG00000048442	Smim5	5.754	5.43E-04	1.64E-02
ENSMUSG00000004359	Spic	5.781	4.16E-04	1.35E-02
ENSMUSG00000018919	Tm4sf5	5.791	6.60E-04	1.86E-02
ENSMUSG00000109231	Gm45737	5.797	1.64E-03	3.36E-02
ENSMUSG00000006784	Ttc25	5.807	3.61E-04	1.24E-02
ENSMUSG00000085860	2410003L11	5.812	2.39E-04	9.23E-03
ENSMUSG00000074623	Gm826	5.812	4.28E-05	2.71E-03
ENSMUSG00000079547	H2-DMb1	5.828	1.10E-03	2.57E-02
ENSMUSG00000060441	Trim5	5.830	1.22E-04	5.84E-03
ENSMUSG00000038233	Fam198a	5.856	2.38E-04	9.22E-03
ENSMUSG00000030707	Coro1a	5.885	2.25E-03	4.17E-02
ENSMUSG00000083827	Gm15712	5.914	2.11E-03	3.99E-02
ENSMUSG00000114150	Gm46367	5.940	4.70E-04	1.47E-02
ENSMUSG00000060791	Gmfg	5.941	7.31E-06	7.29E-04
ENSMUSG00000102344	9430053O09	5.943	1.14E-03	2.63E-02
ENSMUSG00000081058	Hist2h3c2	5.970	5.04E-04	1.55E-02
ENSMUSG00000102544	Gm5103	5.970	4.64E-04	1.46E-02
ENSMUSG00000069385	Gm10267	5.997	1.06E-03	2.50E-02
ENSMUSG00000085772	D630024D03	6.009	1.50E-04	6.83E-03
ENSMUSG00000097717	1700066J03I	6.026	1.53E-04	6.90E-03
ENSMUSG00000114939	Gm48566	6.028	1.69E-03	3.44E-02
ENSMUSG00000113974	4930442G10	6.050	2.40E-04	9.27E-03
ENSMUSG00000002190	Clgn	6.093	4.29E-04	1.38E-02
ENSMUSG00000024164	C3	6.116	5.38E-04	1.63E-02
ENSMUSG00000067704	Wfdc13	6.130	1.34E-03	2.95E-02
ENSMUSG00000035208	Slfn8	6.163	2.29E-03	4.22E-02
ENSMUSG00000048231	H2-M10.4	6.169	4.23E-04	1.37E-02
ENSMUSG00000043953	Ccrl2	6.172	1.00E-03	2.43E-02
ENSMUSG00000031089	Slc6a14	6.205	7.01E-04	1.94E-02
ENSMUSG00000035594	Chrna5	6.230	3.90E-04	1.30E-02
ENSMUSG00000072852	2310040G07	6.273	1.49E-04	6.79E-03
ENSMUSG00000029228	Lnx1	6.277	2.19E-04	8.81E-03
ENSMUSG00000100815	Gm29112	6.292	9.03E-04	2.30E-02
ENSMUSG00000116288	AC116487.2	6.403	1.82E-04	7.80E-03
ENSMUSG00000108750	Gm44750	6.549	1.86E-03	3.67E-02
ENSMUSG00000062488	Ifit3b	6.555	1.06E-03	2.50E-02

ENSMUSG00000004892	Bcan	6.578	2.10E-07	5.27E-05
ENSMUSG00000068463	B630019A10	6.636	8.18E-05	4.36E-03
ENSMUSG00000087132	A930001C03	6.647	4.29E-07	9.08E-05
ENSMUSG00000059625	Sohlh1	6.657	3.00E-05	2.10E-03
ENSMUSG00000041550	Serpina5	6.704	7.40E-04	2.01E-02
ENSMUSG00000029343	Crybb1	6.750	3.74E-05	2.51E-03
ENSMUSG00000047085	Lrrc4b	6.944	2.42E-05	1.80E-03
ENSMUSG00000030732	Chrdl2	7.003	2.91E-04	1.07E-02
ENSMUSG00000104109	Gm30292	7.038	1.14E-05	1.01E-03
ENSMUSG00000037188	Grhl3	7.097	2.92E-05	2.07E-03
ENSMUSG00000090667	Gm765	7.140	3.42E-04	1.20E-02
ENSMUSG00000024245	Tmem178	7.179	3.04E-09	1.79E-06
ENSMUSG00000050700	Emilin3	7.262	7.26E-05	3.99E-03
ENSMUSG00000047343	Mettl21c	7.671	7.25E-06	7.26E-04
ENSMUSG00000002324	Rec8	7.790	8.74E-06	8.15E-04
ENSMUSG00000085121	5730437C11	7.964	2.06E-06	2.86E-04
ENSMUSG00000020928	Higd1b	17.753	2.64E-03	4.63E-02
ENSMUSG00000070423	Olfr558	18.332	1.80E-03	3.61E-02

Supplementary Table S5.

List of DEGs between 3h-24h in 2%

Ensembl_ID	SYMBOL	og2FoldChang	pvalue	padj
ENSMUSG00000085183	Wincr1	-22.053	5.26E-28	1.02E-23
ENSMUSG00000110163	Gm45389	-18.287	1.88E-03	3.87E-02
ENSMUSG00000050578	Mmp13	-10.837	3.98E-10	2.65E-07
ENSMUSG00000021680	Crhbp	-10.495	3.66E-09	1.81E-06
ENSMUSG00000115620	Gm45924	-8.719	9.48E-05	4.81E-03
ENSMUSG00000050359	Sprr1a	-8.526	7.92E-14	1.39E-10
ENSMUSG00000020911	Krt19	-8.078	2.85E-04	1.04E-02
ENSMUSG00000062345	Serpinb2	-8.021	1.23E-06	1.94E-04
ENSMUSG00000047562	Mmp10	-7.771	3.01E-04	1.08E-02
ENSMUSG00000021403	Serpinb9b	-7.348	8.96E-13	1.44E-09
ENSMUSG00000025582	Nptx1	-7.337	4.30E-04	1.37E-02
ENSMUSG00000022296	Baalc	-7.015	1.16E-05	9.65E-04
ENSMUSG00000029819	Npy	-6.829	5.84E-05	3.43E-03
ENSMUSG00000109903	Gm28710	-6.728	3.10E-04	1.10E-02
ENSMUSG00000024810	Il33	-6.671	4.17E-04	1.35E-02
ENSMUSG00000112120	Gm32255	-6.607	9.34E-04	2.43E-02
ENSMUSG00000021675	F2rl2	-6.603	2.05E-04	8.15E-03
ENSMUSG00000111467	Gm47775	-6.472	2.83E-04	1.03E-02
ENSMUSG00000029272	Sult1e1	-6.380	2.25E-03	4.43E-02
ENSMUSG00000041552	Ptchd1	-6.373	1.74E-03	3.68E-02
ENSMUSG00000044626	Liph	-6.317	1.13E-04	5.40E-03
ENSMUSG00000029304	Spp1	-5.985	6.48E-09	2.91E-06
ENSMUSG00000029378	Areg	-5.911	1.89E-03	3.87E-02
ENSMUSG00000031297	Slc7a3	-5.791	5.43E-11	5.00E-08
ENSMUSG00000026535	Ifi202b	-5.760	1.76E-07	3.96E-05
ENSMUSG00000026981	Il1rn	-5.715	6.36E-05	3.61E-03
ENSMUSG00000024912	Fosl1	-5.704	1.43E-07	3.47E-05
ENSMUSG00000010797	Wnt2	-5.529	7.57E-05	4.12E-03
ENSMUSG00000023905	Tnfrsf12a	-5.512	1.13E-22	1.09E-18
ENSMUSG00000019997	Ctgf	-5.485	3.17E-14	6.12E-11
ENSMUSG00000099974	Bcl2a1d	-5.460	3.40E-04	1.17E-02
ENSMUSG00000050335	Lgals3	-4.976	2.06E-14	4.97E-11
ENSMUSG00000028172	Tacr3	-4.913	1.55E-03	3.44E-02
ENSMUSG00000048482	Bdnf	-4.881	1.69E-06	2.51E-04
ENSMUSG00000029334	Prkg2	-4.816	2.45E-12	3.16E-09
ENSMUSG00000105528	Gm43519	-4.781	8.48E-04	2.26E-02
ENSMUSG00000041660	Bbox1	-4.720	8.28E-04	2.21E-02
ENSMUSG00000115009	G930009F23	-4.716	1.18E-03	2.83E-02
ENSMUSG00000063531	Sema3e	-4.683	4.29E-04	1.37E-02
ENSMUSG00000033730	Egr3	-4.679	6.88E-04	1.94E-02
ENSMUSG00000015652	Steap1	-4.663	1.15E-12	1.71E-09
ENSMUSG00000074199	Krtdap	-4.603	4.17E-05	2.62E-03

ENSMUSG00000037946	Fgd3	-4.450	7.32E-06	6.97E-04
ENSMUSG00000024640	Psat1	-4.404	4.81E-15	1.33E-11
ENSMUSG00000032487	Ptgs2	-4.396	5.09E-04	1.55E-02
ENSMUSG00000005148	Klf5	-4.393	1.63E-05	1.28E-03
ENSMUSG00000055407	Map6	-4.354	1.25E-04	5.63E-03
ENSMUSG00000029752	Asns	-4.296	2.58E-14	5.53E-11
ENSMUSG00000047171	Helt	-4.277	2.70E-04	9.97E-03
ENSMUSG00000032715	Trib3	-4.270	3.74E-08	1.25E-05
ENSMUSG00000023043	Krt18	-4.259	4.04E-09	1.95E-06
ENSMUSG00000021835	Bmp4	-4.259	1.46E-12	2.01E-09
ENSMUSG00000042734	Ttc9	-4.060	9.48E-04	2.46E-02
ENSMUSG00000028128	F3	-4.056	6.22E-08	1.79E-05
ENSMUSG00000006445	Epha2	-4.041	1.73E-03	3.67E-02
ENSMUSG00000046223	Plaur	-4.031	1.28E-08	5.17E-06
ENSMUSG00000115243	Gm5207	-4.015	3.00E-05	2.05E-03
ENSMUSG00000007655	Cav1	-3.994	6.69E-11	5.87E-08
ENSMUSG00000089812	Gm15867	-3.972	2.72E-05	1.90E-03
ENSMUSG00000027313	Chac1	-3.963	7.08E-08	1.95E-05
ENSMUSG00000029161	Cgref1	-3.941	4.37E-09	2.06E-06
ENSMUSG00000023046	Igfbp6	-3.938	4.25E-18	2.74E-14
ENSMUSG00000074813	Gm14005	-3.911	2.92E-05	2.00E-03
ENSMUSG00000024907	Gal	-3.880	2.67E-06	3.56E-04
ENSMUSG00000031530	Dusp4	-3.822	9.00E-05	4.65E-03
ENSMUSG00000028195	Cyr61	-3.802	2.29E-10	1.77E-07
ENSMUSG00000003541	Ier3	-3.782	1.68E-11	1.80E-08
ENSMUSG00000001021	S100a3	-3.736	3.09E-05	2.08E-03
ENSMUSG00000027562	Car2	-3.732	1.08E-04	5.25E-03
ENSMUSG00000078249	Hmga1b	-3.727	2.73E-08	9.78E-06
ENSMUSG00000022686	B3gnt5	-3.670	2.10E-03	4.20E-02
ENSMUSG00000022805	Maats1	-3.655	1.04E-03	2.62E-02
ENSMUSG00000028776	Tinagl1	-3.603	2.38E-09	1.25E-06
ENSMUSG00000026479	Lamc2	-3.544	7.35E-06	6.97E-04
ENSMUSG00000028480	Glipr2	-3.525	1.68E-03	3.59E-02
ENSMUSG00000037855	Zfp365	-3.517	7.53E-07	1.30E-04
ENSMUSG00000051111	Sv2c	-3.494	1.71E-04	7.20E-03
ENSMUSG00000020256	Aldh1l2	-3.473	2.26E-04	8.70E-03
ENSMUSG00000037868	Egr2	-3.462	1.66E-03	3.58E-02
ENSMUSG00000021822	Plau	-3.360	6.29E-05	3.59E-03
ENSMUSG00000005124	Wisp1	-3.310	1.78E-06	2.57E-04
ENSMUSG00000019960	Dusp6	-3.299	9.27E-07	1.53E-04
ENSMUSG00000030854	Ptpn5	-3.264	4.40E-04	1.39E-02
ENSMUSG00000030827	Fgf21	-3.240	1.94E-03	3.94E-02
ENSMUSG00000074657	Kif5a	-3.230	1.13E-03	2.77E-02
ENSMUSG00000046711	Hmga1	-3.222	3.71E-06	4.19E-04
ENSMUSG00000017144	Rnd3	-3.212	5.39E-07	1.00E-04
ENSMUSG00000042256	Ptchd4	-3.208	1.05E-03	2.64E-02
ENSMUSG00000000058	Cav2	-3.176	4.61E-08	1.42E-05

ENSMUSG00000110126	Gm9347	-3.162	4.48E-06	4.81E-04
ENSMUSG00000034271	Jdp2	-3.098	7.32E-06	6.97E-04
ENSMUSG00000032068	Plet1	-3.077	2.62E-03	4.92E-02
ENSMUSG00000057604	Lmcd1	-3.050	1.89E-05	1.42E-03
ENSMUSG00000029446	Psph	-2.990	1.79E-16	6.91E-13
ENSMUSG00000025140	Pycr1	-2.976	6.06E-04	1.77E-02
ENSMUSG00000030717	Nupr1	-2.905	3.49E-04	1.20E-02
ENSMUSG00000113769	5033406O09	-2.877	5.26E-06	5.50E-04
ENSMUSG00000062661	Ncs1	-2.857	2.73E-06	3.61E-04
ENSMUSG00000031434	Morc4	-2.779	4.66E-05	2.84E-03
ENSMUSG00000026547	Tagln2	-2.777	6.84E-04	1.94E-02
ENSMUSG00000115505	Gm9247	-2.751	2.46E-03	4.71E-02
ENSMUSG00000003283	Hck	-2.714	2.66E-03	4.98E-02
ENSMUSG00000028811	Yars	-2.711	8.32E-07	1.40E-04
ENSMUSG00000042549	Map2k3os	-2.706	1.29E-03	3.04E-02
ENSMUSG00000028680	Plk3	-2.689	7.13E-05	3.95E-03
ENSMUSG00000037960	Card19	-2.682	1.51E-08	5.94E-06
ENSMUSG00000020142	Slc1a4	-2.661	1.15E-03	2.80E-02
ENSMUSG00000039126	Prune2	-2.653	1.83E-05	1.39E-03
ENSMUSG00000015312	Gadd45b	-2.627	4.50E-07	8.98E-05
ENSMUSG00000083512	Gm12749	-2.615	1.98E-03	4.02E-02
ENSMUSG00000024587	Nars	-2.610	1.65E-07	3.79E-05
ENSMUSG00000029777	Gars	-2.568	1.50E-09	9.15E-07
ENSMUSG00000037060	Cavin3	-2.556	1.38E-03	3.18E-02
ENSMUSG00000036856	Wnt4	-2.541	2.60E-03	4.90E-02
ENSMUSG00000026784	Pdss1	-2.535	3.79E-12	4.58E-09
ENSMUSG00000031574	Star	-2.491	6.46E-06	6.33E-04
ENSMUSG00000006442	Srm	-2.471	4.47E-07	8.98E-05
ENSMUSG00000052688	Rab7b	-2.461	4.62E-07	9.11E-05
ENSMUSG00000053746	Ptrh1	-2.450	3.48E-06	4.07E-04
ENSMUSG00000073433	Arhgdig	-2.423	6.07E-07	1.10E-04
ENSMUSG00000022241	Tars	-2.422	3.61E-06	4.13E-04
ENSMUSG00000005667	Mthfd2	-2.414	9.69E-07	1.59E-04
ENSMUSG00000091898	Tnnc1	-2.413	1.70E-06	2.51E-04
ENSMUSG00000010755	Cars	-2.412	3.62E-10	2.50E-07
ENSMUSG00000025511	Tspan4	-2.400	4.67E-08	1.42E-05
ENSMUSG00000082674	Gm11914	-2.390	1.07E-03	2.67E-02
ENSMUSG00000011179	Odc1	-2.338	1.40E-10	1.13E-07
ENSMUSG00000101431	Gm7901	-2.334	4.16E-04	1.35E-02
ENSMUSG00000026749	Nek6	-2.330	8.78E-06	8.04E-04
ENSMUSG00000055737	Ghr	-2.329	9.24E-06	8.27E-04
ENSMUSG00000060950	Trmt61a	-2.329	1.81E-04	7.48E-03
ENSMUSG00000019970	Sgk1	-2.325	6.25E-04	1.80E-02
ENSMUSG00000031490	Eif4ebp1	-2.322	1.18E-05	9.74E-04
ENSMUSG00000053398	Phgdh	-2.321	5.32E-07	9.98E-05
ENSMUSG00000027610	Gss	-2.317	1.61E-07	3.76E-05
ENSMUSG00000025007	Aldh18a1	-2.308	1.42E-06	2.19E-04

ENSMUSG00000001025	S100a6	-2.297	2.65E-03	4.96E-02
ENSMUSG00000028063	Lmna	-2.247	1.28E-04	5.73E-03
ENSMUSG00000034765	Dusp5	-2.213	4.92E-06	5.17E-04
ENSMUSG00000038539	Atf5	-2.197	1.08E-05	9.16E-04
ENSMUSG00000032802	Srxn1	-2.188	5.89E-04	1.74E-02
ENSMUSG00000018849	Wwc1	-2.167	1.37E-03	3.16E-02
ENSMUSG00000050222	Il17d	-2.164	7.53E-04	2.06E-02
ENSMUSG00000038894	Irs2	-2.163	9.62E-05	4.84E-03
ENSMUSG00000028069	Gpatch4	-2.155	4.60E-08	1.42E-05
ENSMUSG00000083465	Gm11652	-2.148	9.03E-05	4.65E-03
ENSMUSG00000042743	Sgtb	-2.137	1.65E-03	3.58E-02
ENSMUSG00000068739	Sars	-2.130	3.33E-06	3.97E-04
ENSMUSG00000025403	Shmt2	-2.047	9.18E-06	8.27E-04
ENSMUSG00000025287	Acot9	-2.032	3.40E-06	4.00E-04
ENSMUSG00000098274	Rpl24	-2.029	3.54E-04	1.21E-02
ENSMUSG00000042515	Mum1l1	-2.025	2.35E-06	3.21E-04
ENSMUSG00000041506	Rrp9	-2.002	3.31E-06	3.97E-04
ENSMUSG00000025869	Nop16	-2.002	3.27E-06	3.97E-04
ENSMUSG00000002343	Armc6	-1.999	8.49E-04	2.26E-02
ENSMUSG00000032231	Anxa2	-1.982	4.07E-06	4.50E-04
ENSMUSG00000045763	Basp1	-1.979	8.78E-05	4.61E-03
ENSMUSG00000050017	Pitpnb	-1.968	3.19E-04	1.11E-02
ENSMUSG00000038335	Tsr1	-1.957	1.51E-09	9.15E-07
ENSMUSG00000020205	Phlda1	-1.954	1.43E-06	2.19E-04
ENSMUSG00000037601	Nme1	-1.949	5.86E-07	1.07E-04
ENSMUSG00000038279	Nop2	-1.945	1.58E-07	3.72E-05
ENSMUSG00000003348	Mob3a	-1.928	1.78E-03	3.74E-02
ENSMUSG00000056501	Cebpb	-1.927	1.74E-03	3.68E-02
ENSMUSG00000041774	Ydjc	-1.923	3.95E-04	1.31E-02
ENSMUSG00000028495	Rps6	-1.921	1.23E-05	1.00E-03
ENSMUSG00000053560	Ier2	-1.920	1.68E-03	3.59E-02
ENSMUSG00000029171	Pgm1	-1.909	1.76E-04	7.34E-03
ENSMUSG00000020303	Stc2	-1.908	1.18E-04	5.51E-03
ENSMUSG00000074220	Zfp382	-1.896	9.55E-04	2.47E-02
ENSMUSG00000028896	Rcc1	-1.887	4.09E-05	2.58E-03
ENSMUSG00000018932	Map2k3	-1.887	3.07E-04	1.09E-02
ENSMUSG00000000561	Wdr77	-1.885	1.82E-05	1.39E-03
ENSMUSG00000055044	Pdlim1	-1.882	1.29E-03	3.03E-02
ENSMUSG00000085156	Snhg15	-1.877	3.59E-04	1.22E-02
ENSMUSG00000063229	Ldha	-1.872	1.20E-04	5.57E-03
ENSMUSG00000085666	Gm9855	-1.871	2.44E-03	4.68E-02
ENSMUSG00000021831	Ero1l	-1.847	2.99E-05	2.04E-03
ENSMUSG00000048612	Myof	-1.825	2.80E-04	1.02E-02
ENSMUSG00000007041	Clic1	-1.811	2.55E-05	1.84E-03
ENSMUSG00000053801	Grwd1	-1.807	2.01E-06	2.83E-04
ENSMUSG00000047565	Acot10	-1.807	1.38E-03	3.19E-02
ENSMUSG00000039405	Prss23	-1.791	1.87E-03	3.86E-02

ENSMUSG00000027405	Nop56	-1.785	4.86E-08	1.42E-05
ENSMUSG00000010067	Rassf1	-1.779	9.09E-04	2.38E-02
ENSMUSG00000024037	Wdr4	-1.768	2.23E-04	8.66E-03
ENSMUSG00000063316	Rpl27	-1.763	4.07E-08	1.33E-05
ENSMUSG00000051223	Bzw1	-1.761	3.85E-05	2.46E-03
ENSMUSG00000037465	Klf10	-1.759	1.51E-03	3.37E-02
ENSMUSG00000101188	Eif4a-ps4	-1.754	5.30E-04	1.60E-02
ENSMUSG00000043192	Gm1840	-1.744	7.56E-04	2.06E-02
ENSMUSG00000002825	Qtrt1	-1.740	2.41E-04	9.19E-03
ENSMUSG00000049957	Ccdc137	-1.736	7.83E-05	4.21E-03
ENSMUSG00000032966	Fkbp1a	-1.734	1.75E-04	7.33E-03
ENSMUSG00000032051	Fdx1	-1.727	2.01E-03	4.07E-02
ENSMUSG00000008450	Nutf2	-1.698	7.47E-07	1.30E-04
ENSMUSG00000013089	Etv5	-1.692	2.28E-03	4.47E-02
ENSMUSG00000000078	Klf6	-1.687	6.76E-05	3.78E-03
ENSMUSG00000112903	Gm5948	-1.684	4.24E-04	1.36E-02
ENSMUSG00000095567	Noc2l	-1.669	3.69E-05	2.42E-03
ENSMUSG00000078812	Eif5a	-1.664	1.90E-04	7.77E-03
ENSMUSG00000022184	Fbxo4	-1.656	1.81E-06	2.60E-04
ENSMUSG00000052825	Gm9892	-1.655	5.45E-06	5.66E-04
ENSMUSG00000001305	Rrp15	-1.649	2.81E-05	1.95E-03
ENSMUSG00000024493	Lars	-1.642	6.50E-07	1.16E-04
ENSMUSG00000029500	Pgam5	-1.642	6.19E-05	3.57E-03
ENSMUSG00000042406	Atf4	-1.639	7.61E-05	4.13E-03
ENSMUSG00000028010	Gar1	-1.631	2.28E-06	3.12E-04
ENSMUSG00000085385	Snhg17	-1.626	9.84E-06	8.61E-04
ENSMUSG00000052151	Plpp2	-1.615	2.06E-05	1.53E-03
ENSMUSG00000074280	Gm6166	-1.612	2.09E-03	4.19E-02
ENSMUSG00000003848	Nob1	-1.602	4.32E-04	1.38E-02
ENSMUSG00000067161	Gm5560	-1.600	1.78E-03	3.73E-02
ENSMUSG00000033706	Smyd5	-1.594	2.08E-04	8.19E-03
ENSMUSG00000064264	Zfp428	-1.591	1.19E-07	2.99E-05
ENSMUSG00000024999	Noc3l	-1.580	2.67E-04	9.93E-03
ENSMUSG00000028466	Creb3	-1.561	9.62E-04	2.48E-02
ENSMUSG00000046865	Fbl	-1.558	2.07E-04	8.19E-03
ENSMUSG00000000916	Nsun5	-1.557	4.34E-04	1.38E-02
ENSMUSG00000040354	Mars	-1.551	7.84E-05	4.21E-03
ENSMUSG00000022554	Hgh1	-1.550	2.61E-07	5.49E-05
ENSMUSG00000031960	Aars	-1.547	3.97E-05	2.52E-03
ENSMUSG00000048007	Timm8a1	-1.542	2.65E-04	9.88E-03
ENSMUSG00000090243	Gm16103	-1.541	2.94E-04	1.05E-02
ENSMUSG00000028729	Ebna1bp2	-1.540	3.92E-07	8.05E-05
ENSMUSG00000005481	Ddx39	-1.540	1.07E-05	9.11E-04
ENSMUSG00000048261	Gm4879	-1.531	1.07E-03	2.66E-02
ENSMUSG00000021595	Nsun2	-1.529	2.25E-04	8.70E-03
ENSMUSG00000026234	Ncl	-1.522	1.10E-07	2.81E-05
ENSMUSG00000029430	Ran	-1.519	2.19E-08	8.29E-06

ENSMUSG00000028741	Mrto4	-1.510	9.00E-07	1.50E-04
ENSMUSG00000051557	Pusl1	-1.508	1.17E-03	2.82E-02
ENSMUSG00000057561	Eif1a	-1.505	1.10E-05	9.34E-04
ENSMUSG00000040463	Mybbp1a	-1.504	1.82E-07	4.04E-05
ENSMUSG00000029610	Aimp2	-1.498	8.28E-06	7.71E-04
ENSMUSG00000032078	Zpr1	-1.497	4.65E-04	1.45E-02
ENSMUSG00000024841	Eif1ad	-1.496	2.42E-06	3.27E-04
ENSMUSG00000026020	Nop58	-1.486	4.72E-09	2.17E-06
ENSMUSG00000030079	Ruvbl1	-1.482	1.16E-05	9.65E-04
ENSMUSG00000041360	Pum3	-1.478	7.05E-04	1.98E-02
ENSMUSG00000031578	Mak16	-1.475	1.01E-05	8.69E-04
ENSMUSG00000063524	Eno1	-1.475	5.40E-05	3.25E-03
ENSMUSG00000018446	C1qbp	-1.475	8.15E-08	2.19E-05
ENSMUSG00000026019	Wdr12	-1.472	1.07E-04	5.23E-03
ENSMUSG00000026670	Uap1	-1.469	3.34E-06	3.97E-04
ENSMUSG00000006732	Mettl1	-1.468	9.71E-06	8.57E-04
ENSMUSG00000026603	Smyd2	-1.463	1.09E-03	2.70E-02
ENSMUSG00000040675	Mthfd1l	-1.459	4.21E-08	1.36E-05
ENSMUSG00000024312	Wdr46	-1.458	2.12E-03	4.23E-02
ENSMUSG00000041057	Wdr43	-1.450	2.86E-06	3.72E-04
ENSMUSG00000025485	Ric8a	-1.449	1.12E-03	2.76E-02
ENSMUSG00000042215	Bag2	-1.445	9.62E-06	8.53E-04
ENSMUSG00000001380	Hars	-1.445	3.05E-04	1.09E-02
ENSMUSG00000038046	Mrm3	-1.444	3.17E-06	3.95E-04
ENSMUSG00000058355	Abce1	-1.442	2.52E-04	9.48E-03
ENSMUSG00000001627	Ifrd1	-1.440	1.50E-04	6.49E-03
ENSMUSG00000028982	Slc25a33	-1.439	5.60E-05	3.32E-03
ENSMUSG00000027944	Hax1	-1.438	2.06E-07	4.47E-05
ENSMUSG00000062867	Impdh2	-1.436	1.10E-03	2.72E-02
ENSMUSG00000039356	Exosc2	-1.428	9.87E-05	4.94E-03
ENSMUSG00000031432	Prps1	-1.422	2.97E-04	1.07E-02
ENSMUSG00000055612	Cdca7	-1.418	3.75E-05	2.43E-03
ENSMUSG00000031146	Plp2	-1.416	2.60E-04	9.74E-03
ENSMUSG00000022863	Btg3	-1.409	3.98E-04	1.31E-02
ENSMUSG00000028318	Polr1e	-1.405	1.45E-04	6.32E-03
ENSMUSG00000029447	Cct6a	-1.381	2.82E-05	1.95E-03
ENSMUSG00000020116	Pno1	-1.380	1.64E-06	2.46E-04
ENSMUSG00000026806	Ddx31	-1.379	3.10E-05	2.08E-03
ENSMUSG00000067367	Lyar	-1.375	2.03E-05	1.51E-03
ENSMUSG00000025981	Coq10b	-1.373	2.09E-04	8.21E-03
ENSMUSG00000020691	Mettl2	-1.370	5.31E-04	1.60E-02
ENSMUSG00000025980	Hspd1	-1.368	8.81E-12	1.00E-08
ENSMUSG00000027395	Polr1b	-1.367	1.13E-03	2.77E-02
ENSMUSG00000029036	Atad3a	-1.364	1.21E-04	5.57E-03
ENSMUSG00000041488	Stx3	-1.363	7.15E-06	6.87E-04
ENSMUSG00000042354	Gnl3	-1.362	4.88E-04	1.50E-02
ENSMUSG00000020089	Ppa1	-1.360	9.61E-09	4.04E-06

ENSMUSG00000040158	Tax1bp3	-1.351	2.10E-03	4.20E-02
ENSMUSG00000021149	Gtpbp4	-1.350	4.74E-08	1.42E-05
ENSMUSG00000070284	Gmppb	-1.350	2.28E-03	4.47E-02
ENSMUSG00000031568	Rwdd4a	-1.350	1.16E-04	5.48E-03
ENSMUSG00000036693	Nop14	-1.347	7.03E-08	1.95E-05
ENSMUSG00000062937	Mtap	-1.345	7.26E-04	2.02E-02
ENSMUSG00000038550	Ciart	-1.341	3.01E-04	1.08E-02
ENSMUSG00000071359	Tbpl1	-1.341	5.72E-04	1.70E-02
ENSMUSG00000038510	Rpf2	-1.337	4.82E-04	1.49E-02
ENSMUSG00000000339	Rtca	-1.336	3.15E-04	1.11E-02
ENSMUSG00000023988	Bysl	-1.335	1.50E-03	3.36E-02
ENSMUSG00000054836	Elp6	-1.329	2.23E-03	4.39E-02
ENSMUSG00000020358	Hnrnpab	-1.329	1.26E-04	5.67E-03
ENSMUSG00000031352	Hccs	-1.324	3.68E-04	1.24E-02
ENSMUSG00000027804	Ppid	-1.322	3.04E-05	2.06E-03
ENSMUSG00000021282	Eif5	-1.318	2.87E-06	3.72E-04
ENSMUSG00000025995	Wdr75	-1.315	6.04E-05	3.53E-03
ENSMUSG00000025591	Tma16	-1.310	3.25E-06	3.97E-04
ENSMUSG00000074656	Eif2s2	-1.310	1.84E-05	1.39E-03
ENSMUSG00000038388	Mpp6	-1.308	2.39E-03	4.64E-02
ENSMUSG00000026245	Farsb	-1.306	1.41E-03	3.23E-02
ENSMUSG00000031278	Acsl4	-1.306	3.55E-05	2.34E-03
ENSMUSG00000027236	Eif3j1	-1.302	2.30E-07	4.93E-05
ENSMUSG00000024360	Etf1	-1.302	1.78E-03	3.74E-02
ENSMUSG00000097195	Snhg5	-1.297	1.72E-04	7.24E-03
ENSMUSG00000062070	Pgk1	-1.295	5.09E-04	1.55E-02
ENSMUSG00000004127	Trmt10a	-1.294	1.87E-04	7.69E-03
ENSMUSG00000020869	Lrrc59	-1.293	1.27E-03	3.00E-02
ENSMUSG00000010095	Slc3a2	-1.287	3.79E-06	4.24E-04
ENSMUSG00000024726	Carnmt1	-1.283	5.10E-04	1.55E-02
ENSMUSG00000024359	Hspa9	-1.282	3.98E-07	8.09E-05
ENSMUSG00000068039	Tcp1	-1.278	8.71E-08	2.31E-05
ENSMUSG00000115219	Eef1akmt4	-1.268	5.87E-04	1.74E-02
ENSMUSG00000056962	Jmjd6	-1.267	2.04E-04	8.13E-03
ENSMUSG00000081999	Gm13461	-1.261	1.89E-04	7.75E-03
ENSMUSG00000021694	Ercc8	-1.261	2.82E-05	1.95E-03
ENSMUSG00000028683	Eif2b3	-1.260	9.19E-06	8.27E-04
ENSMUSG00000007029	Vars	-1.256	1.42E-04	6.23E-03
ENSMUSG00000035530	Eif1	-1.251	1.68E-07	3.81E-05
ENSMUSG00000020075	Ddx21	-1.248	4.72E-07	9.21E-05
ENSMUSG00000031917	Nip7	-1.247	5.78E-06	5.88E-04
ENSMUSG00000030057	Cnbp	-1.246	6.22E-05	3.57E-03
ENSMUSG00000061024	Rrs1	-1.244	1.13E-04	5.42E-03
ENSMUSG00000108414	Snhg1	-1.240	2.08E-03	4.17E-02
ENSMUSG00000029507	Pus1	-1.239	1.17E-03	2.82E-02
ENSMUSG00000022234	Cct5	-1.237	1.31E-05	1.05E-03
ENSMUSG00000028330	Ncbp1	-1.228	2.11E-04	8.25E-03

ENSMUSG00000024805	Pcgf5	-1.226	3.25E-04	1.13E-02
ENSMUSG00000025264	Tsr2	-1.225	1.09E-04	5.29E-03
ENSMUSG00000030007	Cct7	-1.224	1.21E-04	5.57E-03
ENSMUSG00000026615	Eprs	-1.218	1.05E-05	9.05E-04
ENSMUSG00000063888	Rpl7l1	-1.217	1.70E-03	3.62E-02
ENSMUSG00000026617	Bpnt1	-1.216	1.83E-05	1.39E-03
ENSMUSG00000034343	Ube2f	-1.209	9.86E-07	1.60E-04
ENSMUSG00000020484	Xbp1	-1.208	3.67E-04	1.24E-02
ENSMUSG00000001785	Pwp1	-1.207	2.56E-04	9.62E-03
ENSMUSG00000038274	Fau	-1.206	1.46E-07	3.49E-05
ENSMUSG00000021116	Eif2s1	-1.204	1.76E-04	7.34E-03
ENSMUSG00000046027	Stard5	-1.199	5.60E-04	1.67E-02
ENSMUSG00000030888	Rrp8	-1.196	6.04E-04	1.77E-02
ENSMUSG00000027613	Eif6	-1.195	1.66E-03	3.58E-02
ENSMUSG00000024732	Ccdc86	-1.194	3.65E-04	1.23E-02
ENSMUSG00000059796	Eif4a1	-1.190	1.83E-05	1.39E-03
ENSMUSG00000034203	Chchd4	-1.190	1.23E-06	1.94E-04
ENSMUSG00000034120	Srsf2	-1.188	4.10E-06	4.51E-04
ENSMUSG00000041747	Utp15	-1.187	2.91E-06	3.74E-04
ENSMUSG00000020648	Dus4l	-1.187	8.60E-04	2.28E-02
ENSMUSG00000023110	Prmt5	-1.184	1.13E-03	2.77E-02
ENSMUSG00000041453	Rpl21	-1.183	7.22E-04	2.01E-02
ENSMUSG00000070319	Eif3g	-1.179	1.43E-07	3.47E-05
ENSMUSG00000025134	Alyref	-1.174	1.51E-05	1.19E-03
ENSMUSG00000003868	Ruvbl2	-1.174	4.15E-04	1.35E-02
ENSMUSG00000030738	Eif3c	-1.166	1.28E-04	5.73E-03
ENSMUSG00000014226	Cacybp	-1.163	1.13E-03	2.77E-02
ENSMUSG00000029229	Chic2	-1.155	4.16E-04	1.35E-02
ENSMUSG00000016554	Eif3d	-1.155	2.33E-05	1.70E-03
ENSMUSG00000055723	Rras2	-1.146	1.90E-03	3.89E-02
ENSMUSG00000022407	Adsl	-1.134	9.92E-04	2.53E-02
ENSMUSG00000015176	Nolc1	-1.132	1.37E-03	3.16E-02
ENSMUSG00000025613	Cct8	-1.130	1.72E-06	2.51E-04
ENSMUSG00000038975	Rabggtb	-1.128	7.76E-05	4.20E-03
ENSMUSG00000025858	Get4	-1.125	1.36E-03	3.16E-02
ENSMUSG00000001707	Eef1e1	-1.123	7.23E-05	3.97E-03
ENSMUSG00000063884	Ptcd3	-1.118	1.85E-05	1.40E-03
ENSMUSG00000027669	Gnb4	-1.117	2.43E-03	4.67E-02
ENSMUSG00000006699	Cdc42	-1.108	1.51E-03	3.37E-02
ENSMUSG00000002881	Nab1	-1.107	2.30E-04	8.82E-03
ENSMUSG00000024528	Srfbp1	-1.106	3.18E-04	1.11E-02
ENSMUSG00000006599	Gtf2h1	-1.106	1.19E-03	2.85E-02
ENSMUSG00000043424	Eif3j2	-1.102	8.41E-06	7.78E-04
ENSMUSG00000001525	Tubb5	-1.101	3.80E-05	2.45E-03
ENSMUSG00000028007	Snx7	-1.096	2.47E-03	4.72E-02
ENSMUSG00000079435	Rpl36a	-1.094	2.19E-03	4.32E-02
ENSMUSG00000030264	Thumpd3	-1.093	4.87E-05	2.94E-03

ENSMUSG00000027808	Serp1	-1.091	4.89E-04	1.50E-02
ENSMUSG00000063694	Cycs	-1.090	1.80E-03	3.76E-02
ENSMUSG00000016319	Slc25a5	-1.086	2.47E-04	9.35E-03
ENSMUSG00000004264	Phb2	-1.079	1.69E-03	3.61E-02
ENSMUSG00000013701	Timm23	-1.078	1.82E-05	1.39E-03
ENSMUSG00000072235	Tuba1a	-1.077	1.09E-03	2.70E-02
ENSMUSG00000106988	Tsg101-ps	-1.076	7.45E-04	2.05E-02
ENSMUSG00000030759	Far1	-1.076	6.49E-05	3.67E-03
ENSMUSG00000011114	Tbrg1	-1.075	2.49E-03	4.74E-02
ENSMUSG00000037594	BC022687	-1.074	2.13E-03	4.24E-02
ENSMUSG00000007739	Cct4	-1.071	1.19E-04	5.53E-03
ENSMUSG00000081603	Gm14681	-1.071	6.55E-04	1.88E-02
ENSMUSG00000032279	Idh3a	-1.069	3.68E-06	4.18E-04
ENSMUSG00000026356	Dars	-1.069	1.66E-03	3.58E-02
ENSMUSG00000001674	Ddx18	-1.061	5.65E-05	3.34E-03
ENSMUSG00000022814	Umps	-1.060	1.15E-05	9.65E-04
ENSMUSG00000023883	Phf10	-1.060	3.45E-04	1.19E-02
ENSMUSG00000034681	Rnps1	-1.059	1.11E-03	2.75E-02
ENSMUSG00000030045	Mrpl19	-1.057	1.17E-03	2.82E-02
ENSMUSG00000028889	Yrdc	-1.053	1.10E-04	5.31E-03
ENSMUSG00000025630	Hprt	-1.052	1.41E-03	3.22E-02
ENSMUSG00000031657	Heatr3	-1.047	1.49E-03	3.34E-02
ENSMUSG00000001891	Ugp2	-1.047	1.04E-04	5.13E-03
ENSMUSG00000002660	Clpp	-1.040	2.35E-03	4.57E-02
ENSMUSG00000021958	Pinx1	-1.040	3.24E-04	1.12E-02
ENSMUSG00000025190	Got1	-1.039	7.23E-05	3.97E-03
ENSMUSG00000017561	Crlf3	-1.034	1.00E-03	2.55E-02
ENSMUSG00000071172	Srsf3	-1.032	1.75E-03	3.69E-02
ENSMUSG00000003808	Farsa	-1.027	1.32E-03	3.08E-02
ENSMUSG00000022752	Tomm70a	-1.027	7.51E-04	2.05E-02
ENSMUSG00000090137	Uba52	-1.027	5.66E-06	5.79E-04
ENSMUSG00000042719	Naa25	-1.022	1.23E-04	5.63E-03
ENSMUSG00000018965	Ywhah	-1.020	5.67E-04	1.69E-02
ENSMUSG00000026926	Pmpca	-1.017	3.11E-04	1.10E-02
ENSMUSG00000022792	Yars2	-1.017	6.25E-04	1.80E-02
ENSMUSG00000047649	Cd3eap	-1.016	7.58E-04	2.06E-02
ENSMUSG00000018697	Aatf	-1.014	4.68E-04	1.45E-02
ENSMUSG00000004393	Ddx56	-1.013	2.17E-03	4.29E-02
ENSMUSG00000028333	Anp32b	-1.013	8.30E-06	7.71E-04
ENSMUSG00000042225	Ammecr1	-1.011	1.49E-04	6.43E-03
ENSMUSG00000021276	Cinp	-1.000	2.92E-04	1.05E-02
ENSMUSG00000022663	Atg3	-0.998	4.58E-04	1.43E-02
ENSMUSG00000039067	Psmd7	-0.996	2.52E-03	4.79E-02
ENSMUSG00000030265	Kras	-0.996	3.38E-05	2.24E-03
ENSMUSG00000030521	Mphosph10	-0.992	2.51E-03	4.79E-02
ENSMUSG00000032026	Rexo2	-0.989	1.62E-03	3.54E-02
ENSMUSG00000019951	Uhrf1bp1l	-0.989	2.32E-04	8.85E-03

ENSMUSG00000032557	Uba5	-0.982	1.68E-03	3.59E-02
ENSMUSG00000038671	Arfrp1	-0.981	2.45E-03	4.70E-02
ENSMUSG00000026036	Nif3l1	-0.975	1.37E-04	6.05E-03
ENSMUSG00000057113	Npm1	-0.973	1.02E-04	5.07E-03
ENSMUSG00000030942	Thumpd1	-0.970	1.92E-04	7.79E-03
ENSMUSG00000030662	Ipo5	-0.969	2.90E-05	2.00E-03
ENSMUSG00000111877	Gm6477	-0.958	1.75E-03	3.68E-02
ENSMUSG00000028869	Gnl2	-0.958	1.05E-03	2.64E-02
ENSMUSG00000021692	Dimt1	-0.956	2.02E-03	4.08E-02
ENSMUSG00000022538	Lsg1	-0.948	2.53E-03	4.80E-02
ENSMUSG00000021235	Coq6	-0.948	7.96E-04	2.14E-02
ENSMUSG00000021737	Psmd6	-0.946	7.38E-07	1.30E-04
ENSMUSG00000017999	Ddx27	-0.941	4.58E-04	1.43E-02
ENSMUSG00000027357	Crls1	-0.941	2.66E-05	1.88E-03
ENSMUSG00000024436	Mrps18b	-0.939	1.19E-03	2.85E-02
ENSMUSG00000020224	Llph	-0.936	7.53E-04	2.06E-02
ENSMUSG00000018293	Pfn1	-0.936	2.44E-03	4.68E-02
ENSMUSG00000024712	Rfk	-0.934	2.25E-04	8.70E-03
ENSMUSG00000041028	Ghitm	-0.929	1.06E-03	2.65E-02
ENSMUSG00000043445	Pgp	-0.927	7.08E-04	1.99E-02
ENSMUSG00000031671	Setd6	-0.926	7.03E-04	1.98E-02
ENSMUSG00000027671	Actl6a	-0.920	7.23E-04	2.01E-02
ENSMUSG00000061613	U2af1	-0.919	1.09E-03	2.70E-02
ENSMUSG00000020464	Pnpt1	-0.918	1.63E-03	3.56E-02
ENSMUSG00000025439	Clns1a	-0.909	1.02E-03	2.59E-02
ENSMUSG00000020088	Sar1a	-0.908	1.31E-03	3.08E-02
ENSMUSG00000026377	Nifk	-0.907	1.10E-03	2.72E-02
ENSMUSG00000020248	Nfyb	-0.906	1.04E-04	5.13E-03
ENSMUSG00000019782	Rwdd1	-0.904	8.41E-04	2.24E-02
ENSMUSG00000036430	Tbcc	-0.901	1.74E-03	3.68E-02
ENSMUSG00000025393	Atp5b	-0.897	1.38E-03	3.18E-02
ENSMUSG00000022035	Ccdc25	-0.897	3.91E-04	1.30E-02
ENSMUSG00000053907	Mat2a	-0.896	1.04E-07	2.72E-05
ENSMUSG00000028907	Utp11	-0.886	1.48E-04	6.43E-03
ENSMUSG00000030224	Strap	-0.880	2.06E-03	4.14E-02
ENSMUSG00000020922	Lsm12	-0.878	2.75E-04	1.01E-02
ENSMUSG00000040681	Hmgn1	-0.877	1.38E-03	3.18E-02
ENSMUSG00000019795	Pcmt1	-0.876	3.82E-06	4.24E-04
ENSMUSG00000045503	Sys1	-0.871	1.23E-04	5.62E-03
ENSMUSG00000017428	Psmd11	-0.860	1.73E-03	3.67E-02
ENSMUSG00000001774	Chordc1	-0.860	3.96E-05	2.52E-03
ENSMUSG00000017801	Mlx	-0.859	1.88E-03	3.87E-02
ENSMUSG00000003438	Timm50	-0.856	1.24E-03	2.95E-02
ENSMUSG00000020720	Psmd12	-0.853	7.71E-04	2.09E-02
ENSMUSG00000015120	Ube2i	-0.845	1.42E-05	1.13E-03
ENSMUSG00000061207	Stk19	-0.844	1.85E-03	3.83E-02
ENSMUSG00000107470	Gm3375	-0.841	1.02E-03	2.58E-02

ENSMUSG00000026869	Psmd5	-0.840	4.42E-04	1.40E-02
ENSMUSG00000029551	Psmg3	-0.840	1.31E-04	5.82E-03
ENSMUSG00000028187	Rpf1	-0.838	1.15E-03	2.80E-02
ENSMUSG00000036371	Serbp1	-0.834	1.13E-04	5.40E-03
ENSMUSG00000030879	Mrpl17	-0.832	1.78E-04	7.39E-03
ENSMUSG00000028322	Exosc3	-0.831	4.27E-04	1.37E-02
ENSMUSG00000040521	Tsfm	-0.831	1.55E-03	3.44E-02
ENSMUSG00000039886	Tmem120a	-0.830	3.33E-04	1.15E-02
ENSMUSG00000010554	Mettl16	-0.828	1.03E-03	2.60E-02
ENSMUSG00000004268	Emg1	-0.826	3.08E-04	1.09E-02
ENSMUSG00000030603	Psmc4	-0.821	1.20E-03	2.88E-02
ENSMUSG00000038482	Tfdp1	-0.818	9.74E-04	2.50E-02
ENSMUSG00000026159	Agfg1	-0.816	8.71E-04	2.30E-02
ENSMUSG00000037740	Mrps26	-0.816	1.97E-04	7.95E-03
ENSMUSG00000021660	Btf3	-0.815	1.69E-03	3.61E-02
ENSMUSG00000018565	Elp5	-0.808	4.55E-06	4.86E-04
ENSMUSG00000027601	Mtfr1	-0.806	2.84E-04	1.03E-02
ENSMUSG00000021131	Erh	-0.797	1.04E-03	2.62E-02
ENSMUSG00000029922	Mkrn1	-0.795	3.19E-04	1.11E-02
ENSMUSG00000020180	Snrpd3	-0.793	2.11E-04	8.25E-03
ENSMUSG00000030512	Snrpa1	-0.788	2.32E-03	4.51E-02
ENSMUSG00000024580	Grpel2	-0.785	4.27E-05	2.68E-03
ENSMUSG00000028988	Ctnnbip1	-0.783	1.17E-03	2.83E-02
ENSMUSG00000020775	Mrpl38	-0.780	2.04E-04	8.11E-03
ENSMUSG00000051518	Rps19bp1	-0.779	1.36E-03	3.16E-02
ENSMUSG00000035021	Baz1a	-0.779	7.39E-04	2.04E-02
ENSMUSG00000030880	Polr3e	-0.777	1.35E-03	3.14E-02
ENSMUSG00000014195	Dnajc7	-0.760	9.00E-04	2.36E-02
ENSMUSG00000070697	Utp3	-0.756	9.93E-04	2.53E-02
ENSMUSG00000023147	Wrb	-0.755	2.29E-03	4.47E-02
ENSMUSG00000021578	Ccdc127	-0.754	1.44E-03	3.27E-02
ENSMUSG00000032002	Dcun1d5	-0.751	8.52E-05	4.52E-03
ENSMUSG00000078652	Psme3	-0.738	2.11E-03	4.21E-02
ENSMUSG00000022204	Ngdn	-0.737	1.75E-05	1.36E-03
ENSMUSG00000040843	Tiprl	-0.736	1.68E-03	3.59E-02
ENSMUSG00000034620	Tmem5	-0.720	1.90E-03	3.89E-02
ENSMUSG00000032563	Mrpl3	-0.691	2.45E-04	9.29E-03
ENSMUSG00000079478	Sssca1	-0.684	2.01E-03	4.06E-02
ENSMUSG00000024878	Cbwd1	-0.682	1.25E-03	2.96E-02
ENSMUSG00000015290	Ubl4a	-0.677	1.64E-03	3.57E-02
ENSMUSG00000021018	Polr2h	-0.674	2.13E-03	4.24E-02
ENSMUSG00000039640	Mrpl12	-0.674	2.72E-04	1.00E-02
ENSMUSG00000055762	Eef1d	-0.667	1.90E-03	3.89E-02
ENSMUSG00000029038	Ssu72	-0.657	4.52E-04	1.42E-02
ENSMUSG00000022858	Tra2b	-0.654	2.22E-03	4.38E-02
ENSMUSG00000001416	Cct3	-0.651	2.02E-03	4.08E-02
ENSMUSG00000031697	Orc6	-0.623	2.10E-03	4.20E-02

ENSMUSG00000043284	Tmem11	-0.622	7.94E-04	2.14E-02
ENSMUSG00000061360	Phf5a	-0.609	4.81E-04	1.49E-02
ENSMUSG00000034024	Cct2	-0.606	9.05E-05	4.65E-03
ENSMUSG00000043866	Taf10	-0.603	1.28E-03	3.01E-02
ENSMUSG00000022427	Tomm22	-0.371	2.60E-03	4.90E-02
ENSMUSG00000022671	Mzt2	0.577	2.13E-03	4.24E-02
ENSMUSG00000025102	3110040N11	0.603	1.46E-03	3.31E-02
ENSMUSG00000002345	Borcs8	0.644	2.28E-03	4.47E-02
ENSMUSG00000027104	Atf2	0.708	9.52E-05	4.82E-03
ENSMUSG00000031360	Ctps2	0.729	6.15E-05	3.57E-03
ENSMUSG00000031023	Akip1	0.736	1.24E-04	5.63E-03
ENSMUSG00000037608	Bclaf1	0.742	6.06E-04	1.77E-02
ENSMUSG00000025040	Fundc1	0.746	2.58E-03	4.87E-02
ENSMUSG00000029433	Diablo	0.747	6.74E-05	3.78E-03
ENSMUSG00000009030	Pdcl	0.763	9.64E-05	4.84E-03
ENSMUSG00000022051	Bnip3l	0.767	2.60E-03	4.89E-02
ENSMUSG00000051285	Pcmtd1	0.799	1.92E-03	3.91E-02
ENSMUSG00000066724	Gm10175	0.802	1.17E-03	2.83E-02
ENSMUSG00000028070	Naxe	0.805	1.15E-03	2.80E-02
ENSMUSG00000052459	Atp6v1a	0.810	2.52E-03	4.79E-02
ENSMUSG00000056211	R3hdm1	0.828	5.28E-04	1.60E-02
ENSMUSG00000058325	Dock1	0.842	8.01E-04	2.15E-02
ENSMUSG00000025505	Tmem80	0.851	2.35E-03	4.57E-02
ENSMUSG00000039159	Ube2h	0.857	1.08E-03	2.70E-02
ENSMUSG00000001018	Snapin	0.861	1.05E-03	2.64E-02
ENSMUSG00000025035	Arl3	0.862	6.94E-04	1.96E-02
ENSMUSG00000027709	Mccc1	0.864	1.79E-03	3.74E-02
ENSMUSG00000028062	Lamtor2	0.866	1.25E-03	2.96E-02
ENSMUSG00000053929	Cyhr1	0.879	3.27E-05	2.18E-03
ENSMUSG00000030613	Ccdc90b	0.879	3.14E-04	1.11E-02
ENSMUSG00000034951	Cog7	0.888	7.97E-04	2.14E-02
ENSMUSG00000033596	Rfwd3	0.900	1.39E-03	3.19E-02
ENSMUSG00000024038	Ndufv3	0.905	6.32E-04	1.82E-02
ENSMUSG00000022092	Ppp3cc	0.912	1.58E-03	3.49E-02
ENSMUSG00000050552	Lamtor4	0.924	9.28E-04	2.42E-02
ENSMUSG00000032398	Snapc5	0.932	1.25E-05	1.02E-03
ENSMUSG00000021109	Hif1a	0.935	1.09E-04	5.28E-03
ENSMUSG00000034744	Nagk	0.941	1.92E-03	3.91E-02
ENSMUSG00000020091	Eif4ebp2	0.944	3.15E-04	1.11E-02
ENSMUSG00000030515	Tarsl2	0.962	7.93E-04	2.14E-02
ENSMUSG00000021948	Prkcd	0.967	1.64E-03	3.58E-02
ENSMUSG00000030213	Atf7ip	0.970	3.52E-04	1.21E-02
ENSMUSG00000079427	Mthfsl	0.982	2.22E-04	8.62E-03
ENSMUSG00000039795	Zfand1	0.987	1.68E-03	3.59E-02
ENSMUSG00000028580	Pum1	0.987	2.36E-03	4.58E-02
ENSMUSG00000036880	Acaa2	0.999	2.59E-03	4.89E-02
ENSMUSG00000044201	Cdc25c	1.002	1.66E-03	3.58E-02

ENSMUSG00000022617	Chkb	1.002	6.75E-04	1.92E-02
ENSMUSG00000040128	Pnrc1	1.011	6.86E-04	1.94E-02
ENSMUSG00000078566	Bnip3	1.033	7.59E-04	2.06E-02
ENSMUSG00000025860	Xiap	1.036	1.63E-03	3.56E-02
ENSMUSG00000035637	Grhpr	1.039	6.09E-04	1.77E-02
ENSMUSG00000057367	Birc2	1.048	5.00E-04	1.53E-02
ENSMUSG00000073198	Bnip3l-ps	1.050	1.52E-03	3.38E-02
ENSMUSG00000057572	Zbtb8os	1.055	1.40E-03	3.22E-02
ENSMUSG00000023966	Rsph9	1.058	1.82E-04	7.52E-03
ENSMUSG00000029401	Rilpl2	1.065	4.18E-04	1.35E-02
ENSMUSG00000006021	Kptn	1.067	8.28E-04	2.21E-02
ENSMUSG00000040795	Iqcc	1.069	2.02E-03	4.08E-02
ENSMUSG00000035901	Dennd5a	1.078	4.44E-04	1.40E-02
ENSMUSG00000042364	Snx18	1.084	1.17E-04	5.48E-03
ENSMUSG00000026721	Rabgap1l	1.090	1.15E-03	2.80E-02
ENSMUSG00000002580	Mien1	1.115	3.85E-05	2.46E-03
ENSMUSG00000033111	3830406C13	1.115	6.80E-06	6.61E-04
ENSMUSG00000036644	Tbc1d9b	1.116	2.38E-03	4.62E-02
ENSMUSG00000031641	Cbr4	1.120	3.73E-05	2.42E-03
ENSMUSG00000042408	Zmym6	1.124	9.56E-04	2.47E-02
ENSMUSG00000028382	Ptbp3	1.125	3.56E-04	1.21E-02
ENSMUSG00000040549	Ckap5	1.139	4.58E-04	1.43E-02
ENSMUSG00000021643	Serf1	1.148	2.30E-03	4.48E-02
ENSMUSG00000054894	Atp5s	1.148	1.60E-04	6.85E-03
ENSMUSG00000000486	45170	1.149	1.46E-03	3.31E-02
ENSMUSG00000042055	Wdr11	1.164	1.99E-03	4.04E-02
ENSMUSG00000037847	Nmrk1	1.166	2.17E-03	4.29E-02
ENSMUSG00000031529	Tnks	1.167	2.52E-03	4.79E-02
ENSMUSG00000021033	Gstz1	1.181	6.22E-06	6.19E-04
ENSMUSG00000029471	Camkk2	1.191	9.81E-04	2.51E-02
ENSMUSG00000026154	Sdhaf4	1.194	1.69E-03	3.61E-02
ENSMUSG00000037152	Ndufc1	1.196	1.28E-03	3.01E-02
ENSMUSG00000032038	St3gal4	1.198	1.74E-04	7.31E-03
ENSMUSG00000074749	Kiz	1.203	2.31E-04	8.82E-03
ENSMUSG00000035142	Nubpl	1.204	6.17E-04	1.79E-02
ENSMUSG00000026385	Dbi	1.206	2.45E-03	4.70E-02
ENSMUSG00000027984	Hadh	1.207	3.16E-05	2.12E-03
ENSMUSG00000037628	Cdkn3	1.219	2.63E-03	4.95E-02
ENSMUSG00000039745	Htatip2	1.225	1.06E-03	2.66E-02
ENSMUSG00000028101	Pias3	1.231	1.25E-03	2.97E-02
ENSMUSG00000060147	Serpinb6a	1.234	1.49E-04	6.43E-03
ENSMUSG00000006717	Acot13	1.240	3.35E-06	3.97E-04
ENSMUSG00000024392	Bag6	1.242	6.00E-04	1.76E-02
ENSMUSG00000042506	Usp22	1.244	7.80E-04	2.11E-02
ENSMUSG00000022817	Itgb5	1.245	3.16E-04	1.11E-02
ENSMUSG00000042675	Ypel3	1.249	1.90E-05	1.43E-03
ENSMUSG00000038014	Fam120a	1.257	1.06E-04	5.21E-03

ENSMUSG00000025993	Slc40a1	1.261	8.67E-05	4.58E-03
ENSMUSG00000031161	Hdac6	1.262	1.67E-03	3.59E-02
ENSMUSG00000065979	Cpped1	1.264	5.80E-04	1.72E-02
ENSMUSG00000037499	Nenf	1.265	1.47E-03	3.31E-02
ENSMUSG00000040841	Six5	1.267	2.23E-05	1.62E-03
ENSMUSG00000030779	Rbbp6	1.272	9.71E-04	2.50E-02
ENSMUSG00000042155	Klhl23	1.274	3.99E-04	1.31E-02
ENSMUSG00000014075	Tctex1d2	1.278	5.80E-05	3.42E-03
ENSMUSG00000027165	B230118H07	1.287	1.32E-04	5.87E-03
ENSMUSG00000008822	Acyp1	1.287	4.16E-04	1.35E-02
ENSMUSG00000042293	Gm5617	1.288	2.55E-04	9.57E-03
ENSMUSG00000025731	Mettl26	1.289	2.39E-03	4.63E-02
ENSMUSG00000023911	Flywch2	1.294	1.91E-03	3.90E-02
ENSMUSG00000028549	Itgb3bp	1.296	4.73E-04	1.46E-02
ENSMUSG00000040370	Etfrf1	1.297	3.53E-04	1.21E-02
ENSMUSG00000038718	Pbx3	1.299	2.69E-04	9.95E-03
ENSMUSG00000022773	Ypel1	1.299	1.88E-03	3.87E-02
ENSMUSG00000008035	Mid1ip1	1.303	1.30E-03	3.06E-02
ENSMUSG00000028470	Hint2	1.303	1.24E-04	5.63E-03
ENSMUSG00000070394	Tmem256	1.310	1.33E-03	3.10E-02
ENSMUSG00000033429	Mcee	1.311	4.54E-05	2.80E-03
ENSMUSG00000023094	Msrb2	1.313	5.16E-04	1.57E-02
ENSMUSG00000041126	H2afv	1.314	9.22E-06	8.27E-04
ENSMUSG00000078695	Cisd3	1.320	4.67E-04	1.45E-02
ENSMUSG00000072772	Grcc10	1.320	3.71E-05	2.42E-03
ENSMUSG00000024960	Plcb3	1.321	1.57E-04	6.75E-03
ENSMUSG00000057103	Nat8f1	1.323	7.33E-04	2.03E-02
ENSMUSG00000044475	Ascc1	1.328	1.98E-04	7.95E-03
ENSMUSG00000022708	Zbtb20	1.345	2.77E-04	1.01E-02
ENSMUSG00000052102	Gnpda1	1.347	8.91E-05	4.64E-03
ENSMUSG00000097867	Lppos	1.352	1.46E-03	3.31E-02
ENSMUSG00000073633	Fbxo36	1.366	4.42E-04	1.40E-02
ENSMUSG00000032216	Nedd4	1.366	7.46E-05	4.08E-03
ENSMUSG00000028756	Pink1	1.367	2.03E-03	4.09E-02
ENSMUSG00000032702	Kank1	1.373	1.16E-03	2.82E-02
ENSMUSG00000029521	Chek2	1.381	1.94E-03	3.94E-02
ENSMUSG00000036782	Klhl13	1.385	1.58E-03	3.49E-02
ENSMUSG00000045867	Cradd	1.391	1.33E-04	5.91E-03
ENSMUSG00000027654	Fam83d	1.391	6.53E-04	1.87E-02
ENSMUSG00000022550	Adck5	1.394	6.07E-04	1.77E-02
ENSMUSG00000027187	Cat	1.403	7.86E-05	4.21E-03
ENSMUSG00000052139	Babam2	1.407	9.80E-06	8.61E-04
ENSMUSG00000028795	Ccdc28b	1.408	3.21E-08	1.11E-05
ENSMUSG00000020150	Gamt	1.411	8.04E-04	2.16E-02
ENSMUSG00000002058	Unc119	1.419	3.73E-05	2.42E-03
ENSMUSG00000049225	Pdp1	1.419	1.26E-08	5.17E-06
ENSMUSG00000002043	Trappc6a	1.420	1.18E-04	5.50E-03

ENSMUSG00000097059	Fam120aos	1.427	1.26E-03	2.98E-02
ENSMUSG00000001666	Ddt	1.428	1.02E-03	2.59E-02
ENSMUSG00000030990	Pgap2	1.431	3.53E-06	4.11E-04
ENSMUSG00000046982	Tshz1	1.434	1.56E-03	3.45E-02
ENSMUSG00000035642	Aamdc	1.436	6.22E-05	3.57E-03
ENSMUSG00000019689	Fmc1	1.438	3.07E-05	2.07E-03
ENSMUSG00000020898	Ctc1	1.443	4.13E-04	1.35E-02
ENSMUSG00000034610	Zcchc11	1.454	2.00E-04	8.04E-03
ENSMUSG00000033685	Ucp2	1.459	3.36E-05	2.23E-03
ENSMUSG00000044252	Osbpl1a	1.461	2.53E-06	3.39E-04
ENSMUSG00000021792	Fam213a	1.465	1.47E-03	3.31E-02
ENSMUSG00000029385	Ccng2	1.468	6.29E-06	6.22E-04
ENSMUSG00000064215	Ifi27	1.485	9.16E-06	8.27E-04
ENSMUSG00000033065	Pfkm	1.506	1.43E-03	3.26E-02
ENSMUSG00000027332	Ivd	1.509	1.12E-05	9.42E-04
ENSMUSG00000037253	Mex3c	1.516	2.64E-04	9.88E-03
ENSMUSG00000010362	Rdm1	1.521	2.57E-05	1.84E-03
ENSMUSG00000060261	Gtf2i	1.537	1.91E-04	7.79E-03
ENSMUSG00000039286	Fndc3b	1.556	1.60E-03	3.52E-02
ENSMUSG00000032220	Myo1e	1.565	8.97E-04	2.36E-02
ENSMUSG00000035048	Anapc13	1.567	7.84E-05	4.21E-03
ENSMUSG00000058756	Thra	1.567	9.65E-04	2.48E-02
ENSMUSG00000020601	Trib2	1.572	8.02E-06	7.56E-04
ENSMUSG00000050538	B230217C12	1.573	2.06E-03	4.13E-02
ENSMUSG00000045257	Morn2	1.573	2.36E-04	9.01E-03
ENSMUSG00000037493	Cib2	1.579	4.64E-04	1.45E-02
ENSMUSG00000034449	Dhrs11	1.583	1.78E-04	7.39E-03
ENSMUSG00000022040	Ephx2	1.593	1.53E-03	3.40E-02
ENSMUSG00000025955	Akr1cl	1.593	6.20E-05	3.57E-03
ENSMUSG00000023055	Calcoco1	1.595	5.55E-05	3.30E-03
ENSMUSG00000023150	Ivns1abp	1.601	6.07E-04	1.77E-02
ENSMUSG00000024866	Acy3	1.610	2.09E-04	8.20E-03
ENSMUSG00000036879	Phkb	1.610	6.50E-04	1.87E-02
ENSMUSG00000066456	Hmgn3	1.611	1.29E-05	1.05E-03
ENSMUSG00000021065	Fut8	1.611	6.86E-04	1.94E-02
ENSMUSG00000048490	Nrip1	1.623	1.87E-03	3.86E-02
ENSMUSG00000097415	AU020206	1.624	1.61E-04	6.85E-03
ENSMUSG00000022048	Dpysl2	1.633	7.81E-04	2.11E-02
ENSMUSG00000030122	Ptms	1.633	2.26E-04	8.70E-03
ENSMUSG00000038286	Bphl	1.639	3.14E-06	3.94E-04
ENSMUSG00000031666	Rbl2	1.641	4.03E-04	1.32E-02
ENSMUSG00000038070	Cntln	1.648	5.49E-04	1.64E-02
ENSMUSG00000018417	Myo1b	1.656	3.84E-04	1.28E-02
ENSMUSG00000034522	Zfp395	1.667	4.62E-06	4.88E-04
ENSMUSG00000021775	Nr1d2	1.670	1.34E-06	2.08E-04
ENSMUSG00000022587	Ly6e	1.680	3.80E-04	1.27E-02
ENSMUSG00000039450	Dcxr	1.697	1.05E-06	1.70E-04

ENSMUSG00000032067	Pts	1.698	2.79E-08	9.81E-06
ENSMUSG00000022323	Rida	1.699	4.45E-05	2.77E-03
ENSMUSG00000046718	Bst2	1.703	1.77E-03	3.72E-02
ENSMUSG00000027589	Pcmtd2	1.710	4.18E-06	4.56E-04
ENSMUSG00000100975	Gm28875	1.723	1.00E-03	2.55E-02
ENSMUSG00000086158	Ccpg1os	1.730	4.38E-05	2.74E-03
ENSMUSG00000026211	Obsl1	1.737	1.76E-06	2.55E-04
ENSMUSG00000026495	Efcab2	1.737	9.22E-05	4.71E-03
ENSMUSG00000087165	2010001A14	1.739	8.55E-04	2.27E-02
ENSMUSG00000026839	Upp2	1.746	2.68E-04	9.93E-03
ENSMUSG00000041390	Mdfic	1.749	2.30E-03	4.48E-02
ENSMUSG00000047003	Zfp41	1.753	2.16E-04	8.42E-03
ENSMUSG00000073755	5730409E04	1.753	1.13E-03	2.77E-02
ENSMUSG00000025404	R3hdm2	1.760	1.69E-05	1.33E-03
ENSMUSG00000051817	Sox12	1.766	2.07E-06	2.88E-04
ENSMUSG00000032609	Klhdc8b	1.781	1.83E-03	3.80E-02
ENSMUSG00000097162	2310010J17I	1.789	1.21E-04	5.58E-03
ENSMUSG00000032221	Mns1	1.800	1.66E-03	3.58E-02
ENSMUSG00000028108	Ecm1	1.800	1.26E-04	5.66E-03
ENSMUSG00000054723	Vmac	1.804	2.57E-05	1.84E-03
ENSMUSG00000086468	Etaa1os	1.811	1.16E-04	5.48E-03
ENSMUSG00000025466	Fuom	1.816	5.48E-06	5.66E-04
ENSMUSG00000046329	Slc25a23	1.822	3.80E-04	1.27E-02
ENSMUSG00000043644	0610009L18	1.824	3.17E-04	1.11E-02
ENSMUSG00000040249	Lrp1	1.826	7.22E-04	2.01E-02
ENSMUSG00000051343	Rab11fip5	1.828	2.88E-04	1.04E-02
ENSMUSG00000010307	Tmem86a	1.837	5.82E-04	1.72E-02
ENSMUSG00000079598	Clec2l	1.838	1.58E-03	3.49E-02
ENSMUSG00000021238	Aldh6a1	1.844	5.19E-04	1.57E-02
ENSMUSG00000026664	Phyh	1.844	6.39E-08	1.82E-05
ENSMUSG00000107369	Gstm2-ps1	1.853	1.22E-05	1.00E-03
ENSMUSG00000091561	Gm6665	1.854	9.23E-05	4.71E-03
ENSMUSG00000024253	Dync2li1	1.861	1.65E-09	9.65E-07
ENSMUSG00000034101	Ctnnd1	1.863	1.50E-03	3.36E-02
ENSMUSG00000087336	Gm15860	1.883	9.75E-04	2.50E-02
ENSMUSG00000039958	Etfbkmt	1.889	4.21E-04	1.36E-02
ENSMUSG00000048581	E130311K13	1.904	4.25E-06	4.61E-04
ENSMUSG00000021690	Jmy	1.914	3.85E-05	2.46E-03
ENSMUSG00000022661	Cd200	1.927	1.44E-05	1.15E-03
ENSMUSG00000031453	Rasa3	1.935	6.48E-06	6.33E-04
ENSMUSG00000004633	Chn2	1.936	1.87E-03	3.86E-02
ENSMUSG00000086587	Gm11837	1.945	1.08E-04	5.26E-03
ENSMUSG00000000594	Gm2a	1.948	2.48E-03	4.73E-02
ENSMUSG00000033577	Myo6	1.954	1.49E-05	1.18E-03
ENSMUSG00000044080	S100a1	1.962	1.62E-06	2.44E-04
ENSMUSG00000058135	Gstm1	1.966	2.99E-06	3.83E-04
ENSMUSG00000040562	Gstm2	1.974	4.74E-05	2.88E-03

ENSMUSG00000053279	Aldh1a1	1.977	2.64E-05	1.88E-03
ENSMUSG00000044986	Tst	1.982	1.25E-04	5.63E-03
ENSMUSG00000032009	Sesn3	1.988	6.02E-06	6.06E-04
ENSMUSG00000060675	Pla2g16	1.989	4.64E-05	2.84E-03
ENSMUSG00000036894	Rap2b	2.000	1.50E-03	3.36E-02
ENSMUSG00000067336	Bmpr2	2.014	4.57E-04	1.43E-02
ENSMUSG00000037606	Osbpl5	2.049	4.16E-04	1.35E-02
ENSMUSG00000003031	Cdkn1b	2.050	1.18E-05	9.74E-04
ENSMUSG00000024646	Cyb5a	2.050	6.19E-05	3.57E-03
ENSMUSG00000049086	Bmyc	2.057	2.88E-04	1.04E-02
ENSMUSG00000062866	Phactr2	2.058	1.85E-07	4.07E-05
ENSMUSG00000027935	Rab13	2.059	1.33E-05	1.07E-03
ENSMUSG00000042712	Tceal9	2.061	7.20E-07	1.28E-04
ENSMUSG00000020216	Jsrp1	2.063	2.68E-04	9.93E-03
ENSMUSG00000043079	Synpo	2.068	1.83E-03	3.80E-02
ENSMUSG00000021619	Atg10	2.082	6.65E-05	3.75E-03
ENSMUSG00000022390	Zc3h7b	2.100	4.52E-05	2.79E-03
ENSMUSG00000019718	L3hypdh	2.111	4.27E-06	4.61E-04
ENSMUSG00000023191	P3h3	2.114	5.89E-04	1.74E-02
ENSMUSG00000013033	Adgrl1	2.115	7.45E-04	2.05E-02
ENSMUSG00000036053	Fmnl2	2.115	1.46E-03	3.31E-02
ENSMUSG00000021094	Dhrs7	2.134	2.40E-03	4.65E-02
ENSMUSG00000098318	Lockd	2.140	1.04E-04	5.13E-03
ENSMUSG00000031099	Smarca1	2.141	2.61E-07	5.49E-05
ENSMUSG00000026365	Cfh	2.163	2.22E-03	4.38E-02
ENSMUSG00000011382	Dhdh	2.168	1.13E-03	2.77E-02
ENSMUSG00000111080	Gm20300	2.173	4.79E-05	2.90E-03
ENSMUSG00000073985	Gm10602	2.177	3.85E-04	1.28E-02
ENSMUSG00000030630	Fah	2.193	2.70E-05	1.90E-03
ENSMUSG00000066357	Wdr6	2.195	2.75E-10	1.97E-07
ENSMUSG00000067878	Map7d3	2.196	8.96E-04	2.36E-02
ENSMUSG00000031760	Mt3	2.205	6.72E-04	1.92E-02
ENSMUSG00000089768	Tmsb15b1	2.209	8.99E-04	2.36E-02
ENSMUSG00000027223	Mapk8ip1	2.216	2.42E-03	4.66E-02
ENSMUSG00000059645	Gm7361	2.220	3.61E-04	1.22E-02
ENSMUSG00000112876	Gm32443	2.234	2.03E-04	8.10E-03
ENSMUSG00000031613	Hpgd	2.246	5.75E-04	1.71E-02
ENSMUSG00000030329	Pianp	2.247	2.43E-03	4.67E-02
ENSMUSG00000040557	Mettl27	2.267	2.41E-10	1.79E-07
ENSMUSG00000037826	Ppm1k	2.273	1.32E-03	3.08E-02
ENSMUSG00000033910	Gucy1a1	2.307	8.74E-04	2.30E-02
ENSMUSG00000024713	Pcsk5	2.308	2.52E-04	9.48E-03
ENSMUSG00000026817	Ak1	2.310	1.83E-03	3.80E-02
ENSMUSG00000002409	Dyrk1b	2.318	1.49E-03	3.34E-02
ENSMUSG00000030055	Rab43	2.322	8.66E-04	2.29E-02
ENSMUSG00000096727	Psmb9	2.323	1.13E-03	2.77E-02
ENSMUSG00000027848	Olfml3	2.327	5.39E-04	1.62E-02

ENSMUSG00000027227	Sord	2.365	2.07E-04	8.19E-03
ENSMUSG00000034371	Tkfc	2.367	1.61E-04	6.85E-03
ENSMUSG00000020486	45173	2.373	1.42E-04	6.22E-03
ENSMUSG00000085614	1700123M08	2.377	8.23E-05	4.38E-03
ENSMUSG00000062901	Klhl24	2.377	8.57E-09	3.77E-06
ENSMUSG00000051515	Fam181b	2.397	6.80E-05	3.78E-03
ENSMUSG00000027875	Hmgcs2	2.403	5.50E-04	1.64E-02
ENSMUSG00000038248	Sobp	2.425	5.58E-06	5.74E-04
ENSMUSG00000051098	Mblac2	2.428	2.41E-03	4.65E-02
ENSMUSG00000020962	Gtf2a1	2.441	1.24E-04	5.63E-03
ENSMUSG00000033488	Cryzl2	2.442	2.10E-08	8.13E-06
ENSMUSG00000039765	Cc2d2a	2.445	7.43E-04	2.04E-02
ENSMUSG00000020263	Appl2	2.454	5.24E-07	9.93E-05
ENSMUSG00000061535	C1qtnf7	2.468	1.77E-04	7.38E-03
ENSMUSG00000020388	Pdlim4	2.506	1.36E-07	3.36E-05
ENSMUSG00000116656	AC131339.2	2.536	2.08E-04	8.20E-03
ENSMUSG00000038543	BC028528	2.539	3.95E-04	1.31E-02
ENSMUSG00000055435	Maf	2.543	1.18E-03	2.84E-02
ENSMUSG00000031284	Pak3	2.558	2.22E-03	4.38E-02
ENSMUSG00000025875	Tspan17	2.565	1.54E-04	6.62E-03
ENSMUSG00000028015	Ctso	2.571	9.22E-04	2.41E-02
ENSMUSG00000024598	Fbn2	2.578	1.83E-03	3.80E-02
ENSMUSG00000030793	Pycard	2.587	6.26E-05	3.58E-03
ENSMUSG00000031310	Zmym3	2.598	7.04E-06	6.81E-04
ENSMUSG00000099034	2810039B14	2.604	3.04E-04	1.08E-02
ENSMUSG00000110935	Gm8834	2.606	8.64E-05	4.58E-03
ENSMUSG00000001520	Nrip2	2.619	1.48E-04	6.43E-03
ENSMUSG00000070315	4930581F22	2.623	1.62E-03	3.55E-02
ENSMUSG00000002997	Prkar2b	2.654	1.10E-07	2.81E-05
ENSMUSG00000042109	Csdc2	2.658	2.20E-05	1.62E-03
ENSMUSG00000021884	Hacl1	2.688	2.69E-08	9.78E-06
ENSMUSG00000028773	Fabp3	2.697	1.90E-04	7.77E-03
ENSMUSG00000041132	N4bp2l1	2.788	1.03E-03	2.61E-02
ENSMUSG00000036545	Adamts2	2.790	5.94E-05	3.48E-03
ENSMUSG00000022440	C1qtnf6	2.791	1.97E-06	2.80E-04
ENSMUSG00000087380	2210408F21	2.793	2.72E-05	1.90E-03
ENSMUSG00000089996	Tmsb15b2	2.795	1.52E-04	6.54E-03
ENSMUSG00000078350	Smim1	2.808	1.16E-04	5.47E-03
ENSMUSG00000001313	Rnd2	2.812	1.95E-04	7.91E-03
ENSMUSG00000116097	AL590144.2	2.820	6.79E-05	3.78E-03
ENSMUSG00000052957	Gas1	2.838	2.65E-03	4.96E-02
ENSMUSG00000033420	Antxr1	2.843	1.18E-04	5.50E-03
ENSMUSG00000022389	Tef	2.873	5.18E-07	9.92E-05
ENSMUSG00000040666	Sh3bgr	2.876	1.80E-03	3.76E-02
ENSMUSG00000023982	Guca1a	2.885	1.15E-04	5.47E-03
ENSMUSG00000002346	Slc25a42	2.896	1.88E-03	3.87E-02
ENSMUSG00000101655	2310040G24	2.900	6.63E-04	1.89E-02

ENSMUSG00000029838	Ptn	2.902	2.56E-03	4.85E-02
ENSMUSG00000085260	Med9os	2.925	6.14E-04	1.78E-02
ENSMUSG00000072966	Gprasp2	2.949	1.33E-03	3.10E-02
ENSMUSG00000033208	S100b	2.961	1.82E-03	3.80E-02
ENSMUSG00000030074	Gxylt2	2.985	9.82E-04	2.51E-02
ENSMUSG00000039533	Mmd2	2.986	7.15E-04	2.00E-02
ENSMUSG00000057948	Unc13d	2.988	4.55E-04	1.43E-02
ENSMUSG00000064342	mt-Ti	2.993	1.64E-03	3.57E-02
ENSMUSG00000112646	Gm5654	3.024	2.88E-04	1.04E-02
ENSMUSG00000059022	Kcp	3.026	1.64E-04	6.93E-03
ENSMUSG00000013083	2200002J24I	3.029	1.65E-03	3.58E-02
ENSMUSG00000060224	Pyroxd2	3.043	1.48E-03	3.32E-02
ENSMUSG00000055692	Tmem191c	3.070	1.14E-04	5.45E-03
ENSMUSG00000050953	Gja1	3.081	3.72E-04	1.25E-02
ENSMUSG00000086382	Chrna1os	3.095	7.48E-08	2.04E-05
ENSMUSG00000086900	Kcnab3os	3.103	4.26E-04	1.37E-02
ENSMUSG00000026489	Coq8a	3.114	2.39E-09	1.25E-06
ENSMUSG00000041476	Smpx	3.115	2.25E-08	8.36E-06
ENSMUSG00000020099	Unc5b	3.167	2.01E-04	8.04E-03
ENSMUSG00000086863	Gm15543	3.194	4.36E-04	1.38E-02
ENSMUSG00000022883	Robo1	3.217	8.92E-04	2.35E-02
ENSMUSG00000042500	Ago4	3.229	1.23E-04	5.62E-03
ENSMUSG00000074595	Wfdc6a	3.237	7.31E-04	2.03E-02
ENSMUSG00000085347	Hk1os	3.268	1.22E-05	1.00E-03
ENSMUSG00000055493	Epm2a	3.272	2.14E-03	4.24E-02
ENSMUSG00000027966	Col11a1	3.297	8.14E-07	1.39E-04
ENSMUSG00000024818	Slc25a45	3.319	7.23E-04	2.01E-02
ENSMUSG00000027217	Tspan18	3.326	2.01E-04	8.04E-03
ENSMUSG00000038776	Ephx1	3.338	1.44E-03	3.28E-02
ENSMUSG00000012819	Cdh23	3.352	1.39E-03	3.20E-02
ENSMUSG00000045731	Pnoc	3.359	2.18E-06	3.01E-04
ENSMUSG00000097354	2310001H17	3.388	5.53E-04	1.65E-02
ENSMUSG00000041536	Serpina3a	3.401	3.59E-04	1.22E-02
ENSMUSG00000033610	Pank1	3.405	2.77E-06	3.64E-04
ENSMUSG00000031877	Ces2g	3.412	1.78E-03	3.74E-02
ENSMUSG00000090925	1810064F22	3.426	1.85E-03	3.84E-02
ENSMUSG00000081359	Cox20-ps	3.426	1.06E-03	2.65E-02
ENSMUSG00000034810	Scn7a	3.432	1.47E-03	3.31E-02
ENSMUSG00000097413	A830052D11	3.444	1.47E-03	3.31E-02
ENSMUSG00000022235	Cmbl	3.450	9.50E-09	4.04E-06
ENSMUSG00000032224	Fam81a	3.458	5.07E-07	9.80E-05
ENSMUSG00000029163	Emilin1	3.469	4.97E-04	1.53E-02
ENSMUSG00000100005	B130024G19	3.475	4.36E-04	1.38E-02
ENSMUSG00000035561	Aldh1b1	3.478	1.75E-03	3.68E-02
ENSMUSG00000041000	Trim62	3.481	1.90E-09	1.08E-06
ENSMUSG00000033715	Akr1c14	3.483	4.08E-11	3.94E-08
ENSMUSG00000031871	Cdh5	3.488	1.25E-04	5.63E-03

ENSMUSG00000067279	Ppp1r3c	3.507	2.12E-03	4.22E-02
ENSMUSG00000107736	Gm44148	3.509	6.04E-04	1.77E-02
ENSMUSG00000050645	Defb19	3.517	2.31E-04	8.82E-03
ENSMUSG00000025934	Gsta3	3.559	7.80E-11	6.56E-08
ENSMUSG00000040111	Gramd1b	3.601	1.73E-05	1.35E-03
ENSMUSG00000021185	Dglucy	3.614	5.47E-05	3.27E-03
ENSMUSG00000026831	1700007K13	3.615	1.73E-03	3.67E-02
ENSMUSG00000079013	Serpina3j	3.659	1.66E-04	7.01E-03
ENSMUSG00000044835	Ankrd45	3.670	4.07E-04	1.33E-02
ENSMUSG00000025468	Caly	3.683	3.92E-04	1.30E-02
ENSMUSG00000030474	Siglece	3.684	2.57E-03	4.86E-02
ENSMUSG00000059857	Ntng1	3.689	2.41E-03	4.66E-02
ENSMUSG00000017453	Pipox	3.706	1.63E-03	3.56E-02
ENSMUSG00000045672	Col27a1	3.754	1.66E-03	3.58E-02
ENSMUSG00000027489	Necab3	3.756	7.42E-04	2.04E-02
ENSMUSG00000019301	Hsd17b1	3.761	6.43E-05	3.65E-03
ENSMUSG00000004892	Bcan	3.799	5.00E-04	1.53E-02
ENSMUSG00000030257	Srgap3	3.806	4.62E-05	2.84E-03
ENSMUSG00000046182	Gsg1l	3.817	1.28E-03	3.01E-02
ENSMUSG00000042129	Rassf4	3.825	2.67E-04	9.93E-03
ENSMUSG00000001663	Gstt1	3.833	2.12E-09	1.17E-06
ENSMUSG00000021294	Kif26a	3.834	1.29E-03	3.03E-02
ENSMUSG00000037161	Mgarp	3.847	1.96E-04	7.93E-03
ENSMUSG00000068854	Hist2h2be	3.849	1.21E-03	2.90E-02
ENSMUSG00000034059	Ypel4	3.849	9.56E-04	2.47E-02
ENSMUSG00000109080	Gm38944	3.939	1.22E-03	2.91E-02
ENSMUSG00000039676	Capsl	3.946	7.07E-04	1.99E-02
ENSMUSG00000109992	Gm45650	3.954	1.66E-03	3.58E-02
ENSMUSG00000074665	Bpifb4	3.961	6.16E-04	1.79E-02
ENSMUSG00000033082	Clec1a	3.965	2.40E-03	4.64E-02
ENSMUSG00000021806	Nid2	3.973	2.01E-05	1.50E-03
ENSMUSG00000001665	Gstt3	3.993	3.76E-08	1.25E-05
ENSMUSG00000028415	Spink4	3.994	7.49E-04	2.05E-02
ENSMUSG00000024620	Pdgfrb	4.013	1.46E-04	6.35E-03
ENSMUSG00000045392	Olfr1033	4.015	1.47E-03	3.31E-02
ENSMUSG00000034413	Neurl1b	4.022	8.53E-04	2.26E-02
ENSMUSG00000053821	Gm9922	4.027	1.71E-03	3.64E-02
ENSMUSG00000021213	Akr1c13	4.028	1.16E-04	5.48E-03
ENSMUSG00000032135	Mcam	4.049	7.83E-04	2.12E-02
ENSMUSG00000047363	Cstad	4.062	5.42E-04	1.63E-02
ENSMUSG00000025094	Slc18a2	4.066	2.28E-03	4.47E-02
ENSMUSG00000037754	Ppp1r16b	4.076	2.70E-05	1.90E-03
ENSMUSG00000054675	Tmem119	4.082	2.65E-03	4.96E-02
ENSMUSG00000027004	Frzb	4.112	1.61E-03	3.53E-02
ENSMUSG00000101378	Gm28086	4.132	2.77E-04	1.01E-02
ENSMUSG00000020467	Efemp1	4.145	1.90E-04	7.77E-03
ENSMUSG00000027347	Rasgrp1	4.166	1.43E-03	3.26E-02

ENSMUSG00000037129	Tmprss13	4.250	1.02E-04	5.08E-03
ENSMUSG00000102979	Gm37350	4.255	6.81E-05	3.78E-03
ENSMUSG00000038453	Srcin1	4.274	8.73E-05	4.60E-03
ENSMUSG00000013653	1810065E05	4.299	2.43E-03	4.67E-02
ENSMUSG00000030935	Acsm3	4.302	1.11E-03	2.74E-02
ENSMUSG00000075410	Prcd	4.306	5.66E-07	1.04E-04
ENSMUSG00000041329	Atp1b2	4.309	3.44E-04	1.19E-02
ENSMUSG00000112220	Gm36208	4.316	1.32E-03	3.08E-02
ENSMUSG00000029650	Slc46a3	4.326	9.60E-05	4.84E-03
ENSMUSG00000001827	Folr1	4.330	3.58E-04	1.22E-02
ENSMUSG00000042249	Grk3	4.330	1.81E-03	3.78E-02
ENSMUSG00000074218	Cox7a1	4.333	8.36E-10	5.39E-07
ENSMUSG00000074678	Defb25	4.335	1.57E-03	3.48E-02
ENSMUSG00000074594	Wfdc9	4.356	5.98E-04	1.76E-02
ENSMUSG00000025176	Hoga1	4.386	1.14E-03	2.77E-02
ENSMUSG00000084915	C230037L18	4.388	2.55E-03	4.83E-02
ENSMUSG00000085354	Gm2044	4.406	1.41E-04	6.22E-03
ENSMUSG00000023885	Thbs2	4.413	1.61E-04	6.85E-03
ENSMUSG00000089671	Gm16537	4.424	3.65E-04	1.23E-02
ENSMUSG00000024747	Aldh1a7	4.456	4.84E-08	1.42E-05
ENSMUSG00000026241	Nppc	4.509	2.14E-03	4.25E-02
ENSMUSG00000037206	Islr	4.536	1.39E-04	6.13E-03
ENSMUSG00000040569	Slc26a7	4.538	1.65E-03	3.58E-02
ENSMUSG00000073821	8030451A03	4.555	4.47E-05	2.77E-03
ENSMUSG00000110489	Gm31659	4.576	3.16E-04	1.11E-02
ENSMUSG00000102923	Gm31728	4.602	1.32E-03	3.08E-02
ENSMUSG00000107689	Gm44386	4.609	1.82E-11	1.85E-08
ENSMUSG00000083222	Gm12427	4.620	1.50E-03	3.36E-02
ENSMUSG00000074207	Adh1	4.653	8.90E-05	4.64E-03
ENSMUSG00000031789	Cngb1	4.655	3.08E-06	3.90E-04
ENSMUSG00000046807	Lrrc75b	4.701	1.92E-03	3.91E-02
ENSMUSG00000070304	Scn2b	4.709	2.57E-05	1.84E-03
ENSMUSG00000074604	Mgst2	4.757	8.09E-06	7.59E-04
ENSMUSG00000056174	Col8a2	4.782	5.27E-04	1.59E-02
ENSMUSG00000065952	C330021F23	4.842	8.23E-04	2.20E-02
ENSMUSG00000020096	Tbata	4.846	2.14E-05	1.58E-03
ENSMUSG00000087612	A230005M16	4.852	7.89E-05	4.21E-03
ENSMUSG00000035407	Kank4	4.854	2.68E-07	5.57E-05
ENSMUSG00000086909	Gm4926	4.868	9.03E-05	4.65E-03
ENSMUSG00000072941	Sod3	4.889	9.98E-06	8.65E-04
ENSMUSG00000101438	Gm19412	4.902	2.03E-03	4.09E-02
ENSMUSG00000037709	Fam13a	4.930	6.16E-06	6.17E-04
ENSMUSG00000048038	Ccdc187	4.950	2.89E-04	1.04E-02
ENSMUSG00000021416	Eci3	4.979	1.44E-06	2.19E-04
ENSMUSG00000027870	Hao2	5.082	4.61E-06	4.88E-04
ENSMUSG00000043664	Tmem221	5.101	3.97E-04	1.31E-02
ENSMUSG00000100022	Gm29590	5.150	9.24E-04	2.41E-02

ENSMUSG00000031872	Bean1	5.198	5.99E-06	6.06E-04
ENSMUSG00000066361	Serpina3c	5.198	1.27E-03	3.01E-02
ENSMUSG00000093479	Gm20629	5.207	1.59E-03	3.51E-02
ENSMUSG00000024245	Tmem178	5.245	3.80E-06	4.24E-04
ENSMUSG00000055430	Nap1l5	5.262	8.63E-06	7.95E-04
ENSMUSG00000092323	BB365896	5.302	3.75E-04	1.26E-02
ENSMUSG00000029343	Crybb1	5.305	1.21E-03	2.90E-02
ENSMUSG00000027871	Hsd3b1	5.315	5.54E-16	1.78E-12
ENSMUSG00000023153	Tmem52	5.345	7.32E-04	2.03E-02
ENSMUSG00000097717	1700066J03l	5.470	6.47E-04	1.86E-02
ENSMUSG00000086813	Gm13657	5.551	1.01E-04	5.04E-03
ENSMUSG00000113965	A730091E23	5.577	6.59E-04	1.89E-02
ENSMUSG00000044062	Plekhd1os	5.577	6.82E-04	1.94E-02
ENSMUSG00000113598	Gm48287	5.583	4.39E-05	2.74E-03
ENSMUSG00000069385	Gm10267	5.617	2.27E-03	4.45E-02
ENSMUSG00000020475	Pgam2	5.640	1.96E-04	7.93E-03
ENSMUSG00000112736	Gm48718	5.690	9.33E-05	4.75E-03
ENSMUSG00000020562	Efcab10	5.702	1.06E-04	5.19E-03
ENSMUSG00000063594	Gng8	5.747	1.52E-03	3.38E-02
ENSMUSG00000001985	Grik3	5.780	3.30E-06	3.97E-04
ENSMUSG00000116573	AC161816.1	5.782	7.31E-04	2.03E-02
ENSMUSG00000084942	7330404K18	5.827	9.01E-05	4.65E-03
ENSMUSG00000047786	Lix1	5.872	9.89E-06	8.61E-04
ENSMUSG00000034488	Edil3	5.962	8.81E-05	4.61E-03
ENSMUSG00000104020	Gm37215	6.082	9.44E-04	2.45E-02
ENSMUSG00000085876	Gm12409	6.095	4.07E-04	1.33E-02
ENSMUSG00000041193	Pla2g5	6.134	1.00E-04	5.01E-03
ENSMUSG00000077058	Gm24542	6.153	2.45E-05	1.78E-03
ENSMUSG00000074596	Spint3	6.166	7.16E-05	3.95E-03
ENSMUSG00000069814	Ccdc92b	6.265	5.51E-05	3.29E-03
ENSMUSG00000107876	Gm43936	6.266	6.30E-06	6.22E-04
ENSMUSG00000027584	Oprl1	6.339	4.83E-04	1.49E-02
ENSMUSG00000038354	Ankrd35	6.402	1.07E-06	1.71E-04
ENSMUSG00000085419	Gm11734	6.538	8.23E-07	1.40E-04
ENSMUSG00000085121	5730437C11	6.584	9.14E-05	4.69E-03
ENSMUSG00000104420	E230020A03	6.674	2.50E-04	9.45E-03
ENSMUSG00000113974	4930442G10	6.811	3.43E-05	2.26E-03
ENSMUSG00000030249	Abcc9	6.819	3.04E-06	3.86E-04
ENSMUSG00000113744	Gm47925	7.216	3.57E-06	4.13E-04
ENSMUSG00000040026	Saa3	7.399	2.22E-05	1.62E-03
ENSMUSG00000104109	Gm30292	7.424	3.60E-06	4.13E-04
ENSMUSG00000021613	Hapln1	7.526	7.49E-05	4.09E-03
ENSMUSG00000075010	AW112010	8.355	5.20E-04	1.58E-02
ENSMUSG00000041550	Serpina5	8.604	9.53E-06	8.49E-04
ENSMUSG00000002324	Rec8	8.619	2.02E-06	2.83E-04
ENSMUSG00000041445	Mmrn2	19.080	5.46E-05	3.27E-03
ENSMUSG00000020928	Higd1b	19.494	9.35E-04	2.43E-02
